# Randomized clinical trials in dentistry: Risks of bias, risks of random errors, reporting quality, and methodologic quality over the years 1955–2013

**DOI:** 10.1371/journal.pone.0190089

**Published:** 2017-12-22

**Authors:** Humam Saltaji, Susan Armijo-Olivo, Greta G. Cummings, Maryam Amin, Carlos Flores-Mir

**Affiliations:** 1 School of Dentistry, University of Alberta, Edmonton, Alberta, Canada; 2 Faculty of Rehabilitation Medicine, University of Alberta, Edmonton, Alberta, Canada; 3 Faculty of Nursing, University of Alberta, Edmonton, Alberta, Canada; Copenhagen University Hospital, DENMARK

## Abstract

**Objectives:**

To examine the risks of bias, risks of random errors, reporting quality, and methodological quality of randomized clinical trials of oral health interventions and the development of these aspects over time.

**Methods:**

We included 540 randomized clinical trials from 64 selected systematic reviews. We extracted, in duplicate, details from each of the selected randomized clinical trials with respect to publication and trial characteristics, reporting and methodologic characteristics, and Cochrane risk of bias domains. We analyzed data using logistic regression and Chi-square statistics.

**Results:**

Sequence generation was assessed to be inadequate (at unclear or high risk of bias) in 68% (n = 367) of the trials, while allocation concealment was inadequate in the majority of trials (n = 464; 85.9%). Blinding of participants and blinding of the outcome assessment were judged to be inadequate in 28.5% (n = 154) and 40.5% (n = 219) of the trials, respectively. A sample size calculation before the initiation of the study was not performed/reported in 79.1% (n = 427) of the trials, while the sample size was assessed as adequate in only 17.6% (n = 95) of the trials. Two thirds of the trials were not described as double blinded (n = 358; 66.3%), while the method of blinding was appropriate in 53% (n = 286) of the trials. We identified a significant decrease over time (1955–2013) in the proportion of trials assessed as having inadequately addressed methodological quality items (P < 0.05) in 30 out of the 40 quality criteria, or as being inadequate (at high or unclear risk of bias) in five domains of the Cochrane risk of bias tool: sequence generation, allocation concealment, incomplete outcome data, other sources of bias, and overall risk of bias.

**Conclusions:**

The risks of bias, risks of random errors, reporting quality, and methodological quality of randomized clinical trials of oral health interventions have improved over time; however, further efforts that contribute to the development of more stringent methodology and detailed reporting of trials are still needed.

## Introduction

Randomized clinical trials are the ideal type of clinical research to examine the effectiveness of treatment interventions in health sciences [1]. The value and significance of a randomized clinical trial depends on the control of potential biases, how rigorously it was conducted, and how thoroughly the results were reported. The SPIRIT (Standard Protocol Items: Recommendations for Interventional Trials) Statement [2], the Consolidated Standards of Reporting Trials (CONSORT) Statement [3], and recent initiatives such as the International Committee of Medical Journal Editors (ICMJE) statement on clinical trial registration [4], have led to improvements in both the methodological and reporting quality of medical randomized clinical trials [5–7]. Adhering to these initiatives is critical to oral health research and practice, as high quality randomized clinical trials contribute largely to the body of evidence measured in systematic reviews and meta-analyses, especially when assessing therapeutic interventions.

Currently, nearly 50 clinical trials of oral health interventions are estimated to be published every month, and this number is expected to increase over time [8]. Emerging evidence from methodological reports published in various dentistry-related specialties over the last decade (periodontics [9], prosthodontics [10], implantology [11], orthodontics [12], restorative dentistry [13], and six dental specialties [14]) report methodological quality of oral heath randomized clinical trials that is below acceptable levels to adequately lead clinical decision making. Moreover, there is evidence that some trials are biased and, due to weaknesses in their methodological characteristics, they tend to exaggerate the magnitude of the treatment effect [15]. This emerging evidence raises questions about the validity of trial results for oral health interventions, which dental practitioners use when making day-to-day clinical decisions in dental practice, and which policy makers use more generally when developing clinical practice guidelines.

In the context of medical research methodology, the internal validity of a trial is dependent on “risk of bias” (which concerns the internal validity of a trial) [16] and “risks of random errors” (i.e., risks of play of chance). Moreover, while “reporting quality” refers to the reporting of the conduct and design of a trial [17, 18], “methodological quality” is related to the internal validity of a trial and determined by the extent to which the conduct and design of a trial are precisely and rigorously performed to generally acceptable standards so that biases are minimized.

In the field of oral health, to our knowledge, no study has assessed changes over time regarding quality of reporting, methodological characteristics, and risks of bias in randomized clinical trials of oral health interventions. A recent report by Reveiz et al. [19] described the results of an examination of different risk of bias domains in a sample of medical randomized clinical trials (identified from a cohort of Cochrane reviews). Reveiz and colleagues’ report stated that the rate of trials found at low risk of bias consistently increased with time. However, since the randomized clinical trials in Reveiz and colleagues’ sample were performed in the field of medicine and were dependent on the risk of bias assessment performed by investigators presenting published reviews (rather than by conducting standardized data extraction from each trial), the findings cannot be compared to findings from trials in the field of dentistry which tend to have different design characteristics, such as difficulty in applying blinding and common use of the split-mouth design.

Consequently, it is unclear if the increase in number of published randomized clinical trials of oral health interventions over time has been associated with changes in the conduct and reporting of the trials. In this study, we set out to assess these aspects in randomized clinical trials of oral health interventions, and if these aspects have improved over time. Our objectives were to (1) examine the risks of bias, risks of random errors, reporting quality, and methodological quality, and the general trial characteristics of randomized clinical trials of oral health interventions; and (2) determine whether (and to what extent) risks of bias, risks of random errors, reporting quality, and methodological quality have improved over time.

## Methods

### Study sample

**Fig 1:**
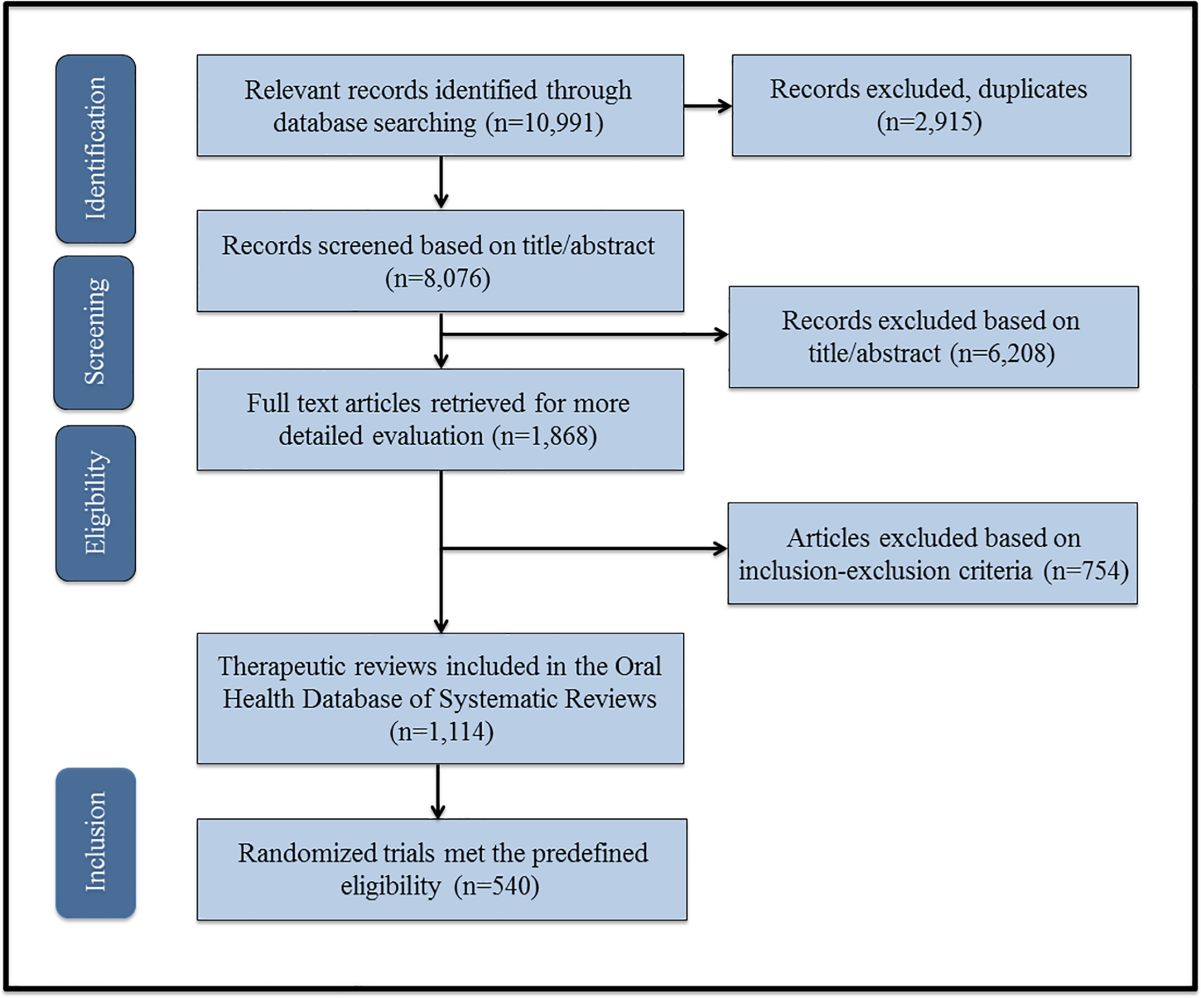
Flow diagram of the literature search [20, 21].

We used the Oral Health Database of Systematic Reviews [20, 21] which includes a comprehensive selection of oral health systematic reviews published in the field of oral health research between 1991 and 2014. From this database, we selected a sample of systematic reviews with meta-analyses and their associated randomized clinical trials that met the following criteria: the meta-analysis was (1) published in any language and (2) conducted in an oral health field that examined an intervention concerning craniofacial, oral, or dental diseases (as defined by the American Dental Association [ADA] scope of practice) [22]. A randomized clinical trial was defined as “an experiment in which two or more interventions (possibly including a control intervention or no intervention) are compared, by being randomly allocated to participants” [16]. Further details regarding the study selection included in the final database of systematic reviews have been published [20, 21] (see **Fig 1**). Briefly, two reviewers (dentists with oral health research backgrounds) independently selected relevant reports and determined the final eligibility of the full texts (any disagreements were resolved through consensus). Ultimately, 540 randomized clinical trials that met the predefined eligibility criteria were selected and utilized in this study. The list of included randomized clinical trials is provided in **S5 Appendix**. The study protocol was not registered or published in advance.

### Data extraction

A panel of five reviewers from diverse health research areas (dentistry, pediatrics, and physical therapy) extracted the data. To ensure consistency during data extraction, two team members (H.S., S.A.) facilitated a reviewer training process, similar to the process followed in our team’s previous investigations [23, 24]. In this process, the review panel evaluated and discussed 10 randomized clinical trials not included in the final set of trials. Once agreement on data extraction protocols and interpretation was achieved, the review panel performed data extraction in duplicate. Two assessors independently carried out data extraction for each included randomized clinical trial (consensus meetings were employed to resolve any disagreements). One assessor (H.S.) who has a background in oral health research performed a complete data extraction (n = 540, 100%), while other members of the review panel (C.H., J.S., J.F.) who have medical (non-oral health) research backgrounds, acted as secondary assessors. If two assessors could not reach an agreement, then a third assessor (S.A.) assisted with consensus. Only data that received consensus were used for data analyses. We used a structured and pilot-tested data extraction template, designed using Microsoft Office Access, for data extraction. We extracted details from each of the selected randomized clinical trials with respect to publication and trial characteristics, risks of bias, risks of random errors, reporting quality, and methodological quality, as described below.

### Publication and trial characteristics

Data elements related to publication and trial characteristics included the following information: publication year, dental speciality as classified by the American Dental Association (ADA) (e.g., periodontics, dental public health, prosthodontics and restorative dentistry, oral medicine and oral pathology, implantology, oral and maxillofacial surgery, orthodontics and dentofacial orthopedics, pediatric dentistry, endodontics [22]), country and continent of first author, number of authors, funding source (e.g., foundation, government, industry, academic), type of journal (e.g., specialty oral health, general oral health, nonoral health), type of intervention (e.g., surgical, drug, dental material, device, psychological, educational, policy), age of participants, number of centres (e.g., multicentres, single centre), design (e.g., parallel, crossover, split-mouth, cluster), type of outcome (e.g., subjective, objective), and sample size.

### Risks of bias

We employed the Cochrane risk of bias tool introduced in 2008 [1], which contains six domains and seven items, namely, “sequence generation,” “allocation concealment,” “blinding of outcome assessors,” “blinding of participants,” “incomplete outcome data,” “selective outcome reporting,” and “other sources of bias.” We used the Cochrane Collaboration guidelines to score domains (e.g., high, low, unclear). However, we developed specific rules to make final decisions (see **S3 Appendix** and **S4 Appendix)**. For “other sources of bias,” we examined baseline comparability, control for cointerventions, whether treatment compliance was acceptable, and funding [25]. For the overall assessment of risk of bias, if *one* domain was assessed as having a high risk, the overall risk of bias assessment was labelled “high risk.” A randomized trial was considered to be at low risk of bias if it was assessed as “low risk” in *all* individual domains. If the assessment was “unclear” in *at least* one domain (and other domains were unclear or low) the overall risk of bias assessment was designated “unclear” [26, 27].When reporting the results of the risk of bias assessment, we combined “high” and “unclear” risk of bias assessments into one type of trials, considering that trials that these types of trials are at risks of overestimating benefits and at risk of underestimating harms [1, 14, 25].

### Risks of random error

Risks of random errors involves two criteria: (1) sample size calculation and (2) adequate sample size (see **S1 Appendix**).

### Reporting quality and methodologic quality

Reporting quality and methodological quality are difficult to distinguish and often overlap to some extent. Methodological quality is defined as “the confidence that the trial design, conduct, and analysis has minimized or avoided biases in its treatment comparisons” [17, 18] (e.g., the sequence generation was appropriate). Reporting quality involves the provision of “information about the design, conduct, and analysis of the trial” [17, 18] (e.g., this was a randomized trial). Accordingly, based on preliminary work performed by the research team [28, 29], we obtained 40 quality assessment criteria and their classifications (“reporting” vs. “conduct”) from the most commonly used quality assessment tools in health care research [30–37]. Of the 40 quality criteria selected, 15 criteria assess “reporting” quality, 21 criteria assess “methodological” quality, and four quality criteria assess both reporting quality and methodological quality. We classified the items that evaluated methodological quality according to type of bias as follows [28, 29] (see **S1 Appendix**): selection bias (6 criteria), performance and detection bias (4 criteria), performance bias (9 criteria), performance and compliance bias (2 criteria), information bias (3 criteria), reporting bias (3 criteria), attrition bias (5 criteria), detection bias (2 criteria), statistical bias (1 criterion), threats to precision (1 criterion), and multiple biases (2 criteria). We also grouped the selected quality criteria according to the following categories [16]: patient selection (inclusion and exclusion criteria, description of participants); assignment, randomization, and allocation concealment; blinding; interventions; attrition, follow up and protocol deviation; outcomes; and statistical analysis. Using original tools as guidelines, the definitions and methods were derived for each criterion, using a three-part answering scheme (yes, no, unclear) for each item. We established decision rules and guidelines to ensure consistency (see **S1 Appendix** and **S2 Appendix**).

### Data analysis

We conducted descriptive analyses for each trial characteristic, quality assessment item, and risk of bias domain (using means and standard deviations [SD] or median and interquartile range [IQR] for continuous outcomes, and proportions and percentages for categorical outcomes, where appropriate). To evaluate whether the quality of randomized clinical trials has improved over years, trials were grouped according to four periods of publication year: before 1990, 1990–1999, 2000–2006, 2007–2013. We used Chi-square statistics and two-tailed Fisher exact tests to examine the difference in proportion with respect to time periods for all quality assessment items and risk of bias domains. Furthermore, we used a logistic regression to explore the relationship between each criterion and time; we entered time into the logistic regression model as a continuous variable (publication year) and a categorical variable (time period of publication year; < 1990 was used as a reference category). The outcome of each analysis was each methodological criterion dichotomized in low risk vs. others (unclear, high risk of bias), or yes vs. others (no, unclear, not-reported). We reported odds ratios (OR) with 95% confidence intervals (CIs) and we set the statistical significance at P < .05. We performed statistical analyses using Stata Version 14.0 (StataCorp) and the Statistical Package for Social Sciences for Windows (SPSS) Version 18.0 (IBM, Armonk, NY).

## Results

### Trial publication and characteristics

**Fig 2:**
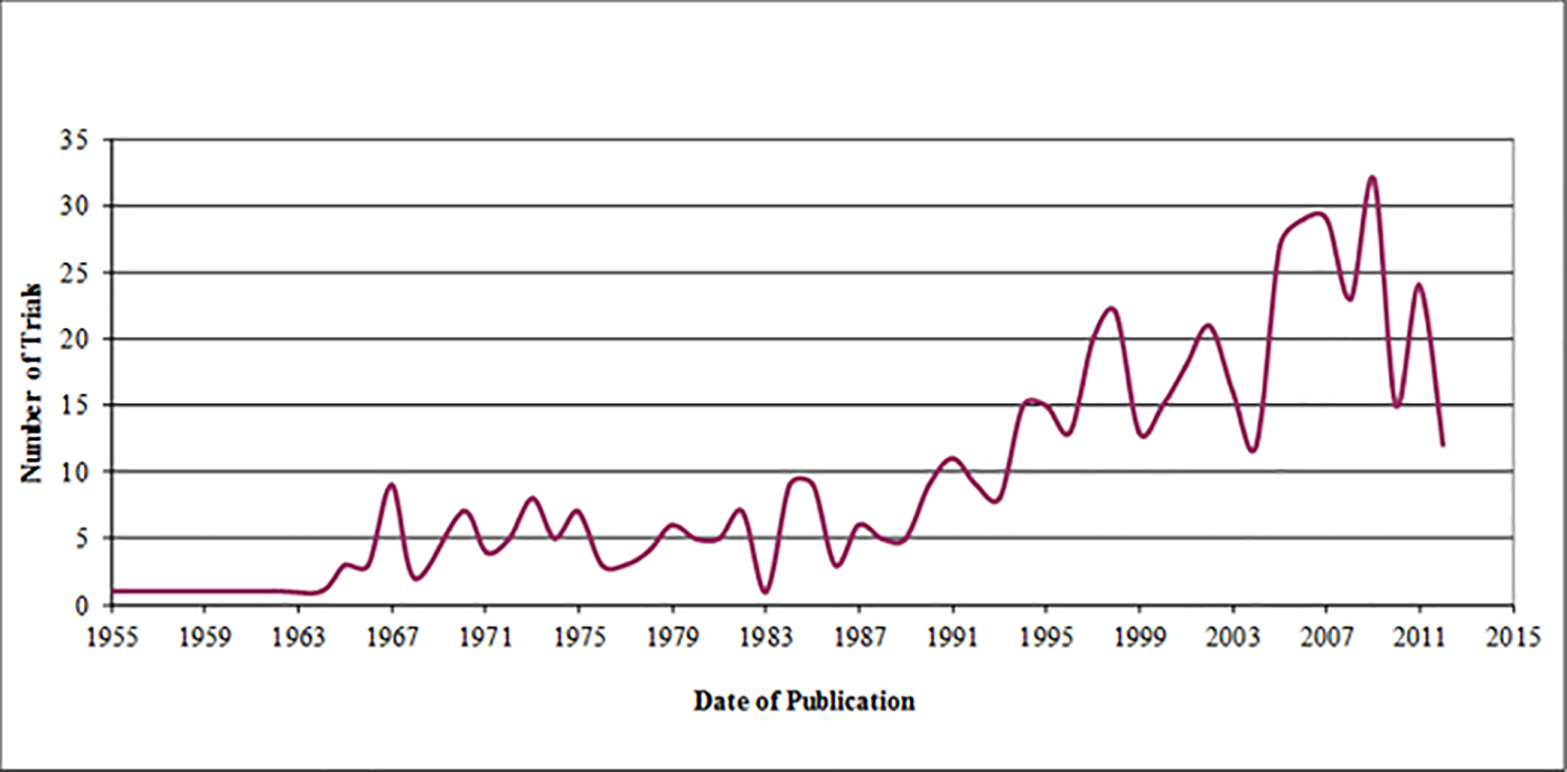
Number of oral heath trials according to year of publication.

From the selected 540 trials published between 1955 and 2013 (median year of publication: 2000; IQR: 1990, 2007) (see **Fig 2**), the majority of trials were published either in periodontics (n = 233; 43.1%), dental public health (n = 124; 23.0%), or prosthodontic and restorative dentistry (n = 54; 10.0%). More than half of the trials were published in journals that specialized in oral health (n = 304; 56.3%) (see **Table 1**). The trials’ first authors were most frequently from Europe (n = 239; 44.3%) followed by North America (n = 202; 37.4%). Three countries (the United Kingdom, Italy, and the United States) accounted for almost half of all trials (n = 280; 51.9%). Approximately one fifth of the trials were multicenter trials, nearly half of the trials involved four to six authors (n = 249; 46.1%), and one third included two to three authors (n = 169; 31.3%). In approximately half of the trials, the authors did not declare whether they received a source of funding (n = 256; 47.4%), while nearly one third of trials received funding from industry (n = 171; 31.7%).

**Table 1:** Publication and trial characteristics of trials (N = 540).

Trial Characteristic		No. (%)
**Primary dental specialty**		
Periodontics		233 (43.1)
Dental public health		124 (23.0)
Prosthodontics and restorative dentistry		54 (10.0)
Oral medicine and oral pathology		42 (7.8)
Implantology		33 (6.1)
Oral and maxillofacial surgery		31 (5.7)
Orthodontics and dentofacial orthopedics		13 (2.4)
Pediatric dentistry		6 (1.1)
Endodontics		4 (0.7)
**Date of publication**		
Before 1990		127 (23.5)
1990–1999		135 (25.0)
2000–2006		138 (25.6)
2007–2013		140 (25.9)
**Continent of first author**		
Europe		239 (44.3)
North America		202 (37.4)
Asia		55 (10.2)
South America		28 (5.2)
Africa		7 (1.3)
Australia		9 (1.7)
**Country of first author** *(No*. *of countries = 45)*		
USA		187 (34.6)
UK		53 (9.8)
Italy		40 (7.4)
Sweden		27 (5.0)
Turkey		26 (4.8)
Brazil		25 (4.6)
Germany		20 (3.7)
Canada		16 (3.0)
France		13 (2.4)
China		12 (2.2)
Other		121 (22.4)
**Number of authors**		
1		25 (4.6)
2–3		169 (31.3)
4–6		249 (46.1)
> or equal to 7		97 (18.0)
**Source of funding**		
Industry		156 (28.9)
Government		43 (8.0)
Academics		19 (3.5)
Foundation		14 (2.6)
Government and foundation/academics		17 (3.1)
Industry and government/academics		15 (2.8)
Other combination		13 (2.4)
No funding		7 (1.3)
Funding not declared		256 (47.4)
**Type of journal**		
Specialty oral-health		304 (56.3)
General oral-health		171 (31.7)
Non-oral-health (medical)		65 (12.0)
**Study design**		
Parallel		372 (68.9)
Split-mouth		126 (23.3)
Crossover		28 (5.2)
Cluster		10 (1.9)
Factorial		4 (0.7)
**Placebo-controlled**		
Yes		204 (37.8)
No		336 (62.2)
**Number of centers**		
Multicenter		103 (19.1)
	2–5 center	51 (9.4)
	6–10 center	28 (5.2)
	>10 center	24 (4.4)
Single center		393 (72.8)
Unclear		44 (8.1)
**Nature of intervention, classification I**		
Drug		143 (26.5)
Non-drug		359 (66.5)
Both (drug and non-drug)		38 (7.0)
**Nature of intervention, classification II**		
Surgical		158 (29.3)
Non-surgical		370 (68.5)
Both (surgical and non-surgical)		12 (2.2)
**Nature of intervention, classification III**		
Drug		170 (31.5)
Surgical		163 (30.2)
Dental material		83 (15.4)
Device		35 (6.5)
Psychological, Educational, Policy		16 (3.0)
Other		73 (13.5)
**Mean age of participants**		
Pediatric		136 (25.2)
Adult		398 (73.7)
Geriatric		6 (1.1)

Approximately one third of the trials were placebo-clinical (n = 204; 37.8%) and two thirds examined nondrug (n = 359; 66.5%) or nonsurgical (n = 370; 68.5%) interventions. One quarter of the trials examined pediatric patients (n = 136; 25.2%), while the majority examined adults (n = 398; 73.7%). The majority of trials used parallel design (n = 372; 68.9%), while almost one quarter used the split-mouth design (n = 126; 23.3%).

### Changes in risks of bias, risks of random errors, reporting quality, and methodological characteristics over time

Sequence generation was assessed to be adequate (low risk of bias) in 32% (n = 173) of the trials, while it was inadequate (unclear or high risk of bias) in 68% (n = 367) of the trials. Allocation concealment was inadequate (unclear or high risk of bias) in the majority of trials (n = 464; 85.9%). Blinding of participants was judged to be adequate (at low risk of bias) in 71.5% (n = 386) of the trials, and blinding of the outcome assessment was judged to be adequate (at low risk of bias) in 59.4% of the trials. Other sources of bias—baseline comparability, similarity of cointerventions, and compliance to treatment—were judged at low risk of bias in 77.8%, 40.2%, and 53.5% of the trials, respectively. The influence of the trial sponsor was assessed as being inadequate (unclear or high risk of bias) in 83.3% (n = 450) of the trials, while it was assessed as appropriate in 16.7% (n = 90) of the trials. The overall risk of bias was inadequate (unclear or high risk of bias) in 94.6% (n = 511) of the trials, and an overall low risk of bias was assessed in only 5.4% (n = 29) of the trials (see **Table 2**).

**Table 2:** Risk of bias, risk of random error, and quality assessments by criterion (N = 540).

**Criterion**		**Risk of Bias Assessment, N (%)**		
		**Low Risk**	**High Risk**	**Unclear Risk**
Sequence generation		173 (32)	2 (0.4)	365 (67.6)
Allocation concealment		76 (14.1)	6 (1.1)	458 (84.8)
Blinding of participants		386 (71.5)	7 (1.3)	147 (27.2)
Blinding of outcome assessment		321 (59.4)	16 (3.0)	203 (37.6)
Incomplete outcome data		295 (54.6)	93 (17.2)	152 (28.1)
Selective outcome reporting		519 (96.1)	5 (0.9)	16 (3.0)
Other sources of bias		286 (53.0)	1 (0.2)	253 (46.9)
	Baseline comparability	420 (77.8)	0 (0.0)	120 (22.2)
	Similarity of co-interventions	217 (40.2)	0 (0.0)	323 (59.8)
	Compliance to the treatment	289 (53.5)	1 (0.2)	250 (46.3)
	Appropriate influence of trial sponsor	90 (16.7)	57 (10.6)	393 (72.8)
	Early stopping of trial	5 (0.9)	535 (99.1)	0 (0.0)
Overall risk of bias		29 (5.4)	113 (20.9)	398 (73.7)
		**Risk of Random Error, N (%)**		
		**Low Risk**	**High Risk**	**Unclear Risk**
Sample size calculation done prior to study initiation		113 (20.9)	420 (77.8)	7 (1.3)
Adequate sample size		95 (17.6)	18 (3.3)	427 (79.1)
		**Quality Assessment, N (%)**		
		**Yes**	**No**	**Unclear/NR**
**Patient Selection (Inclusion and Exclusion and Description of Participants)**				
Inclusion criteria clearly defined		497 (92)	7 (1.3)	36 (6.7)
Exclusion criteria clearly defined		486 (90)	24 (4.4)	30 (5.6)
Baseline comparability (group equivalence)		393 (72.8)	3 (0.6)	144 (26.7)
**Assignment, Randomization, and Allocation Concealment**				
Study described as randomized		517 (95.7)	16 (3.0)	7 (1.3)
Generation of allocation sequence appropriate		181 (33.5)	8 (1.5)	351 (65)
Generation of allocation sequence concealed		64 (11.9)	7 (1.3)	469 (86.9)
**Blinding**				
Study described as double-blind		181 (33.5)	358 (66.3)	1 (0.2)
Method of blinding appropriate		286 (53)	17 (3.1)	237 (43.9)
Blinding of principal investigator		33 (6.1)	10 (1.9)	497 (92.0)
Blinding of assessor		321 (59.4)	16 (3.0)	203 (37.6)
Blinding of patients		192 (35.6)	69 (12.8)	279 (51.7)
Blinding of therapists/care-providers		134 (24.8)	356 (65.9)	50 (9.3)
Blinding of data analyst		9 (1.7)	3 (0.6)	528 (97.8)
**Interventions**				
Treatment protocol adequately described for treatment group		532 (98.5)	2 (0.4)	6 (1.1)
Treatment protocol adequately described for control group		528 (97.8)	4 (0.7)	8 (1.5)
Treatment protocol adequately described for comparison group ^†^		227 (98.7)	1 (0.4)	2 (0.9)
Presence of a control group		395 (73.1)	143 (26.5)	2 (0.4)
Presence of a placebo group		206 (38.1)	334 (61.9)	0 (0.0)
Co-interventions avoided/comparable		208 (38.5)	4 (0.7)	328 (60.7)
Co-interventions reported for each group §		77 (15.3)	424 (84.1)	3 (0.6)
Testing of participants compliance to treatment protocol		330 (61.1)	11 (2)	199 (36.9)
Compliance acceptable (80% of treatment received)		275 (50.9)	5 (0.9)	260 (48.1)
**Attrition, Follow-up and Protocol Deviation**				
Report of withdraws and dropouts		483 (89.4)	20 (3.7)	37 (6.9)
Withdrawal/dropouts rate acceptable (< than 20%)		395 (73.1)	93 (17.2)	52 (9.6)
Reasons for withdraws/dropouts reported		384 (71.1)	109 (20.2)	47 (8.7)
Adverse effects described		276 (51.1)	259 (48.0)	5 (0.9)
Short follow-up measurement performed		509 (94.3)	31 (5.7)	0 (0.0)
Long term follow-up measurement performed		307 (68.7)	140 (31.3)	0 (0.0)
**Outcomes**				
Outcome measures described		528 (97.8)	6 (1.1)	6 (1.1)
Validity for main outcome measures reported		18 (3.3)	522 (96.7)	0 (0.0)
Reliability for main outcome measures reported		37 (6.9)	503 (93.1)	0 (0.0)
Responsiveness for main outcome measures reported		17 (3.1)	522 (96.7)	1 (0.2)
**Data Analysis**				
Descriptive measures identified and reported		534 (98.9)	4 (0.7)	2 (0.4)
Appropriate statistical analysis used		463 (85.7)	4 (0.7)	73 (13.5)
Intention to treat analysis used		218 (40.4)	264 (48.9)	58 (10.7)
Clinical significance reported		157 (29.1)	381 (70.7)	1 (0.2)

^†^ Does not equal 100% for overall, as the item was not applicable in 310 trials.

§Does not equal 100% for overall, as the item was not applicable in 36 trials.

Regarding risks of random errors, a sample size calculation before the initiation of the study was not performed or reported in 79.1% (n = 427) of the trials, while the sample size was assessed as adequate in only 17.6% (n = 95) of the trials.

Inclusion and exclusion criteria were clearly defined in the majority of trials, while baseline comparability was adequate in 72.8% of the trials. Two thirds of the trials were not described as double blinded (n = 358; 66.3%), while the method of blinding was appropriate in 53% (n = 286) of the trials. Blinding of the principal investigator and statistician was unclear/not reported in 92% and 97.8% of the trials, respectively, that is, in the vast majority of the trials.

The treatment protocol was adequately described for treatment and control groups in the vast majority of trials, with 73.1% (n = 395) having a designated control group, and 38.1% (n = 206) using a placebo group. Whether cointerventions were avoided/comparable was assessed as unclear/not reported in 60.7% (n = 328) of the trials, while 84.1% (n = 424) of the trials did not report cointerventions for each group. Participants compliance to treatment protocol was tested in 61.1% (n = 330) of the trials, with compliance being acceptable (more than or equal to 80%) in 50.9% (n = 275) of the trials. Withdrawals/dropouts were reported in the vast majority (89.4%, n = 483) of trials, with withdrawal/dropout rates being acceptable (less than or equal to 20%) in 73.1% (n = 395) of the trials, and reasons for withdrawals/dropouts reported in 71.1% (n = 384) of the trials. Adverse effects were not described in nearly half (n = 264; 48.9%) of the trials.

Outcome measures were described in the majority of trials, while psychometric properties of main outcome measures—validity, reliability, and responsiveness—were not reported in 96.7%, 93.1%, and 96.7% of the trials, respectively. The statistical analysis was appropriate in 85.7% (n = 463) of the trials, with descriptive measures being reported in the majority of the trials. The clinical significance was not reported in 70.9% (n = 382) of the trials, while the intention to treat analysis was not used/reported in 59.6% (n = 264) of the trials (see **Table 2**).

We identified a significant decrease (P < 0.001) in the proportion of trials judged as being inadequate (having high or unclear risk of bias) over time in five domains of the Cochrane risk of bias tool: sequence generation, allocation concealment, incomplete outcome data, other sources of bias (including: baseline comparability, similarity of cointerventions, and compliance to treatment), and overall risk of bias (see **Table 3**). The proportion of trials assessed as being inadequate (having high or unclear risk of bias), with respect to patient blinding, decreased significantly (P < 0.031) in the sample, while change in blinding of outcome assessment and selective outcome reporting were not statistically significant.

**Table 3:** Risk of bias assessment by domain over time (N = 540), N (%).

Domain	Judgment	<1990	1990–1999	2000–2006	2007–2013	*P* value
**Sequence generation**	Low risk	15 (11.8)	27 (2)	59 (42.7)	72 (51.4)	<0.001
	High risk	1 (0.7)	0 (0.0)	0 (0.0)	1 (0.7)	
	Unclear risk	111 (87.4)	108 (8)	79 (57.3)	67 (47.9)	
**Allocation concealment**	Low risk	6 (4.7)	16 (11.9)	26 (18.8)	39 (27.8)	<0.001
	High risk	1 (0.8)	1 (0.7)	1 (0.7)	3 (2.1)	
	Unclear risk	120 (94.5)	118 (87.4)	111 (80.4)	98 (70)	
**Blinding of participants**	Low risk	83 (65.3)	96 (71.1)	97 (70.3)	110 (78.6)	0.031
	High risk	0 (0.0)	0 (0.0)	4 (2.9)	3 (2.1)	
	Unclear risk	44 (34.6)	39 (28.9)	37 (26.8)	27 (19.3)	
**Blinding of outcome assessment**	Low risk	78 (61.4)	70 (51.9)	86 (62.3)	87 (62.1)	0.052
	High risk	0 (0.0)	3 (2.2)	6 (4.3)	7 (5)	
	Unclear risk	49 (38.6)	62 (45.9)	46 (33.3)	46 (32.9)	
**Incomplete outcome data**	Low risk	18 (14.2)	82 (60.7)	98 (71.0)	97 (69.3)	<0.001
	High risk	61 (48.0)	10 (7.4)	14 (10.1)	8 (5.7)	
	Unclear risk	48 (37.8)	43 (31.9)	26 (18.8)	35 (25)	
**Selective outcome reporting**	Low risk	118 (92.9)	130 (96.3)	136 (98.6)	135 (96.4)	0.335
	High risk	3 (2.4)	1 (0.7)	0 (0.0)	1 (0.7)	
	Unclear risk	6 (4.7)	4 (3)	2 (1.4)	4 (2.9)	
**Other sources of bias**	Low risk	20 (15.7)	77 (57)	93 (67.4)	96 (68.6)	<0.001
	High risk	1 (0.8)	0 (0.0)	0 (0.0)	0 (0.0)	
	Unclear risk	106 (83.5)	58 (43)	45 (32.6)	44 (31.4)	
**Baseline comparability**	Low risk	68 (53.5)	114 (84.4)	121 (87.7)	117 (83.6)	<0.001
	High risk	0 (0.0)	0 (0.0)	0 (0.0)	0 (0.0)	
	Unclear risk	59 (46.5)	21 (15.6)	17 (12.3)	23 (16.4)	
**Similarity of co-interventions**	Low risk	17 (13.4)	60 (44.4)	70 (50.7)	70 (50)	<0.001
	High risk	0 (0.0)	0 (0.0)	0 (0.0)	0 (0.0)	
	Unclear risk	110 (86.6)	75 (55.6)	68 (49.3)	70 (50)	
**Compliance to the treatment**	Low risk	23 (18.1)	75 (55.6)	91 (65.9)	100 (71.4)	<0.001
	High risk	1 (0.8)	0 (0.0)	0 (0.0)	0 (0.0)	
	Unclear risk	103 (81.1)	60 (44.4)	47 (34.1)	40 (28.6)	
**Appropriate influence of trial sponsor**	Low risk	14 (11)	17 (12.6)	20 (14.5)	39 (27.9)	0.001
	High risk	4 (3.1)	21 (15.6)	20 (14.5)	12 (8.6)	
	Unclear risk	109 (85.8)	97 (71.9)	98 (71)	89 (63.6)	
**Early stopping of trial**	Low risk	2 (1.6)	0 (0.0)	2 (1.4)	1 (0.7)	0.508
	High risk	125 (98.4)	135 (100)	136 (98.6)	139 (99.3)	
	Unclear risk	0 (0.0)	0 (0.0)	0 (0.0)	0 (0.0)	
**Overall risk of bias**	Low risk	0 (0.0)	6 (4.4)	9 (6.5)	14 (10)	<0.001
	High risk	69 (54.3)	14 (10.4)	20 (14.5)	10 (7.1)	
	Unclear risk	58 (45.7)	115 (85.2)	109 (79)	116 (82.9)	

The proportion of trials assessed as having inadequately addressed methodological quality items decreased significantly over time in 30 out of the 40 quality criteria (23 quality criteria at P < 0.001, seven quality criteria at P < 0.05). This was not statistically significant in the following items: study described as randomized, method of blinding appropriate, blinding of principle investigator, blinding of assessor, treatment protocol adequately described for the treatment group and for the comparison group, report of withdrawals/dropouts, outcome measures described, validity and responsiveness for main outcome measures reported, descriptive measures reported, and early cessation of a trial (see **Table 4**).

**Table 4:** Quality assessment and risk of random error by item over time (N = 540), N (%).

Criterion	Judgment	<1990	1990–1999	2000–2006	2007–2013	*P*-value
**Patient Selection (Inclusion and Exclusion and Description of Participants)**						
**Inclusion criteria clearly defined**	Yes	102 (80.3)	122 (90.4)	135 (97.8)	138 (98.6)	<0.001
	No	2 (1.6)	4 (3)	1 (0.7)	0 (0.0)	
	Unclear/NR	23 (18.1)	9 (6.7)	2 (1.4)	2 (1.4)	
**Exclusion criteria clearly defined**	Yes	100 (78.7)	117 (86.7)	133 (96.4)	136 (97.1)	<0.001
	No	13 (10.2)	10 (7.4)	1 (0.7)	0 (0.0)	
	Unclear/NR	14 (11)	8 (5.9)	4 (2.9)	4 (2.9)	
**Baseline comparability (group equivalence)**	Yes	70 (55.1)	104 (77)	112 (81.2)	107 (76.4)	<0.001
	No	3 (2.4)	0 (0.0)	0 (0.0)	0 (0.0)	
	Unclear/NR	54 (42.5)	31 (23)	26 (18.8)	33 (23.6)	
**Assignment, Randomization, and Allocation Concealment**						
**Study described as randomized**	Yes	119 (93.7)	125 (92.6)	133 (96.4)	140 (100)	0.080
	No	6 (4.7)	7 (5.2)	3 (2.2)	0 (0.0)	
	Unclear/NR	2 (1.6)	3 (2.2)	2 (1.4)	0 (0.0)	
**Generation of allocation sequence appropriate**	Yes	18 (14.2)	32 (23.7)	58 (42)	73 (52.1)	<0.001
	No	4 (3.1)	2 (1.5)	2 (1.4)	0 (0.0)	
	Unclear/NR	105 (82.7)	101 (74.8)	78 (56.5)	67 (47.9)	
**Generation of allocation sequence concealed**	Yes	3 (2.4)	12 (8.9)	19 (13.8)	30 (21.4)	<0.001
	No	1 (0.8)	1 (0.7)	3 (2.2)	2 (1.4)	
	Unclear/NR	123 (96.9)	122 (90.4)	116 (84.1)	108 (77.1)	
**Blinding**						
**Study described as double blind**	Yes	61 (48)	48 (35.6)	30 (21.7)	42 (30.0)	<0.001
	No	66 (52)	87 (64.4)	107 (77.5)	98 (70.0)	
	Unclear/NR	0 (0.0)	0 (0.0)	1 (0.7)	0 (0.0)	
**Method of blinding appropriate**	Yes	74 (58.3)	65 (48.1)	68 (49.3)	79 (56.4)	0.066
	No	0 (0.0)	3 (2.2)	8 (5.8)	6 (4.3)	
	Unclear/NR	53 (41.7)	67 (49.6)	62 (44.9)	55 (39.3)	
**Blinding of principal investigator**	Yes	9 (7.1)	3 (2.2)	8 (5.8)	13 (9.3)	0.105
	No	1 (0.8)	3 (2.2)	1 (0.7)	5 (3.6)	
	Unclear/NR	117 (92.1)	129 (95.6)	129 (93.5)	122 (87.1)	
**Blinding of assessor**	Yes	78 (61.4)	69 (51.1)	86 (62.3)	87 (62.1)	0.038
	No	0 (0.0)	3 (2.2)	6 (4.3)	7 (5)	
	Unclear/NR	49 (38.6)	63 (46.7)	46 (33.3)	46 (32.9)	
**Blinding of participants /patients**	Yes	62 (48.8)	43 (31.9)	36 (26.1)	51 (36.4)	0.005
	No	10 (7.9)	20 (14.8)	24 (17.4)	15 (10.7)	
	Unclear/NR	55 (43.3)	72 (53.3)	78 (56.5)	74 (52.9)	
**Blinding of therapists /care- providers**	Yes	56 (44.1)	35 (25.9)	22 (15.9)	21 (15)	<0.001
	No	42 (33.1)	93 (68.9)	106 (76.8)	115 (82.1)	
	Unclear/NR	29 (22.8)	7 (5.2)	10 (7.2)	4 (2.9)	
**Blinding of data analyst**	Yes	0 (0.0)	0 (0.0)	2 (1.4)	7 (5)	0.020
	No	1 (0.8)	1 (0.7)	0 (0.0)	1 (0.7)	
	Unclear/NR	126 (99.2)	134 (99.3)	136 (98.6)	132 (94.3)	
**Interventions**						
**Treatment protocol adequately described for treatment group**	Yes	123 (96.9)	132 (97.8)	137 (99.3)	140 (100)	0.201
	No	1 (0.8)	0 (0.0)	1 (0.7)	0 (0.0)	
	Unclear/NR	3 (2.4)	3 (2.2)	0 (0.0)	0 (0.0)	
**Treatment protocol adequately described for control group**	Yes	120 (94.5)	132 (97.8)	136 (98.6)	140 (100)	0.028
	No	2 (1.6)	0 (0.0)	2 (1.4)	0 (0.0)	
	Unclear/NR	5 (3.9)	3 (2.2)	0 (0.0)	0 (0.0)	
**Treatment protocol adequately described for comparison group**	Yes	78 (97.5)	58 (98.3)	45 (100)	46 (100)	0.352
	No	0 (0.0)	1 (1.7)	0 (0.0)	0 (0.0)	
	Unclear/NR	2 (2.5)	0 (0.0)	0 (0.0)	0 (0.0)	
**Presence of a control group**	Yes	65 (51.2)	98 (72.6)	116 (84.1)	116 (82.9)	<0.001
	No	62 (48.8)	36 (26.7)	22 (15.9)	23 (16.4)	
	Unclear/NR	0 (0.0)	1 (0.7)	0 (0.0)	1 (0.7)	
**Presence of a placebo group**	Yes	88 (69.3)	51 (37.8)	33 (23.9)	34 (24.3)	<0.001
	No	39 (30.7)	84 (62.2)	105 (76.1)	106 (75.7)	
	Unclear/NR	0 (0.0)	0 (0.0)	0 (0.0)	0 (0.0)	
**Co-interventions avoided/comparable**	Yes	16 (12.6)	56 (41.5)	71 (51.4)	65 (46.4)	<0.001
	No	0 (0.0)	1 (0.7)	1 (0.7)	2 (1.4)	
	Unclear/NR	111 (87.4)	78 (57.8)	66 (47.8)	73 (52.1)	
**Co-interventions reported for each group separately**	Yes	5 (4)	22 (18.2)	24 (18.6)	26 (20.2)	<0.001
	No	120 (96)	99 (81.8)	102 (79.1)	103 (79.8)	
	Unclear/NR	0 (0.0)	0 (0.0)	3 (2.3)	0 (0.0)	
**Testing of participants compliance to treatment protocol**	Yes	43 (33.9)	90 (66.7)	94 (68.1)	103 (73.6)	<0.001
	No	0 (0.0)	2 (1.5)	3 (2.2)	6 (4.3)	
	Unclear/NR	84 (66.1)	43 (31.9)	41 (29.7)	31 (22.1)	
**Compliance acceptable (80% of treatment received)**	Yes	24 (18.9)	74 (54.8)	83 (60.1)	94 (67.1)	<0.001
	No	2 (1.6)	0 (0.0)	2 (1.4)	1 (0.7)	
	Unclear/NR	101 (79.5)	61 (45.2)	53 (38.4)	45 (32.1)	
**Attrition, Follow-up and Protocol Deviation**						
**Report of withdraws and dropouts**	Yes	114 (89.8)	122 (90.4)	127 (92)	120 (85.7)	0.441
	No	6 (4.7)	6 (4.4)	2 (1.4)	6 (4.3)	
	Unclear/NR	7 (5.5)	7 (5.2)	9 (6.5)	14 (10)	
**Withdrawal/dropouts rate acceptable (less than 20%)**	Yes	53 (41.7)	114 (84.4)	116 (84.1)	112 (80)	<0.001
	No	61 (48)	9 (6.7)	13 (9.4)	10 (7.1)	
	Unclear/NR	13 (10.2)	12 (8.9)	9 (6.5)	18 (12.9)	
**Reasons for withdraws/dropouts reported**	Yes	53 (41.7)	102 (75.6)	115 (83.3)	114 (81.4)	<0.001
	No	65 (51.2)	24 (17.8)	11 (8)	9 (6.4)	
	Unclear/NR	9 (7.1)	9 (6.7)	12 (8.7)	17 (12.1)	
**Adverse effects described**	Yes	21 (16.5)	79 (58.5)	85 (61.6)	91 (65)	<0.001
	No	106 (83.5)	56 (41.5)	49 (35.5)	48 (34.3)	
	Unclear/NR	0 (0.0)	0 (0.0)	4 (2.9)	1 (0.7)	
**Short follow-up measurement performed**	Yes	126 (99.2)	130 (96.3)	123 (89.1)	130 (92.9)	0.003
	No	1 (0.8)	5 (3.7)	15 (10.9)	10 (7.1)	
	Unclear/NR	0 (0.0)	0 (0.0)	0 (0.0)	0 (0.0)	
**Long term follow-up measurement performed**	Yes	110 (93.2)	62 (54.9)	63 (58.3)	72 (66.7)	<0.001
	No	8 (6.8)	51 (45.1)	45 (41.7)	36 (33.3)	
	Unclear/NR	0 (0.0)	0 (0.0)	0 (0.0)	0 (0.0)	
**Outcomes**						
**Outcome measures described**	Yes	121 (95.3)	132 (97.8)	138 (100)	137 (97.9)	0.181
	No	2 (1.6)	2 (1.5)	0 (0.0)	2 (1.4)	
	Unclear/NR	4 (3.1)	1 (0.7)	0 (0.0)	1 (0.7)	
**Validity for main outcome measures reported**	Yes	0 (0.0)	6 (4.4)	8 (5.8)	4 (2.9)	0.055
	No	127 (100)	129 (95.6)	130 (94.2)	136 (97.1)	
	Unclear/NR	0 (0.0)	0 (0.0)	0 (0.0)	0 (0.0)	
**Reliability for main outcome measures reported**	Yes	3 (2.4)	13 (9.6)	15 (10.9)	6 (4.3)	0.014
	No	124 (97.6)	122 (90.4)	123 (89.1)	134 (95.7)	
	Unclear/NR	0 (0.0)	0 (0.0)	0 (0.0)	0 (0.0)	
**Responsiveness for main outcome measures reported**	Yes	0 (0.0)	8 (5.9)	4 (2.9)	5 (3.6)	0.103
	No	127 (100)	127 (94.1)	133 (96.4)	135 (96.4)	
	Unclear/NR	0 (0.0)	0 (0.0)	1 (0.7)	0 (0.0)	
**Data Analysis**						
**Descriptive measures identified and reported**	Yes	125 (98.4)	132 (97.8)	137 (99.3)	140 (100)	0.649
	No	1 (0.8)	2 (1.5)	1 (0.7)	0 (0.0)	
	Unclear/NR	1 (0.8)	1 (0.7)	0 (0.0)	0 (0.0)	
**Appropriate statistical analysis used**	Yes	72 (56.7)	122 (90.4)	134 (97.1)	135 (96.4)	<0.001
	No	3 (2.4)	1 (0.7)	0 (0.0)	0 (0.0)	
	Unclear/NR	52 (40.9)	12 (8.9)	4 (2.9)	5 (3.6)	
**Intention to treat analysis used**	Yes	12 (9.4)	48 (35.6)	76 (55.1)	82 (58.6)	<0.001
	No	104 (81.9)	69 (51.1)	50 (36.2)	41 (29.3)	
	Unclear/NR	11 (8.7)	18 (13.3)	12 (8.7)	17 (12.1)	
**Clinical significance reported**	Yes	21 (16.5)	46 (34.1)	56 (40.6)	34 (24.5)	<0.001
	No	106 (83.5)	89 (65.9)	82 (59.4)	104 (74.8)	
	Unclear/NR	0 (0.0)	0 (0.0)	0 (0.0)	1 (0.7)	
**Risk of Random Error**						
**Sample size calculation performed prior to initiation of the study**	Yes	7 (5.5)	13 (9.6)	31 (22.5)	62 (44.3)	<0.001
	No	119 (93.7)	119 (88.1)	105 (76.1)	77 (55)	
	Unclear/NR	1 (0.8)	3 (2.2)	2 (1.4)	1 (0.7)	
**Adequate sample size**	Yes	3 (2.4)	10 (7.4)	28 (20.3)	54 (38.6)	<0.001
	No	3 (2.4)	4 (3)	4 (2.9)	7 (5)	
	Unclear/NR	121 (95.3)	121 (89.6)	106 (76.8)	79 (56.4)	

The results of the logistic regression analyses showed that a significant change over time was evident in 29 out of the 36 quality criteria (that is, 10 risk of bias domains and 26 quality items) of which 26 quality criteria improved over time, while 3 criteria (study described as double blind, blinding of care-provider, and presence of placebo group) worsened over time. Conversely, 8 quality criteria (selective outcome reporting, report of withdraws and dropouts, and 6 blinding-based criteria) did not show a significant change over time (see **Table 5**).

**Table 5:** Results from the logistic regression analysis for low risk of bias or adequate quality criteria^†^.

Criterion^§^^#^		Publication year*		Time-periods of publication year^¶^						
				<1990	1990–1999		2000–2006		2007–2013	
		OR^‡^ (95% CI)	*P*-value		OR (95% CI)	*P*-value	OR (95% CI)	*P*-value	OR (95% CI)	*P*-value
**Risk of Bias Assessment**										
**Sequence generation**		1.080 (1.057–1.103)	<0.001	1.00	1.866 (0.941–3.700)	0.074	5.413 (2.865–10.226)	<0.001	7.905 (4.199–14.883)	<0.001
**Allocation concealment**		1.080 (1.049–1.111)	<0.001	1.00	2.711 (1.026–7.165)	0.044	4.681 (1.857–11.796)	0.001	7.787 (3.168–19.137)	<0.001
**Blinding of participants**		1.015 (1.001–1.030)	0.038	1.00	1.304 (0.774–2.198)	0.317	1.254 (0 .748–2.102)	0.390	1.943 (1.127–3.350)	0.017
**Blinding of outcome assessment**		0.997 (0.984–1.011)	0.768	1.00	0.676 (0.413–1.106)	0.119	1.038 (0.632–1.706)	0.880	1.031 (0.629–1.690)	0.903
**Incomplete outcome data**		1.080 (1.062–1.099)	<0.001	1.00	9.368 (5.107–17.18)	<0.001	14.836 (7.984–27.567)	<0.001	13.660 (7.389–25.253)	<0.001
**Selective outcome reporting**		1.026 (0.995–1.058)	0.100	1.00	1.983 (0.646–6.085)	0.231	5.186 (1.098–24.481)	0.038	2.059 (0.671–6.316)	0.206
**Other sources of bias**		1.091 (1.071–1.111)	<0.001	1.00	7.102 (3.95–12.769)	<0.001	11.056 (6.095–20.056)	<0.001	11.672 (6.431–21.185)	<0.001
	**Baseline comparability**	1.054 (1.037–1.071)	<0.001	1.00	4.815 (2.647–8.759)	<0.001	6.467 (3.409–12.269)	<0.001	4.012 (2.273–7.080)	<0.001
	**Similarity of co-interventions**	1.065 (1.047–1.084)	<0.001	1.00	5.966 (3.267–10.89)	<0.001	7.208 (3.953–13.144)	<0.001	6.411 (3.523–11.668)	<0.001
	**Compliance to treatment**	1.093 (1.073–1.114)	<0.001	1.00	6.054 (3.455–10.608)	<0.001	9.481 (5.351–16.798)	<0.001	12.398 (6.914–22.231)	<0.001
	**Appropriate influence of trial sponsor**	1.043 (1.019–1.067)	<0.001	1.00	1.162 (0.547–2.468)	0.695	1.368 (0.659–2.839)	0.400	3.116 (1.599–6.072)	0.001
**Risk of Random Error**										
**Sample size calculation done prior to study initiation**		1.110 (1.077–1.145)	<0.001	1.00	1.826 (0.704–4.736)	0.215	4.966 (2.100–11.743)	<0.001	13.626 (5.930–31.307)	<0.001
**Adequate sample size**		1.135 (1.093–1.178)	<0.001	1.00	3.306 (0.888–12.303)	0.074	10.521 (3.112–35.566)	<0.001	25.953 (7.858–85.711)	<0.001
**Patient Selection (Inclusion and Exclusion and Description of Participants)**										
**Inclusion criteria clearly defined**		1.066 (1.042–1.091)	<0.001	1.00	2.300 (1.119–4.725)	0.023	11.029 (3.240–37.540)	<0.001	16.911 (3.916–73.028)	<0.001
**Exclusion criteria clearly defined**		1.057 (1.036–1.079)	<0.001	1.00	1.755 (0.913–3.373)	0.092	7.182 (2.671–19.306)	<0.001	9.179 (3.113–27.068)	<0.001
**Assignment, Randomization, and Allocation Concealment**										
**Generation of allocation sequence appropriate**		1.073 (1.052–1.094)	<0.001	1.00	1.881 (0.994–3.557)	0.052	4.390 (2.403–8.018)	<0.001	6.597844 (3.625–12.008)	<0.001
**Generation of allocation sequence concealed**		1.105 (1.063–1.149)	<0.001	1.00	4.032 (1.11–14.642)	0.034	6.599 (1.903–22.881)	0.003	11.272 (3.347–37.964)	<0.001
**Blinding**										
**Study described as double-blind**		0.972 (0.958–0.986)	<0.001	1.00	0.559 (0.339–0.920)	0.022	0.300 (0.176–0.512)	<0.001	0.448 (0.270–0.741)	0.002
**Method of blinding appropriate**		0.991 (0.978–0.004)	0.210	1.00	0.665 (0.408–1.083)	0.102	0.695 (0.428–1.130)	0.143	0.927 (0.570–1.507)	0.762
**Blinding of principal investigator**		1.004 (0.975–1.033)	0.774	1.00	0.297 (0.078–1.126)	0.074	0.806 (0.301–2.159)	0.669	1.342 (0.553–3.255)	0.515
**Blinding of assessor**		0.997 (0.984–1.011)	0.768	1.00	0.676 (0.413–1.106)	0.119	1.038 (0.632–1.706)	0.880	1.031 (0.629–1.690)	0.903
**Blinding of patients**		0.976 (0.962–0.989)	0.001	1.00	0.473 (0.286–0.783)	0.004	0.329 (0.195–0.556)	<0.001	0.564 (0.345–0.922)	0.023
**Blinding of care-providers**		0.952 (0.938–0.967)	<0.001	1.00	0.443 (0.263–0.746)	0.002	0.240 (0.135–0.427)	<0.001	0.223 (0.125–0.400)	<0.001
**Interventions**										
**Presence of a control group**		1.055 (1.039–1.071)	<0.001	1.00	2.526 (1.511–4.223)	<0.001	5.029 (2.834–8.923)	<0.001	4.610 (2.631–8.075)	<0.001
**Presence of a placebo group**		0.936 (0.922–0.951)	<0.001	1.00	0.271 (0.162–0.452)	<0.001	0.162 (0.094–0.276)	<0.001	0.152 (0.089–0.261)	<0.001
**Co-interventions avoided /comparable**		1.065 (1.047–1.084)	<0.001	1.00	5.966 (3.267–10.89)	<0.001	7.208 (3.953–13.144)	<0.001	6.411 (3.523–11.668)	<0.001
**Co-interventions reported for each group**		1.054 (1.027–1.082)	<0.001	1.00	5.333 (1.948–14.59)	0.001	5.485 (2.021–14.888)	0.001	6.058 (2.245–16.347)	<0.001
**Testing of participants compliance to treatment protocol**		1.061 (1.045–1.078)	<0.001	1.00	3.906 (2.339–6.525)	<0.001	4.173 (2.498–6.971)	<0.001	5.438 (3.215–9.197)	<0.001
**Compliance acceptable (80% of treatment received)**		1.079 (1.060–1.098)	<0.001	1.00	5.206 (2.977–9.103)	<0.001	6.476 (3.699–11.337)	<0.001	8.769 (4.973–15.464)	<0.001
**Attrition, Follow-up and Protocol Deviation**										
**Report of withdraws and dropouts**		0.990 (0.968–1.013)	0.412	1.00	1.070 (0.476–2.405)	0.870	1.316 (0.5673–3.055)	0.522	.684 (0.325–1.439)	0.317
**Withdrawal/dropouts rate acceptable (< than 20%)**		1.063 (1.046–1.080)	<0.001	1.00	7.579 (4.226–13.59)	<0.001	7.361 (4.137–13.100)	<0.001	5.584 (3.241–9.621)	<0.001
**Reasons for withdraws/ dropouts reported**		1.065 (1.048–1.082)	<0.001	1.00	4.315 (2.546–7.315)	<0.001	6.981 (3.948–12.343)	<0.001	6.121 (3.521–10.642)	<0.001
**Adverse effects described**		1.068 (1.050–1.086)	<0.001	1.00	7.120 (3.986–12.71)	<0.001	8.095 (4.530–14.463)	<0.001	9.374 (5.233–16.791)	<0.001
**Data Analysis**										
**Appropriate statistical analysis used**		1.122 (1.097–1.149)	<0.001	1.00	7.168 (3.664–14.022)	<0.001	25.590 (8.913–73.466)	<0.001	20.625 (7.903–53.819)	<0.001
**Intention to treat analysis used**		1.088 (1.067–1.111)	<0.001	1.00	5.287 (2.648–10.55)	<0.001	11.747 (5.935–23.249)	<0.001	13.548 (6.842–26.826)	<0.001
**Clinical significance reported**		1.022 (1.006–1.038)	0.007	1.00	2.608 (1.448–4.697)	0.001	3.447 (1.933–6.147)	<0.001	1.634 (0.890–2.999)	0.113

**Abbreviations:** CI, confidence interval; OR, odds ratio.

^**†**^ **Low** risk vs. others (both **Unclear** and **High** risk of bias); or **Yes** vs. others (both **No** and **Unclear/Not-reported)**.

^*^ Time was entered in the logistic regression model as a continuous variable.

^**¶**^ Time was entered in the logistic regression model as a categorical variable.

^**§**^ The following criteria were not considered in the analysis because of either having a small number of trials judged as being adequate: ***overall risk of bias; blinding of data analyst; validity*, *reliability*, *and responsiveness for main outcome measures reported; study described as randomized; and early stopping of trial*.**

^**#**^ The following criteria were not considered in the analysis because of having a small number of trials judged as being inadequate/unclear: **treatment protocol adequately described for treatment, control, or comparison group; short or long follow-up measurement performed; outcome measures described; descriptive measures identified and reported.**

^**‡**^ The factor which the odds of the quality criteria, being adequate, increased by every year.

## Discussion

Bias is a threat to the quality of trials [38, 39]; it may impact the reported treatment effect estimates in randomized clinical trials [40–44]. The degree of bias in randomized clinical trials of oral health intervention has decreased over time according to our study. We used the Cochrane Collaboration’s risk of bias tool, in addition to a comprehensive set of reporting and methodological characteristics (selected from seven quality assessment tools reported to be valid), to assess the methodological quality of randomized clinical trials of oral health intervention. Thus, this study provides an in-depth analysis of the methodological characteristics and risks of bias present in dental literature from 1955–2013.

In the majority of the quality items and risk of bias domains, our study showed that the proportion of trials having inadequate quality (or having high or unclear risk of bias) decreased significantly over time. This encouraging trend is similar to what was identified in a recently published report by Reveiz et al. [19]. However, rather than conducting standardized data extraction from each trial, Reveiz used a risks of bias assessment reported by the investigators of reviews; this might be problematic, especially given the documented low reliability of the Cochrane Collaboration’s risk of bias tool [26, 45]. The trend in our study is comparable to that found in a cohort of child related trials [46] and medical randomized clinical trials [5]. A similar trend was identified when the methodological quality of trials of physical therapy interventions was assessed, where an improvement of nearly 0.6 points each decade was found in the total Physiotherapy Evidence Database (PEDro) score [47]. The trend in our report is also similar to a recently published study [48] which analyzed 20,920 randomized clinical trials included in Cochrane reviews; the study concluded that the proportion of trials at unclear risk of bias decreased over time, especially for sequence generation (fell from 69.1% to 31.2%) and for allocation concealment (fell from 70.1% to 44.6%).

Although an improvement over time was identified in our study with respect to randomized clinical trials of oral health intervention, results of risks of bias, risks of random error, and reported methodological quality assessments were still unpropitious, indicating substandard quality and a high potential for bias. We believe that sizable improvements in the conduct and reporting of oral health randomized clinical trials is possible. The fact that the proportion of trials having low risk of bias did not exceed 60% in the majority of risk of bias domains is a significant concern. Because inadequate design and unrigorous conduct of a trial can bias the estimation of the treatment effect size, decisions made in dental practice might not be based on valid findings. For example, allocation concealment (a process of concealing information about which patients are to be assigned to a new treatment versus those to be given a conventional therapy) and sequence randomization (allocation is carried out using a chance mechanism so that neither the participant nor the investigator will know in advance which will be assigned to an intervention) [49–51] were unclear in 84.8% and 66.7% of the trials, respectively, although these factors improved significantly over time. It should be noted that an “unclear” risk of bias results in a trial that may not mirror the actual design and conduct of the trial. Because journals have a word limit that may restrict authors in reporting detailed methods, all of the methodological characteristics used might not be reported [52], thus restricting the accuracy of quality assessment tools. In the field of dentistry, treatment effect size estimates were found to be larger in trials with inadequate trial’s design characteristics which can can affect overestimation of beneficial or underestimation of harmful treatment effects within trials [15, 44].

Our study showed that sample size calculation was not performed or reported in 79.1% of the trials; this finding is concerning as it raises the risks of random errors leading to the risk of having increased numbers of false positive conclusions (type I errors) and false negative conclusions (type II errors) [53] in randomized clinical trials of oral health interventions. It has recently been estimated that randomized clinical trials have to include at least 1000 participants in order for simple randomisation to serve its purpose: allocation a sufficient sample to allow a fair comparison of intervention effects [54].

Moreover, having an appropriate influence of trial sponsor was found to be inadequate or not reported in 83.3% of the trials. This is concerning because sponsorship bias in oral heath randomized clinical trials can potentially benefit the sponsoring company and might lead to inappropriate clinical decisions. Industry sponsorship of trials may lead to favorable findings than sponsorship by other sources [43]. For example, a recently published report examined the influence of industry sponsorship in 41 randomized clinical trials of dental implants and found that the probability of implant failure in sponsored randomized clinical trials was much lower than the probability of implant failure in nonsponsored randomized clinical trials. Also, there is a debate whether the Cochrane risk of bias tool should include funding source as an individual item or not [55–57]. Accordingly, there is a need for large meta-epidemiological studies to quantify the extent of bias associated with influence of funders on the magnitude of treatment effect size in oral heath randomized clinical trials.

Our study revealed that more than half of the trials were published in specialist journals, and that nearly half of the trials were from the United Kingdom and the United States. These trends are similar for medical trials [52]. Possibly the interest of government and public sectors in the aforementioned countries is responsible for facilitating the financial support for such randomized clinical trials [58].

The improvement observed in risks of bias and reported methodological quality of randomized clinical trials over time, could be attributed to efforts made by editors and reviewers of oral health journals, through endorsement of the CONSORT Statement [59, 60], and by the mandatory implementation of trial registration, as recommended by the ICMJE [6, 61].

The CONSORT Statement is an accepted and widely used approach in medical and dental research to assess the reporting quality of randomized clinical trials. This approach covers the fundamental aspects of a trial’s reporting quality; the CONSORT Statement aims to advance the transparency and quality of medical and dental trial reporting through the creation of reporting criteria [62, 63]. It has been endorsed during the last 10 years by several medical journals worldwide, including the majority of high impact oral health journals [59, 63]. Although the CONSORT Statement applies only to reporting quality, it is used commonly and erroneously by many dentistry researchers as a methodological quality assessment tool. Moreover, the SPIRIT Statement has recently become a widely used tool that aims to advance and improve the quality of protocols of randomized clinical trials and involve a set of criteria to address in a trial protocol [64, 65].

In the dental literature, the concepts “reporting quality” and “methodological quality” are often used interchangeably, contributing to conceptual ambiguity. Methodological quality depends mainly on the degree to which the design, conduct, and analysis of a trial follows the highest possible standards (to reduce multiple potential biases) and, hence, suggests that the findings can be based on the used intervention [1, 16, 17]. While the internal validity of a trial (which is closely connected to the risks of bias [16] and the methodological quality) should be the core of quality assessment, “reporting quality” is mistakenly used by researchers as an alternative for methodological quality; this has induced a conceptual ambiguity in the definition of trial “quality” [28]. In the context of medical research, a risk of bias assessment will benefit from an explicit and unambiguous definition of “methodological quality”.

Although endorsement of the CONSORT Statement by dental journal editors and reviewers results in improvement in the reporting quality of trials, it does not guarantee compliance by trialists [60]. Furthermore, reliance on the CONSORT Statement only, may give reviewers, authors, and readers a false sense of security. Transparent reporting is desirable, but it does not necessarily raise methodological quality or lower the risks of bias [39]. For example, good reporting fails to prevent publication bias (i.e., trials of methods that have beneficial and large effects are published rapidly in journals with high impact), and selective outcome reporting (i.e., beneficial findings get publishing priority) [16]. These reported biases can exaggerate the magnitude of treatment effects in clinical trials, and can distort findings in meta-analysis [66, 67]. Implementation of the mandatory trial registration policy [4] could contribute to the improvement of trial quality and lower the risks of bias identified in this study. Implementation of the mandatory trial registration policy started over 10 years ago by 11 leading medical journals, and is currently applied by over 300 medical journals [6], including many leading dental journals [68]. However, recently only 23% of dental randomized clinical trials, published in 15 high impact dental journals, were registered [61, 69].

The results of this study have several implications. Dental trialists need to explicitly report their trials’ results and adhere to published guidelines. Dental journal editors and reviewers should continue to be committed to international initiatives and statements developed to ensure adequate and appropriate conduct and reporting of randomized clinical trials. Adherence to the above guidelines can reduce the risk that inaccurate conclusions will be drawn from the research and, accordingly, will reduce inappropriate recommendations regarding treatment interventions in dental practice. To adequately apply trial findings to care of their patients, clinicians should be aware of the design, conduct, and reporting of a clinical trial. This knowledge will enable a clinician to deliver the best possible results in his or her own dental practice.

Our findings call for oral health policy makers, methodologists, clinicians, and researchers to develop initiatives for improving clinical trials, which would spread such actions within the oral health community. The formation of a global oral health initiative that aims to improve the conduct and reporting of oral health trials, and that prioritizes methodological criteria in oral health research, would be an example of a potentially needed measure to raise standards of randomized clinical trials.

### Strengths and limitations

This cross-sectional observational study provided a comprehensive assessment of oral health randomized clinical trials with respect to trial characteristics, reporting quality, methodological characteristics, and risks of bias, and attempts to identify the variation of these factors over time. The range and size of our sample provided a comprehensive evaluation of oral health trials over the 58 year-period of 1955–2013. One of the strengths of our research was the data extraction method, which was performed in duplicate by two independent assessors to ensure high accuracy and avoid potential biases during the data extraction process. We performed a standardized data extraction rather than relying on the risks of bias assessment reported in systematic reviews, which was the case in recent reports by Reveiz et al. where the risks of bias in medical randomized clinical trials were assessed (19) and by Dechartres et al. using data from 20,920 randomized clinical trials included in Cochrane reviews within all disease areas [48].

A potential limitation of our research is that the choice of sample trials is not strictly random. Our sample of trials originated from 64 dental, oral, and craniofacial meta-analyses and was designed to cover the overall spectrum of clinical oral health research during 1955–2013, therefore, we believe that it represents a realistic cohort for that period. However, as the topics of meta-analyses are often focused on topics of high importance to researches and societies, we cannot exclude that the quality of the trials may be better than the average dentistry randomized clinical trial. Also, it is a drawback that we did not publish our protocol for this observational study.

Another potential limitation is that we did not contact the authors of the studied trials for missing data. A large proportion of the trials were published before the year 2000 when an author’s correspondence information was sometimes not current and not always provided in the publication. Moreover, because we extracted data based on the data reported in the published trials, the actual risk of bias potential was not visible in the majority of risk of bias domains studied due to the poor quality of the reporting identified in the studied trials. As our study did not look at factors that contributed to methodological quality improvement over time, these factors must be considered in future research.

We applied an educated judgement to assign each trial to a primary dental specialty (e.g., dental public health), although the trial could be classified under more than one specialty (e.g., both pediatric dentistry and dental public health).

## Conclusions

Our study showed a significant increase over time (1955–2013) in the proportion of trials judged to be adequate in risks of bias, risks of random errors, reporting quality, and methodological quality. However, the proportion of trials judged as having low risk of bias did not exceed 60% in the majority of the risk of bias domains. We found the risks of bias and the quality assessment in the studied trials to be generally unfavorable. That is, in the trials of oral health interventions the methodology and reporting quality were substandard, resulting in a high potential for bias. We believe that a commitment to international initiatives by researchers, journal editors, and manuscript reviewers can contribute to the development of more stringent methodology and more detailed reporting of randomized clinical trials of oral health interventions.

## Supporting information

S1 Appendix Tools and items to assess quality of randomized trials. (DOCX)

S2 Appendix Guidelines for the quality assessment of trials based on the tools most commonly used in health research. (DOCX)

S3 Appendix The Cochrane Collaboration’s tool for assessing risk of bias. (DOCX)

S4 Appendix Guidelines for evaluating the risk of bias of trials. (DOCX)

S5 Appendix The list of included trials. (DOCX)

S6 Appendix PRISMA 2009 checklist. (DOC)
