# Supplementary material for: Randomized clinical trials in dentistry: Risks of bias, risks of random errors, reporting quality, and methodologic quality over the years 1955–2013
Source: PLoS One. 2017 Dec 22;12(12):e0190089. doi: 10.1371/journal.pone.0190089 (PMC5741237; doi:10.1371/journal.pone.0190089)
Supplement: S1 Appendix — (DOCX) [file pone.0190089.s001.docx]

| **Appendix S1. Tools and items to assess quality of randomized trials [**[**1-9**](#_ENREF_1)**]** | | | | | | | | | | | | |
| --- | --- | --- | --- | --- | --- | --- | --- | --- | --- | --- | --- | --- |
| **Items included in the scales** | **Jadad [**[**2**](#_ENREF_2)**]** | **MAT*** **[**[**3**](#_ENREF_3)**]** | **Delphi [**[**4**](#_ENREF_4)**]** | **Van Tulder**  **[**[**5**](#_ENREF_5)**]** | **MAT-AM**£ **[**[**6**](#_ENREF_6)**]** | **PeDro [**[**7**](#_ENREF_7)**,**[**8**](#_ENREF_8)**]** | **Bizzini [**[**9**](#_ENREF_9)**]** | **Total items (n)** | **FREQ %** | **R**† | **C**‡ | **BIAS** |
| **PATIENT SELECTION (INCLUSION AND EXCLUSION AND DESCRIPTION OF SUBJECTS)** | | | | | | | | | | | | |
| **Inclusion criteria clearly defined/eligibility criteria specified** |  | X | X |  | X | X | X | 5 | 71.4 | X |  | Selection Bias |
| **Exclusion criteria defined** |  | X |  |  |  |  | X | 2 | 28.6 | X |  | Selection Bias |
| **Baseline comparability (group equivalence, homogeneity) regarding the most important prognostic indicators** |  | X | X | X | X | X | X | 6 | 85.7 |  | X | Selection Bias |
| **ASSIGNMENT, RANDOMIZATION, AND ALLOCATION CONCEALMENT** | | | | | | | | | | | | |
| **Study described as randomized** | X |  |  |  |  | X | X | 3 | 42.9 | X |  | Selection Bias |
| **Method of randomization described and appropriate** | X | X |  | X | X | X |  | 5 | 71.4 |  | X | Selection Bias |
| **Method of randomization concealed** |  | X | X | X | X | X |  | 5 | 71.4 |  | X | Selection Bias |
| **BLINDING** | | | | | | | | | | | | |
| **Study described as double blind** | X |  |  |  |  |  |  | 1 | 14.3 | X |  | Performance Bias/Detection Bias (outcome assessment) |
| **Method of blinding described** | X |  |  |  |  |  |  | 1 | 14.3 | X |  | Performance Bias/Detection Bias (outcome assessment) |
| **Blinding of investigator** |  | X | X | X | X | X | X | 6 | 85.7 |  | X | Detection Bias (outcome assessment) |
| **Observer blinding evaluated and successful** |  | X |  |  |  |  |  | 1 | 14.3 |  | X | Detection Bias  (outcome assessment) |
| **Blinding of subjects/patients** |  | X | X | X | X | X |  | 5 | 71.4 |  | X | Performance Bias/Detection Bias  (self-reported outcome assessment) |
| **Blinding of therapist/care provider** |  | X | X | X | X | X |  | 5 | 71.4 |  | X | Performance Bias |
| **Blinding of the outcome (data analyst)** |  | X |  |  |  |  |  | 1 | 14.3 |  | X | Detection Bias |
| **INTERVENTIONS** | | | | | | | | | | | | |
| **Treatment protocol adequately described for the treatment group regarding type of intervention, duration of each intervention, frequency, intensity, and dosage** |  | X |  |  | X |  | X | 3 | 42.9 | X |  | Performance Bias |
| **Treatment protocol adequately described for the control group regarding type of intervention, duration of each intervention, frequency, intensity, and dosage** |  | X |  |  | X |  |  | 2 | 28.6 | X |  | Performance Bias |
| **Treatment protocol adequately described for the comparison group regarding type of intervention, duration of each intervention, frequency, intensity (if applicable) *** |  | X |  |  | X |  |  | 2 | 28.6 | X |  | Performance Bias |
| **Control adequate (presence of a control group)** |  |  |  |  |  |  | X | 1 | 14.3 |  | X | Performance Bias |
| **Placebo adequate (presence of a placebo group)** |  |  |  |  |  |  | X | 1 | 14.3 |  | X | Performance Bias |
| **Cointerventions avoided/or comparable** |  | X |  | X | X |  | X | 4 | 57.1 |  | X | Performance Bias |
| **Cointerventions reported for each group separately** |  |  |  |  | X |  |  | 1 | 14.3 | X |  | Performance Bias |
| **Testing of subject compliance to treatment protocol (report of compliance)** |  | X |  |  |  |  |  | 1 | 14.3 | X | X | Performance Bias/Compliance bias |
| **Compliance acceptable in all groups (80% of treatment received)** |  |  |  | X | X |  |  | 2 | 28.6 |  | X | Performance Bias/Compliance bias |
| **ATTRITION, FOLLOW UP, AND PROTOCOL DEVIATION** | | | | | | | | | | | | |
| **Report of withdraws and dropouts (rate)** | X | X |  | X | X |  | X | 5 | 71.4 | X |  | Attrition Bias |
| **Withdrawal/dropouts rate acceptable (less than 20%)** |  | X (< 5%) |  | X | X | X (15%) |  | 4 | 57.1 |  | X | Attrition Bias |
| **Reasons for withdraws and dropouts reported** | X | X |  |  |  |  | X | 3 | 42.9 | X |  | Attrition Bias |
| **Adverse effects described** |  | X |  |  | X |  |  | 2 | 28.6 | X |  | Reporting Bias |
| **Short follow-up measurement performed** |  |  |  |  | X |  |  | 1 | 14.3 |  | X | Attrition Bias |
| **Long term follow-up measurement performed** |  |  |  |  | X |  |  | 1 | 14.3 |  | X | Attrition Bias |
| **OUTCOMES** | | | | | | | | | | | | |
| **Outcome measures described** |  |  |  |  |  |  | X | 1 | 14.3 | X |  | Reporting Bias |
| **Validity for main outcome measures reported** |  |  |  |  |  |  | X | 1 | 14.3 | X | X | Information Bias |
| **Responsiveness for main outcome measures reported** |  |  |  |  |  |  | X | 1 | 14.3 | X | X | Information Bias |
| **Reliability for main outcome measures reported** |  |  |  |  |  |  | X | 1 | 14.3 | X | X | Information Bias |
| **STATISTICAL ANALYSIS** | | | | | | | | | | | | |
| **Descriptive measures (point estimates and measures of variability) identified and reported for the primary outcome** |  | X | X |  | X | X | X | 5 | 71.4 | X |  | Reporting Bias |
| **Appropriate statistical analysis used** |  | X |  |  |  | X | X | 3 | 42.9 |  | X | Statistical Bias |
| **Sample size described for each group** |  |  |  |  | X |  |  | 1 | 14.3 | X |  | Threats to precision |
| **Intention to treat analysis used** |  | X | X | X | X | X | X | 6 | 85.7 |  | X | Selection bias/attrition bias |
| **RISK OF RANDOM ERROR** | | | | | | | | | | | | |
| **Sample size calculation performed prior to initiation of the study** |  |  | X |  |  |  |  |  | 14.3 |  | X | Threats to precision |
| **Adequate sample size** |  | X |  |  |  |  | X | 2 | 28.6 |  | X | Threats to precision |
| * Maastricht  £ Maastricht Amsterdam  † Reporting  ‡ Conducting | | | | | | | | | | | | |

**References for Appendix S1**

1. Armijo Olivo S, Fuentes CJ, Ospina M, Saltaji H, Hartling L (2013) Inconsistency in the Items Included in Tools Used in General Health Research and Physical Therapy to Evaluate the Methodological Quality of Randomized Controlled Trials: A Descriptive Analysis. BMC Medical Research Methodology 13 (116):1-19

2. Jadad AR, Moore RA, Carroll D, Jenkinson C, Reynolds DJ, Gavaghan DJ, McQuay HJ (1996) Assessing the quality of reports of randomized clinical trials: is blinding necessary? Controlled clinical trials 17 (1):1-12

3. De Vet HCW, De Bie RA, Van Der Heijden GJMG, Verhagen AP, Sijpkes P, Knipschild PG (1997) Systematic reviews on the basis of methodological criteria. Physiotherapy 83 (6):284-289

4. Verhagen AP, de Vet HC, de Bie RA, Kessels AG, Boers M, Bouter LM, Knipschild PG (1998) The Delphi list: a criteria list for quality assessment of randomized clinical trials for conducting systematic reviews developed by Delphi consensus. Journal of Clinical Epidemiology 51 (12):1235-1241

5. Van Tulder M, Furlan A, Bombardier C, Bouter L (2003) Updated method guidelines for systematic reviews in the Cochrane Collaboration Back Review Group. Spine 28 (12):1290-1299

6. Van Tulder MW, Assendelft WJJ, Koes BW, Bouter LM, Bombardier C, Nachemson AL, Esmail R, Deyo RA, Shekelle PG, Bouter LM, De Bie RA, Waddell G, Roland M, Guillemin F (1997) Method guidelines for systematic reviews in the Cochrane Collaboration Back Review Group for spinal disorders. Spine 22 (20):2323-2330

7. Moseley AM, Herbert RD, Sherrington C, Maher CG (2002) Evidence for physiotherapy practice: a survey of the Physiotherapy Evidence Database (PEDro). Australian Journal of Physiotherapy 48 (1):43-49

8. Sherrington C, Herbert RD, Maher CG, Moseley AM (2000) PEDro. A database of randomized trials and systematic reviews in physiotherapy. Manual therapy 5 (4):223-226

9. Bizzini M, Childs JD, Piva SR (2003) Systematic review of the quality of randomized controlled trials for patellofemoral pain syndrome. Journal of Orthopaedic & Sports Physical Therapy 33 (1):4-20
