# Supplementary material for: Randomized clinical trials in dentistry: Risks of bias, risks of random errors, reporting quality, and methodologic quality over the years 1955–2013
Source: PLoS One. 2017 Dec 22;12(12):e0190089. doi: 10.1371/journal.pone.0190089 (PMC5741237; doi:10.1371/journal.pone.0190089)
Supplement: S2 Appendix — (DOCX) [file pone.0190089.s002.docx]

| **Appendix S2. Guidelines for the quality assessment of trials based on the tools most commonly used in health research [**[**1-9**](#_ENREF_1)**]** | | | | |
| --- | --- | --- | --- | --- |
| **Item No.** | **Items/Definitions** | **Yes (High Quality)** | **No (Low Quality)** | **Unclear Quality** |
| **1** | **Inclusion, eligibility criteria for participants**  (e.g., pathology of interest, age, gender, and special characteristics) | The authors describe inclusion criteria of the study participants. They clearly show the characteristics of the study population. | The authors do not describe the inclusion criteria of the study participants. | There is insufficient information about inclusion and exclusion criteria to permit a judgment. |
| **2** | **Exclusion, eligibility criteria for participants**  (e.g., pathology of interest, age, gender, and special characteristics) | The authors describe exclusion criteria of the study participants. There is clear information regarding the population under study. | The authors do not describe the exclusion criteria of the study participants. | There is insufficient information about inclusion and exclusion criteria to permit a judgment. |
| **3** | **Baseline (group equivalence of participants, homogeneity) regarding the most important prognostic indicators** (the groups are similar at the start of the trial). | The authors state that the groups were comparable or had an equal prognostic factor baseline. They analyzed this by comparing groups through a statistical test in all variables of interest. Or the authors state that groups are not comparable, and they adjusted statistically (e.g., by using ANCOVA).  Groups must be comparable with regard to (for example) pain, global perceived effect, participation in daily activities; at least one of the main outcomes must be described, age; sex; and pre-existing participation problems. | The authors state that groups are not equal at baseline and they did not adjust for any difference. | There is insufficient information to permit a judgment. |
| **4** | **Study is described as randomized** | The authors use the word randomized, randomization, random, or minimization as derived within the title, abstract, or text. | The word randomized or randomization or any similar word does not appear in the title, abstract, or text. | There is insufficient information to permit a judgment. |
| **5** | **Randomization process performed** | The authors use the word randomized or randomization or a similar word as derived within the title, abstract, or text, to describe the method performed in the trial, such as random number tables, computer program, etc. | The authors do not describe the method performed in the trial, such as random number tables, computer program. | There is insufficient information about the sequence generation process to permit a judgment. |
| **6** | **Method of randomization described and appropriate** | The authors use the word randomized or randomization or a similar word as derived within the title, abstract, or text and described the method used for doing the randomization such as random number tables, computer program, etc. The investigators describe a random component in the sequence generation process such as:  • Referring to a random number table; • Using a computer random number generator; • Minimization;  • Coin tossing; • Shuffling cards or envelopes; • Throwing dice; • Drawing of lots; | The authors do not describe the method used for doing the randomization, such as random number tables, computer program. Other nonrandom methods were used such as:   - hospital records numbers, - time of presentation, - alternate numbers, - date of birth | There is insufficient information about the sequence generation process to permit a judgment. |
| **7** | **Method of randomization concealed** | Allocation was done and is appropriate  Assignment is generated by an independent person not responsible for determining the eligibility of the patients: To score yes this person: must have no information about patients included in the trial; and must have no influence on the assignment sequence or decision about the eligibility of the patients.  Participants and investigators enrolling participants could not foresee assignment because one of the following, or an equivalent method, is used to conceal allocation:  • Central allocation (including telephone, web-based, and pharmacy-controlled randomization); • Sequentially numbered drug containers of identical appearance; provided by a different person who did the randomization allocation; • Sequentially numbered, opaque, sealed envelopes. | Participants or investigators enrolling participants could possibly foresee assignments and thus introduce selection bias. | There is insufficient information to permit a judgment. |
| **8** | **Study described as double blind** | “Double blind” is the description in the study related to “blindness.”  Also, it should be stated that neither the person doing the assessments, nor the study participants could identify the intervention being assessed. | Not described as double blind. | There is insufficient information to permit a judgment. |
| **9** | **The method of blinding was appropriate** | The authors use the blinding method appropriately.  Blinding of participants/patients is a “must” when outcomes are subjective or self-reported.  When outcomes are measured by an assessor, the assessors should be blinded to group allocation.  Also, score “completely done” when it is unlikely that the blinding could have been broken and the nonblinding of others is unlikely to introduce bias. No blinding, but the review authors judge that the outcome and the outcome measurement are not likely to be influenced by lack of blinding.  Objectives automatized outcomes coming from databases or hospital register office. | There is no blinding or incomplete blinding is performed, and the outcome or outcome measurement is likely to be influenced by lack of blinding. | There is insufficient information to permit a judgment. |
| **10** | **Blinded investigator** | The study describes in the title, abstract, or text that the investigator was blinded. The blinding was appropriate. | The study describes in the title, abstract, or text that the investigator was not blinded. | There is insufficient information to permit a judgment. |
| **11** | **Blinded assessor** | The study describes in the title, abstract, or text that the assessor was blinded. The blinding was appropriate. When outcomes are measured by an assessor, the assessors should be blinded to group allocation. | The study describes in the title, abstract, or text that the assessor was not blinded. | There is insufficient information to permit a judgment. |
| **12** | **Blinded subjects/patients** | The study describes in the title, abstract, or text that subjects/patients were blinded. The blinding was appropriate. | The study describes in the title, abstract, or text that subjects/patients were not blinded. | There is insufficient information to permit a judgment. |
| **13** | **Blinded therapist/care-provider** | The study describes in the title, abstract, or text that the therapists/care-providers were blinded. The blinding was appropriate. | The study describes in the title, abstract, or text that the therapists/care-providers were not blinded, or because of the nature of the intervention (e.g., exercise prescription or supervision, etc.), the therapist could not be blinded. | There is insufficient information to permit a judgment. |
| **14** | **Blinded statistician** | The study describes in the title, abstract, or text that the statistician was blinded. The blinding was appropriate. | The study describes in the title, abstract, or text that the statistician was not blinded. | There is insufficient information to permit a judgment. |
| **15** | **Treatment protocol for experimental group** | The authors describe doses, frequency, intensity, of the treatment protocol for the experimental group (repetition, days per week, length of time) in enough detail to reproduce the intervention. At least three of the five points below are described for the experimental intervention;  1. type of intervention;  2. intensity of the intervention;  3. duration and site of each treatment session;  4. frequency of treatment sessions; and  5. total number of treatment sessions. | The authors do not describe the treatment protocol. | The authors do not describe enough aspects of the treatment protocol for the experimental group that would allow reproducibility of the intervention. |
| **16** | **Treatment protocol for control group** | The authors describe doses, frequency, intensity, position of treatment protocol for the comparison group (repetition, days per week, length of time) in enough detail to reproduce the intervention.  At least three of the five points below are described for the control intervention; if more than two types of intervention are compared, take only two of them):  1. type of intervention;  2. intensity of the intervention;  3. duration and site of each treatment session;  4. frequency of treatment sessions; and  5. total number of treatment sessions. | The authors do not describe the treatment protocol. | The authors do not describe enough aspects of the treatment protocol for the control group that would allow reproducibility of the intervention. |
| **17** | **Treatment protocol for the control or comparison group #2** (if applicable) | The authors describe doses, frequency, intensity, position, of treatment protocol for the comparison group (repetition, days per week, length of time) in enough detail to reproduce the intervention.  At least three of five are described for second control intervention; if more than two types of interventions.  1. type of intervention;  2. intensity of the intervention;  3. duration and site of each treatment session;  4. frequently of treatment sessions; and  5. total number of treatment sessions. | The authors do not describe the treatment protocol. | The authors do not describe enough aspects of treatment protocol for control group #2 that would allow reproducibility of the intervention. |
| **18** | **Control group** | The study employs a control group (i.e., no-treatment/waiting list/standard care). | A control group was not used. | There is insufficient information to permit a judgment. |
| **19** | **Placebo** | The authors describe the use of a placebo group. The authors used a credible sham and there is certainty that this sham was good and was not discovered by the patients. | A placebo group was not used. | There is insufficient information to permit a judgment. |
| **20** | **Cointerventions avoided or comparable.**  Cointerventions are interventions other than the treatment under study. | The authors state that subjects did not receive an additional intervention, or that cointerventions were balanced between treatment and control groups. Data about cointerventions are presented and comparable between treatment and control groups. | Subjects received additional interventions besides the intervention under study. The cointerventions were not balanced between treatment and control groups. | There is insufficient information to permit a judgment.  **N/A:** Treatment and control groups did not receive an intervention in addition to the intervention under study. |
| **21** | **Cointerventions reported for treatment and control groups.** | The authors describe cointerventions for treatment and control groups separately (type of intervention, frequency, dosage, etc.). | The authors do not explain the type or process of cointervention. | There is insufficient information to permit a judgment. |
| **22** | **Subject compliance to treatment protocol.**  *Compliance* means that the subjects follow the treatment as planned; that is, the subjects attend at least 80% of the treatment sessions. | The authors describe that they registered the compliance of the subjects (e.g., through logs or diaries), or they say that subjects were compliant with the treatment because they attended at least 80% of the treatment sessions. Compliance monitoring is assumed for a one-time intervention. | The authors did not test subject compliance to treatment protocol. | There is insufficient information to permit a judgment. |
| **23** | **Acceptable compliance** | There is ≥ 80% compliance in treatment and control groups. The control group might have to be “compliant” as well. For example, in an exercise intervention, the control group would have to comply by doing no exercise. | There is less than 80% compliance in treatment and control groups. | There is insufficient information to permit a judgment. |
| **24** | **Report of withdrawals and dropouts** | There is clear reporting of all withdrawals and dropouts. Generally, this is done by using a flowchart.   1. Number of dropouts. 2. If there were no withdrawals, this fact should be stated in article. | Withdrawals and dropouts are not reported. |  |
| **25** | **Acceptable withdrawal/dropout rate** | The withdrawal/dropout rate in the study was less than or equal to 20%.  Or with multiple time points, at any point there must be at least 85% patients included in analysis | The withdrawal/dropout rate was > 20% when only one-time point was evaluated. |  |
| **26** | **Reasons for dropouts** | There is clear reporting of all dropouts and the reason for each dropout is given. | Reasons for dropouts are not reported. |  |
| **27** | **Adverse effects of the intervention** | Adverse effects of the intervention are reported. | Adverse effects of the intervention are not reported. |  |
| **28** | **Short follow-up measurement of the intervention** | An outcome assessment of the intervention was performed at the end the of intervention period. | The outcome was measured before the treatment was finished and there was no outcome evaluation after the treatment was completed. |  |
| **29** | **Long term follow-up of the intervention** | An outcome assessment was performed three or more months after the treatment was completed. | The outcome was assessed less than three months after the treatment was completed. | **N/A:** There is no assessment performed in the study. |
| **30** | **Description of outcome measures of the intervention** | The authors describe all the treatment outcomes, primary and secondary, and they explain how to score them. | The authors do not describe the outcome(s) of the treatment. | There is insufficient information to permit a judgment. |
| **31** | **Validity of the main outcome** | The authors report the validity of the measure of the main outcome of the intervention (this can be done by references). | The authors do not report the validity of the main outcome measure of the intervention. | There is insufficient information to permit a judgment. |
| **32** | **Responsiveness of the main outcome** | Authors report the responsiveness of the main outcome. This can be done by references. Responsiveness means sensitive to change, or able to detect change. | The authors do not report the responsiveness of the main outcome. | There is insufficient information to permit a judgment. |
| **33** | **Reliability of the main outcome** | The authors report the reliability of the main outcome of the intervention. | The authors do not report the reliability of the main outcome of the intervention. | There is insufficient information to permit a judgment. |
| **34** | **Descriptive measures (point estimates and measures of variability) reported for the primary outcome of the treatment.**  Point estimates include means, medians, modes, and measures of variability and include standard deviation, 95% confidence interval, and quartile. | The authors describe both point estimates (e.g. mean) and variability measures (e.g. SD or CI “confidence interval”) for the main outcome of the intervention. | The authors do not describe either the point estimates or the measures of variability for the main outcome of the intervention. |  |
| **35** | **Appropriate statistical analysis** | The authors describe the analysis for each outcome and the alpha level chosen, and it seems that the chosen analysis was a good approach to the research question.  Statistical comparisons and variability between or among groups in the trial must be provided. Authors need to provide estimates and variability data. | The statistical analysis is not appropriate. | There is insufficient information to permit a judgment. |
| **36** | **Sample size described for each group** | The sample size is described for each group in the study. | The sample size is not described for each group in the study. |  |
| **37** | **Intention to treat (ITT) analysis used (patients are analyzed in the groups to which they were randomized)**  All randomized patients have to be analyzed for the most important outcome measures and at the most important moments of intervention effect measurement or of whether there are no withdrawals or loss to follow-up measurements of intervention. | The authors used an intention to treat (ITT) analysis principle and, according to their evaluation, they analyzed the subjects as randomized. There are no missing data so it is assumed that the ITT principle was followed if no other protocol deviations occurred. | The authors did not use the ITT principle or the authors said that they used ITT but an evaluation of the study indicates that subject analysis was not randomized. | There is insufficient information to permit a judgment. |
| **38** | **Sample size calculation performed prior to initiation of the study** | The authors describe a sample size calculation prior to start the study and calculate how many participants need to be recruited for the study to have an acceptable power. | The authors did not perform a sample size calculation prior to the start of the study. |  |
| **39** | **Adequate sample size** | The sample size calculated is the same as the sample size recruited and maintained throughout the trial. | The sample size calculated is not the same as the sample size recruited, or the dropout rate is more than 20%, or the sample size was insufficient to show a significant treatment effect (acceptable power). | There is insufficient information to permit a judgment. |

**References for Appendix S2**

1. Armijo Olivo S, Fuentes CJ, Ospina M, Saltaji H, Hartling L (2013) Inconsistency in the Items Included in Tools Used in General Health Research and Physical Therapy to Evaluate the Methodological Quality of Randomized Controlled Trials: A Descriptive Analysis. BMC Medical Research Methodology 13 (116):1-19

2. Jadad AR, Moore RA, Carroll D, Jenkinson C, Reynolds DJ, Gavaghan DJ, McQuay HJ (1996) Assessing the quality of reports of randomized clinical trials: is blinding necessary? Controlled clinical trials 17 (1):1-12

3. De Vet HCW, De Bie RA, Van Der Heijden GJMG, Verhagen AP, Sijpkes P, Knipschild PG (1997) Systematic reviews on the basis of methodological criteria. Physiotherapy 83 (6):284-289

4. Verhagen AP, de Vet HC, de Bie RA, Kessels AG, Boers M, Bouter LM, Knipschild PG (1998) The Delphi list: a criteria list for quality assessment of randomized clinical trials for conducting systematic reviews developed by Delphi consensus. Journal of Clinical Epidemiology 51 (12):1235-1241

5. Van Tulder M, Furlan A, Bombardier C, Bouter L (2003) Updated method guidelines for systematic reviews in the Cochrane Collaboration Back Review Group. Spine 28 (12):1290-1299

6. Van Tulder MW, Assendelft WJJ, Koes BW, Bouter LM, Bombardier C, Nachemson AL, Esmail R, Deyo RA, Shekelle PG, Bouter LM, De Bie RA, Waddell G, Roland M, Guillemin F (1997) Method guidelines for systematic reviews in the Cochrane Collaboration Back Review Group for spinal disorders. Spine 22 (20):2323-2330

7. Moseley AM, Herbert RD, Sherrington C, Maher CG (2002) Evidence for physiotherapy practice: a survey of the Physiotherapy Evidence Database (PEDro). Australian Journal of Physiotherapy 48 (1):43-49

8. Sherrington C, Herbert RD, Maher CG, Moseley AM (2000) PEDro. A database of randomized trials and systematic reviews in physiotherapy. Manual therapy 5 (4):223-226

9. Bizzini M, Childs JD, Piva SR (2003) Systematic review of the quality of randomized controlled trials for patellofemoral pain syndrome. Journal of Orthopaedic & Sports Physical Therapy 33 (1):4-20
