# Supplementary material for: Randomized clinical trials in dentistry: Risks of bias, risks of random errors, reporting quality, and methodologic quality over the years 1955–2013
Source: PLoS One. 2017 Dec 22;12(12):e0190089. doi: 10.1371/journal.pone.0190089 (PMC5741237; doi:10.1371/journal.pone.0190089)
Supplement: S3 Appendix — (DOCX) [file pone.0190089.s003.docx]

| **Appendix S3. The Cochrane Collaboration’s tool for assessing risk of bias [**[**1**](#_ENREF_1)**,**[**2**](#_ENREF_2)**]** | | | |
| --- | --- | --- | --- |
| **Domain** | **Description** | **Risk of Bias** | **Consensus (circle)** |
| **Random sequence generation** |  | Was the allocation sequence adequately generated? | Low/High/Unclear |
| **Allocation concealment** |  | Was allocation adequately concealed? | Low/High/Unclear |
| **Blinding of participants and personnel** | *Subjective outcomes* | Was knowledge of the allocated intervention adequately prevented during the study? | Low/High/Unclear |
|  | *Objective outcomes* |  |  |
| **Blinding of outcome assessment** | *Subjective outcomes* | Was knowledge of the allocated intervention adequately prevented during the study? | Low/High/Unclear |
|  | *Objective outcomes* |  |  |
| **Incomplete outcome data** | *Subjective outcomes* | Were incomplete outcome data adequately addressed? | Low/High/Unclear |
|  | *Objective outcomes* |  |  |
| **Selective outcome reporting** |  | Are reports of the study free of suggestion of selective outcome reporting? | Low/High/Unclear |
| **Other sources of bias** |  | Was the study apparently free of other problems that could put it at a high risk of bias? | Low/High/Unclear |
| **Overall risk of bias** | Low/High/Unclear | | Low/High/Unclear |

**References for Appendix S3**

1. Higgins J, Altman D (2008) Chapter 8: Assessing risk of bias in included studies In: Higgins J, Green S (eds) Cochrane Handbook for Systematic Reviews of Interventions version 5.0. John Wiley & Sons, Ltd., Chichester, UK.

2. Higgins JPT, Altman DG, Goetzsche PC, Juni P, Moher D, Oxman AD, SavoviÄ J, Schulz KF, Weeks L, Sterne JAC (2011) The Cochrane Collaboration's tool for assessing risk of bias in randomised trials. BMJ 343 (7829).
