# Supplementary material for: Randomized clinical trials in dentistry: Risks of bias, risks of random errors, reporting quality, and methodologic quality over the years 1955–2013
Source: PLoS One. 2017 Dec 22;12(12):e0190089. doi: 10.1371/journal.pone.0190089 (PMC5741237; doi:10.1371/journal.pone.0190089)
Supplement: S4 Appendix — (DOCX) [file pone.0190089.s004.docx]

| **Appendix S4. Guidelines for evaluating the risk of bias of trials [**[**1**](#_ENREF_1)**,**[**2**](#_ENREF_2)**]** | |
| --- | --- |
| **RANDOM SEQUENCE GENERATION**  **Selection bias (biased allocation to interventions) due to inadequate generation of a randomised sequence.** | |
| Criteria for a judgement of  **‘Low risk’ of bias.** | The investigators describe a random component in the sequence generation process such as:   - Referring to a random number table; - Using a computer random number generator; - Coin tossing; - Shuffling cards or envelopes; - Throwing dice; - Drawing of lots; - Minimization*.   *Minimization may be implemented without a random element, and this is considered to be equivalent to being random. |
| Criteria for the judgement of ‘**High risk’** of bias. | The investigators describe a nonrandom component in the sequence generation process. Usually, the description would involve some systematic, nonrandom approach, for example:   - Sequence generated by odd or even date of birth; - Sequence generated by some rule based on date (or day) of admission; - Sequence generated by some rule based on hospital or clinic record number.   Other nonrandom approaches happen much less frequently than the systematic approaches mentioned above and tend to be obvious. They usually involve judgement or some method of nonrandom categorization of participants, for example:   - Allocation by judgement of the clinician; - Allocation by preference of the participant; - Allocation based on the results of a laboratory test or a series of tests; - Allocation by availability of the intervention. |
| Criteria for the judgement of ‘Unclear risk’ of bias. | Insufficient information about the sequence generation process to permit judgement of ‘Low risk’ or ‘High risk.’ |
| **ALLOCATION CONCEALMENT**  Selection bias (biased allocation to interventions) due to inadequate concealment of allocations prior to assignment. | |
| Criteria for a judgement of ‘Low risk’ of bias. | Participants and investigators enrolling participants could not foresee assignment because one of the following, or an equivalent method, was used to conceal allocation:   - Central allocation (including telephone, web-based and pharmacy-controlled randomization); - Sequentially numbered drug containers of identical appearance; - Sequentially numbered, opaque, sealed envelopes (3 characteristics need to be present) |
| Criteria for the judgement of ‘High risk’ of bias. | Participants or investigators enrolling participants could possibly foresee assignments and thus introduce selection bias, such as allocation based on:   - Using an open random allocation schedule (e.g. a list of random numbers); - Assignment envelopes were used without appropriate safeguards (e.g. if envelopes were unsealed or non­opaque or not sequentially numbered); - Alternation or rotation; - Date of birth; - Case record number; - Any other explicitly unconcealed procedure. |
| Criteria for the judgement of ‘Unclear risk’ of bias. | Insufficient information to permit judgement of ‘Low risk’ or ‘High risk.’ This is usually the case if the method of concealment is not described or not described in sufficient detail to allow a definite judgement – for example, if the use of assignment envelopes is described, but it remains unclear whether envelopes were sequentially numbered, opaque, and sealed. (Not all of the 3 characteristics are present). |
| **BLINDING OF PARTICIPANTS AND PERSONNEL (Main Outcome)**   - Blinding of participants/patients is a “must” when outcomes are subjective or self-reported. - When outcomes are measured by an assessor, then assessors should be blinded to group allocation. - When outcomes are automated [database] (there is no assessor involved) then, blinding of participants or assessors is not an issue   Performance bias due to knowledge of the allocated interventions by participants and personnel during the study. | |
| Criteria for a judgement of ‘Low risk’ of bias. | Any one of the following:   - No blinding or incomplete blinding, but the review authors judge that the outcome is not likely to be influenced by lack of blinding (Automated outcome or administrative) - Blinding of participants and key study personnel ensured, and it is unlikely that the blinding could have been broken - Objectives automatized outcomes coming from databases or hospital register office. |
| Criteria for the judgement of ‘High risk’ of bias. | Any one of the following:   - No blinding or incomplete blinding, and the outcome is likely to be influenced by lack of blinding; - Blinding of key study participants and personnel attempted, but likely that the blinding could have been broken, and the outcome is likely to be influenced by lack of blinding. |
| Criteria for the judgement of ‘Unclear risk’ of bias. | Any one of the following:   - Insufficient information to permit a judgement of ‘Low risk’ or ‘High risk’; - The study did not address the issue of blinding. |
| **BLINDING OF OUTCOME ASSESSMENT**  Detection bias due to knowledge of the allocated interventions by outcome assessors. | |
| Criteria for a judgement of ‘Low risk’ of bias. | Any one of the following:   - No blinding of outcome assessment, but the review authors judge that the outcome measurement is not likely to be influenced by lack of blinding; - Blinding of outcome assessment ensured, and unlikely that the blinding could have been broken. |
| Criteria for a judgement of ‘High risk’ of bias. | Any one of the following:   - No blinding of outcome assessment, and the outcome measurement is likely to be influenced by lack of blinding; - Blinding of outcome assessment, but likely that the blinding could have been broken and the outcome measurement is likely to be influenced by lack of blinding. |
| Criteria for the judgement of ‘Unclear risk’ of bias. | Any one of the following:   - Insufficient information to permit judgement of ‘Low risk’ or ‘High risk’; - The study did not address the issue of blinding. |
| **INCOMPLETE OUTCOME DATA**  Attrition bias due to amount, nature, or handling of incomplete outcome data. | |
| Criteria for a judgement of ‘Low risk’ of bias. | Any one of the following:   - No missing outcome data (All patients were accounted for in the analysis) - Reasons for missing outcome data are unlikely to be related to the outcome (for survival data, censoring is unlikely to introduce bias); - Missing outcome data are balanced in numbers across intervention groups, with similar reasons for missing data across groups; - For dichotomous outcome data, the proportion of missing outcomes compared with observed event risk not enough to have a clinically relevant impact on the intervention effect estimate; - For continuous outcome data, plausible effect size (difference in means or standardized difference in means) among missing outcomes not enough to have a clinically relevant impact on observed effect size; - Missing data have been imputed using appropriate methods. - If authors claimed that an intention-to-treat analysis was performed, raters should confirm that all patients entered were accounted for in the analysis (i.e., do not assume that a true intention-to-treat analysis was done). - If the numbers and reasons for withdrawal/drop-out were described and comparable across groups and the authors performed an intention to treat analysis with ≤ 20% drop outs, then score low risk of bias - If the numbers and reasons for withdrawal/drop-out were described and comparable across groups but the authors did not perform an ITT and the dropout rate was less than or equal to 10%, then score low risk of bias |
| Criteria for the judgement of ‘High risk’ of bias. | Any one of the following:   - Reason for missing outcome data is likely to be related to true outcome, with either imbalance in numbers or reasons for missing data across intervention groups; - For dichotomous outcome data, the proportion of missing outcomes compared with observed event risk is enough to induce clinically relevant bias in the intervention effect estimate; - For continuous outcome data, plausible effect size (difference in means or standardized difference in means) among missing outcomes is enough to induce clinically relevant bias in observed effect size; - ‘As-treated’ analysis is done with a substantial departure of the intervention received from the intervention assigned at randomization; - Potentially inappropriate application of simple imputation. - No Intention to treat (ITT) or ITT performed with > 20% drop outs |
| Criteria for the judgement of ‘Unclear risk’ of bias. | Any one of the following:   - Insufficient reporting of attrition/exclusions to permit judgement of ‘Low risk’ or ‘High risk’ (e.g. number randomized participants is not stated, no reasons for missing data are provided); - The study did not address this outcome. - No Intention to treat (ITT) but >10% and ≤ 20% drop outs. |
| **SELECTIVE REPORTING (Are outcomes reported in methods and results?)**  Reporting bias due to selective outcome reporting. | |
| Criteria for a judgement of ‘Low risk’ of bias. | Any of the following:   - The study protocol is available and all of the study’s prespecified (primary and secondary) outcomes that are of interest in the review have been reported in the manner prespecified; - The study protocol is not available but it is clear that the published reports include all expected outcomes, including those that were prespecified (convincing text of this nature may be uncommon). - Since we are not searching for protocols, outcomes reported in the methods section need to match those reported in the results section - If 0-30% of the secondary outcomes are not reported, score low risk. - The main outcome has to be included in both methods and results sections. |
| Criteria for the judgement of ‘High risk’ of bias. | Any one of the following:   - Not all of the study’s prespecified primary outcomes have been reported; - One or more primary outcomes is reported using measurements, analysis methods, or subsets of the data (e.g. subscales) that were not prespecified; - One or more reported primary outcomes were not prespecified (unless clear justification for their reporting is provided, such as an unexpected adverse effect); - One or more outcomes of interest in the review are reported incompletely so that they cannot be entered in a meta-analysis; - The study report fails to include results for a key outcome that would be expected to have been reported for such a study. - ≥ 70% of secondary outcomes were unreported (combining methods or results sections) - If the main outcome was not reported in the study, score a high risk of bias |
| Criteria for the judgement of ‘Unclear risk’ of bias. | - Insufficient information to permit judgement of ‘Low risk’ or ‘High risk.’ It is likely that the majority of studies will fall into this category. - If between 31%–69% of secondary outcomes are UNREPORTED (combining methods or results sections) score as unclear risk of bias |
| **OTHER BIAS**  Bias due to problems not covered elsewhere in the table. | |
| Criteria for a judgement of ‘Low risk’ of bias. | - The study appears to be free of other sources of bias. - **Influence of funder** was judged as appropriate if one of the following situation is met: 1) sponsor is acknowledged with clear statement regarding no involvement of a sponsor in trial conduct, data management /analysis, or co-authorship; 2) funding is coming from a governmental agency or foundation; 3) sponsor is acknowledged only as providing equipment or drug for the study but no one of the authors is paid by the company or the company had nothing to do with designing or analyzing the trial. |
| Criteria for the judgement of ‘High risk’ of bias. | There is at least one important risk of bias. For example, the study:   - Had a potential source of bias related to the specific study design used; or - Has been claimed to have been fraudulent; or - Had some other problem. - If the study has baseline imbalances regarding demographic factors, duration and severity of complaints, and value of main outcome measure(s) [[3](#_ENREF_3)]. - Imbalances in co-interventions: if the co-interventions were imbalances between groups or they were not similar between groups [[3](#_ENREF_3)]. - Compliance with treatment was not acceptable (very poor adherence with actual treatment: e.g. exercises performed) based on the reported intensity, duration, number and frequency of sessions for both the index intervention and control intervention(s). For example, physiotherapy treatment is usually administered over several sessions; therefore, it is necessary to assess how many sessions each patient attended. For single session interventions, this item is irrelevant [[3](#_ENREF_3)]. - **Influence of funder** was judged as inappropriate if sponsor is acknowledged with information provided that a co-author works for that company of that company was involved in conduct of the study. |
| Criteria for the judgement of ‘Unclear risk’ of bias. | There may be a risk of bias, but there is either:   - Insufficient information to assess whether an important risk of bias exists; or - Insufficient rationale or evidence that an identified problem will introduce bias. - **Influence of funder** was assessed as “unclear” if there is insufficient information to permit a judgment, or if there is no mentioning of funding source. |
| Total Scoring for RoB tool:  If any High Risk = High Risk of Bias  If any Unclear and NO High Risk = Unclear Risk of Bias  If all of the items are Low Risk = Low Risk of Bias | |

**References for Appendix S4**

1. Higgins J, Altman D (2008) Chapter 8: Assessing risk of bias in included studies In: Higgins J, Green S (eds) Cochrane Handbook for Systematic Reviews of Interventions version 5.0. John Wiley & Sons, Ltd., Chichester, UK,

2. Higgins JPT, Altman DG, Goetzsche PC, Juni P, Moher D, Oxman AD, SavoviÄ J, Schulz KF, Weeks L, Sterne JAC (2011) The Cochrane Collaboration's tool for assessing risk of bias in randomised trials. BMJ 343 (7829)

3. Furlan AD, Pennick V, Bombardier C, Van Tulder M (2009) Updated method guidelines for systematic reviews in the cochrane back review group. Spine 34 (18):1929-1941.
