# Supplementary material for: Randomized clinical trials in dentistry: Risks of bias, risks of random errors, reporting quality, and methodologic quality over the years 1955–2013
Source: PLoS One. 2017 Dec 22;12(12):e0190089. doi: 10.1371/journal.pone.0190089 (PMC5741237; doi:10.1371/journal.pone.0190089)
Supplement: S5 Appendix — (DOCX) [file pone.0190089.s005.docx]

**Appendix S5. The list of included trials**

Mitropoulos CM, Holloway PJ, Davies TG, Worthington HV. Relative efficacy of dentifrices containing 250 or 1000 ppm F-in preventing dental caries--report of a 32-month clinical trial. Community Dental Health. 1984;1(3):193-200.

Forsman B. Studies on the effect of dentifrices with low fluoride content. Community Dentistry and Oral Epidemiology. 1974;2(4):166-75.

Polansky R, Haas M, Heschl A, Wimmer G. Clinical effectiveness of photodynamic therapy in the treatment of periodontitis. Journal of Clinical Periodontology. 2009;36(7):575-80.

Allen PF, Thomason JM, Jepson NJA, Nohl F, Smith DG, Ellis J. A randomized controlled trial of implant-retained mandibular overdentures. Journal of Dental Research. 2006;85(6):547-51.

Heifetz SB, Meyers R, Kingman A. A comparison of the anticaries effectiveness of daily and weekly rinsing with sodium fluoride solutions: findings after two years. Pediatric Dentistry. 1981;3(1):17.

Moreira BW, Guimares LO, Vieira S, Piedade EF. Bochecho com fluor associado a fluoretacao da agua de abastecimento publico, na prevencao da carie dentaria. Revassocpaulcirdent. 1981;35(4):301 passim.

Kingman A. Caries-preventive effects of daily and weekly fluoride mouthrinsing in a fluoridated community: final results after 30 months. The Journal of the American Dental Association. 1982;105(6):1010-3.

Cuenin MF, Scheidt MJ, O'Neal RB, Strong SL, Pashley DH, Horner JA, et al. An in vivo study of dentin sensitivity: the relation of dentin sensitivity and the patency of dentin tubules. Journal of Periodontology. 1991;62(11):668-73.

Hodge HC, Holloway PJ, Davies TG, Worthington HV. Caries prevention by dentifrices containing a combination of sodium monofluorophosphate and sodium fluoride. Report of a 3-year clinical trial. British Dental Journal. 1980;149(7):201.

Lulic M, Leiggener Görög I, Salvi GE, Ramseier CA, Mattheos N, Lang NP. One‐year outcomes of repeated adjunctive photodynamic therapy during periodontal maintenance: a proof‐of‐principle randomized‐controlled clinical trial. Journal of Clinical Periodontology. 2009;36(8):661-6.

Yilmaz HG, Kurtulmus-Yilmaz S, Cengiz E. Long-term effect of diode laser irradiation compared to sodium fluoride varnish in the treatment of dentine hypersensitivity in periodontal maintenance patients: a randomized controlled clinical study. Photomedicine and Laser Surgery. 2011;29(11):721-5.

List T, Helkimo M, Andersson S, Carlsson GE. Acupuncture and occlusal splint therapy in the treatment of craniomandibular disorders. Part I. A comparative study. Swedish Dental Journal. 1992;16(4):125-41.

Lindhe J, Koch G. The effect of supervised oral hygiene on the gingivae of children. Journal of Periodontal Research. 1967;2(3):215-20.

Mouly S, Salom M, Tillet Y, Coudert A-C, Oberli F, Preshaw PM, et al. Management of xerostomia in older patients: a randomised controlled trial evaluating the efficacy of a new oral lubricant solution. Drugs and aging. 2007;24(11):957-66.

Wright E, Anderson G, Schulte J. A randomized clinical trial of intraoral soft splints and palliative treatment for masticatory muscle pain. Journal of Orofacial Pain. 1995;9(2).

Kreutzer DL. Brief cognitive-behavioral treatment for TMD pain: Long-term outcomes and moderators of treatment. Pain. 2010;151(1):110-6.

Schwarz F, Arweiler N, Georg T, Reich E. Desensitizing effects of an Er: YAG laser on hypersensitive dentine. Journal of Clinical Periodontology. 2002;29(3):211-5.

Quteish D, Dolby AE. The use of irradiated‐crosslinked human collagen membrane in guided tissue regeneration. Journal of Clinical Periodontology. 1992;19(7):476-84.

Danda AK, Tatiparthi MK, Narayanan V, Siddareddi A. Influence of primary and secondary closure of surgical wound after impacted mandibular third molar removal on postoperative pain and swelling—a comparative and split mouth study. Journal of Oral and Maxillofacial Surgery. 2010;68(2):309-12.

Rong WS, Bian JY, Wang WJ, De Wang J. Effectiveness of an oral health education and caries prevention program in kindergartens in China. Community Dentistry and Oral Epidemiology. 2003;31(6):412-6.

Aldridge JP, Lester V, Watts TLP, Collins A, Viberti G, Wilson RF. Single‐blind studies of the effects of improved periodontal health on metabolic control in Type 1 diabetes mellitus. Journal of Clinical Periodontology. 1995;22(4):271-5.

Higashi Y, Goto C, Hidaka T, Soga J, Nakamura S, Fujii Y, et al. Oral infection-inflammatory pathway, periodontitis, is a risk factor for endothelial dysfunction in patients with coronary artery disease. Atherosclerosis. 2009;206(2):604-10.

Prosper L, Redaelli S, Pasi M, Zarone F, Radaelli G, Gherlone EF. A randomized prospective multicenter trial evaluating the platform-switching technique for the prevention of postrestorative crestal bone loss. International Journal of Oral and Maxillofacial Implants. 2009;24(2).

Wilson L, Massoth D, Whitney C, Huggins KH, et al. Brief group cognitive-behavioral intervention for temporomandibular disorders. Pain. 1994;59(2):175-87.

Yilmaz HG, Kurtulmus-Yilmaz S, Cengiz E. Long-term effect of diode laser irradiation compared to sodium fluoride varnish in the treatment of dentine hypersensitivity in periodontal maintenance patients: a randomized controlled clinical study. Photomedicine and Laser Surgery. 2011;29(11):721-5.

Gallagher SJ, Glassgow I, Caldwell R. Self‐application of fluoride by rinsing. Journal of Public Health Dentistry. 1974;34(1):13-21.

Francetti L, Del Fabbro M, Basso M, Testori T, Weinstein R. Enamel matrix proteins in the treatment of intra‐bony defects. Journal of Clinical Periodontology. 2004;31(1):52-9.

Tan YK, L'Estrange PR, Luo YM, Smith C, Grant HR, Simonds AK, et al. Mandibular advancement splints and continuous positive airway pressure in patients with obstructive sleep apnoea: a randomized cross‐over trial. The European Journal of Orthodontics. 2002;24(3):239-

Dao TTT, Lavigne GJ, Charbonneau A, Feine JS, Lund JP. The efficacy of oral splints in the treatment of myofascial pain of the jaw muscles: a controlled clinical trial. Pain. 1994;56(1):85-94.

Katagiri S, Nitta H, Nagasawa T, Uchimura I, Izumiyama H, Inagaki K, et al. Multi-center intervention study on glycohemoglobin (hba1c) and serum, high-sensitivity CRP (hs-CRP) after local anti-infectious periodontal treatment in type 2 diabetic patients with periodontal disease. Diabetes Research and Clinical Practice. 2009;83(3):308-15.

Moran J, Addy M. The effects of a cetylpyridinium chloride prebrushing rinse as an adjunct to oral hygiene and gingival health. Journal of Periodontology. 1991;62(9):562-4.

Carr MP. Efficacy of an automated flossing device in different regions of the mouth. Journal of Periodontology. 2007;78(8):1529-37.

Mankodi S, Walker C, Conforti N, devizio W, mccool JJ, Volpe AR. Clinical effect of a triclosan-containing dentifrice on plaque and gingivitis: a six-month study. Clinical Preventive Dentistry. 1992;14(6):4-10.

Finkelstein P, Yost KG, Grossman E. Mechanical devices versus antimicrobial rinses in plaque and gingivitis reduction. Clinical Preventive Dentistry. 1990;12(3):8-11.

Cortellini P, Tonetti MS, Lang NP, Suvan JE, Zucchelli G, Vangsted T, et al. The simplified papilla preservation flap in the regenerative treatment of deep intrabony defects: clinical outcomes and postoperative morbidity. Journal of Periodontology. 2001;72(12):1702-12.

Iasella JM, Greenwell H, Miller RL, Hill M, Drisko C, Bohra AA, et al. Ridge preservation with freeze-dried bone allograft and a collagen membrane compared to extraction alone for implant site development: a clinical and histologic study in humans. Journal of Periodontology. 2003;74(7):990-9.

Rsing CK, Aass AM, Mavropoulos A, Gjermo P. Clinical and radiographic effects of enamel matrix derivative in the treatment of intrabony periodontal defects: a 12-month longitudinal placebo-controlled clinical trial in adult periodontitis patients. Journal of Periodontology. 2005;76(1):129-33.

Thomason JM, Lund JP, Chehade A, Feine JS. Patient satisfaction with mandibular implant overdentures and conventional dentures 6 months after delivery. International Journal of Prosthodontics. 2003;16(5).

Hughes JA, Jandt KD, Baker N, Parker D, Newcombe RG, Eisenburger M, et al. Further modification to soft drinks to minimise erosion. Caries Research. 2002;36(1):70-4.

Depaola PF. Combined use of a sodium fluoride prophylaxis paste and a spray containing acidulated sodium fluoride solution. The Journal of the American Dental Association. 1967;75(6):1407-11.

Hargreaves JA, Chester GG. Clinical trial among Scottish children of an anti‐caries dentifrice containing 2% sodium monofluorophosphate. Community Dentistry and Oral Epidemiology. 1973;1(2):47-57.

Fanning EA, Gotjamanos T, Vowles NJ. The use of fluoride dentifrices in the control of dental caries: Methodology and results of a clinical trial. Australian Dental Journal. 1968;13(3):201-6.

Zantner C, Popescu O, Martus P, Kielbassa AM. Randomized clinical study on the efficacy of a new lacquer for dentine hypersensitivity. Schweizer Monatsschrift fur Zahnmedizin. 2006;116(12):1232.

Stephen KW, Creanor SL, Russell JI, Burchell CK, Huntington E, Downie CFA. A 3‐year oral health dose‐response study of sodium monofluoro‐phosphate dentifrices with and without zinc citrate: anti‐caries results. Community Dentistry and Oral Epidemiology. 1988;16(6):321-5.

Dworkin SF, Turner JA, Wilson L, Massoth D, Whitney C, Huggins KH, et al. Brief group cognitive-behavioral intervention for temporomandibular disorders. Pain. 1994;59(2):175-87.

Jackson D, Sutcliffe P. Clinical testing of a stannous fluoride-calcium pyrophosphate dentifrice in Yorkshire school children. British Dental Journal. 1967;123:40.

Berggren U, Linde A. Dental fear and avoidance: a comparison of two modes of treatment. Journal of Dental Research. 1984;63(10):1223-

Cubells AB, Dalmau LB, Petrone ME, Chaknis P, Volpe AR. The effect of A Triclosan/copolymer/fluoride dentifrice on plaque formation and gingivitis: a six-month clinical study. The Journal of Clinical Dentistry. 1990;2(3):63-9.

Bolden TE, Zambon JJ, Sowinski J, Ayad F, mccool JJ, Volpe AR, et al. The clinical effect of a dentifrice containing triclosan and a copolymer in a sodium fluoride/silica base on plaque formation and gingivitis: a six-month clinical study. The Journal of Clinical Dentistry. 1991;3(4):125-31.

Apatzidou DA, Kinane DF. Quadrant root planing versus same‐day full‐mouth root planing. Journal of Clinical Periodontology. 2004;31(2):132-40.

Mateu FA, Boneta AE, devizio W, Stewart B, Proskin HM. A clinical investigation of the efficacy of two dentifrices for controlling established supragingival plaque and gingivitis. The Journal of Clinical Dentistry. 2008;19(3):85-94.

Rosling B, Hellström MK, Ramberg P, Socransky SS, Lindhe J. The use of PVP‐iodine as an adjunct to non‐surgical treatment of chronic periodontitis. Journal of Clinical Periodontology. 2001;28(11):1023-31.

Shern RJ, Duany LF, Senning RS, Zinner DD. Clinical study of an amine fluoride gel and acidulated phosphate fluoride gel. Community Dentistry and Oral Epidemiology. 1976;4(4):133-6.

Marks RG, Conti AJ, Moorhead JE, Cancro L, D'Agostino RB. Results from a three-year caries clinical trial comparing naf and SMFP fluoride formulations. International Dental Journal. 1994;44(3 Suppl 1):275-85.

Robinson PG, Pankhurst CL, Garrett EJ. Randomized‐controlled trial: effect of a reservoir biteguard on quality of life in xerostomia. Journal of oral pathology and medicine. 2005;34(4):193-7.

Shiloah J, Patters MR. DNA probe analyses of the survival of selected periodontal pathogens following scaling, root planing, and intra-pocket irrigation. Journal of Periodontology. 1994;65(6):568-75.

Velasquez-Plata D, Todd Scheyer E, Mellonig JT. Clinical comparison of an enamel matrix derivative used alone or in combination with a bovine-derived xenograft for the treatment of periodontal osseous defects in humans. Journal of Periodontology. 2002;73(4):433-40.

Jones JA, Miller DR, Wehler CJ, Rich SE, Krall‐Kaye EA, mccoy LC, et al. Does periodontal care improve glycemic control? The department of veterans affairs dental diabetes study. Journal of Clinical Periodontology. 2007;34(1):46-52.

Akota I, Alvsaker B, Bjrnland T. The effect of locally applied gauze drain impregnated with chlortetracycline ointment in mandibular third-molar surgery. Acta Odontologica Scandinavica. 1998;56(1):25-9.

Lundh H, Westesson P-L, Kopp S, Tillstrm B. Anterior repositioning splint in the treatment of temporomandibular joints with reciprocal clicking: comparison with a flat occlusal splint and an untreated control group. Oral Surgery, Oral Medicine, Oral Pathology. 1985;60(2):131-6.

Döri F, Nikolidakis D, Huszar T, Arweiler NB, Gera I, Sculean A. Effect of platelet‐rich plasma on the healing of intrabony defects treated with an enamel matrix protein derivative and a natural bone mineral. Journal of Clinical Periodontology. 2008;35(1):44-50.

Bellissimo-Rodrigues F, Bellissimo-Rodrigues WT, Viana JM, Teixeira GCA, Nicolini E, Auxiliadora-Martins M, et al. Effectiveness of oral rinse with chlorhexidine in preventing nosocomial respiratory tract infections among intensive care unit patients. Infection Control and Hospital Epidemiology. 2009;30(10):952-8.

Lundh H, Westesson P-L, Eriksson L, Brooks SL. Temporomandibular joint disk displacement without reduction: treatment with flat occlusal splint versus no treatment. Oral Surgery, Oral Medicine, Oral Pathology. 1992;73(6):655-8.

Spahr A, Haegewald S, Tsoulfidou F, Rompola E, Heijl L, Bernimoulin J-P, et al. Coverage of Miller class I and II recession defects using enamel matrix proteins versus coronally advanced flap technique: a 2-year report. Journal of Periodontology. 2005;76(11):1871-80.

Fogels HR, Alman JE, Meade JJ, O’Donnell JP. The relative caries-inhibiting effects of a stannous fluoride dentifrice in a silica gel base. The Journal of the American Dental Association. 1979;99(3):456-9.

Osunde OD, Adebola RA, Saheeb BD. A comparative study of the effect of suture-less and multiple suture techniques on inflammatory complications following third molar surgery. International Journal of Oral and Maxillofacial Surgery. 2012;41(10):1275-9.

Isik B, Baygin O, Bodur H. Premedication with melatonin vs midazolam in anxious children. Pediatric Anesthesia. 2008;18(7):635-41.

Whittle JG, Whitehead HF, Bishop CM. A randomised control trial of oral health education provided by a health visitor to parents of pre-school children. Community Dental Health. 2008;25(1):28-32.

Kuru B, Yılmaz S, Argın K, Noyan Ü. Enamel matrix derivative alone or in combination with a bioactive glass in wide intrabony defects. Clinical oral investigations. 2006;10(3):227-34.

Froum SJ, Weinberg MA, Tarnow D. Comparison of bioactive glass synthetic bone graft particles and open debridement in the treatment of human periodontal defects. A clinical study. Journal of Periodontology. 1998;69(6):698-709.

Borghetti A, Glise J-M, Monnet-Corti V, Dejou J. Comparative clinical study of a bioabsorbable membrane and subepithelial connective tissue graft in the treatment of human gingival recession. Journal of Periodontology. 1999;70(2):123-30

Gallardo F, Cornejo G, Borie R. Oral midazolam as premedication for the apprehensive child before dental treatment. The Journal of Clinical Pediatric Dentistry. 1994;18(2):123-7.

Minor V, Marris CK, mcgorray SP, Yezierski R, Fillingim R, Logan H, et al. Effects of preoperative ibuprofen on pain after separator placement. American Journal of Orthodontics and Dentofacial Orthopedics. 2009;136(4):510-7.

Mcmillan AS, Tsang CSP, Wong MCM, Kam AYL. Efficacy of a novel lubricating system in the management of radiotherapy-related xerostomia. Oral oncology. 2006;42(8):842-8.

Heifetz SB, Driscoll WS, Creighton WE. The effect on dental caries of weekly rinsing with a neutral sodium fluoride or an acidulated phosphate-fluoride mouthwash. The Journal of the American Dental Association. 1973;87(2):364-8.

Nagy K, Urban E, Fazekas O, Thurzo L, Nagy E. Controlled study of lactoperoxidase gel on oral flora and saliva in irradiated patients with oral cancer. Journal of Craniofacial Surgery. 2007;18(5):1157-64.

Ingraham RQ, Williams JE. An evaluation of the utility of application and cariostatic effectiveness of phosphate-fluorides in solution and gel states. Journal-Tennessee State Dental Association. 1970;50(1):5-12.

O'Mullane DM, Kavanagh D, Ellwood RP, Chesters RK, Schafer F, Huntington E, et al. A three-year clinical trial of a combination of trimetaphosphate and sodium fluoride in silica toothpastes. Journal of Dental Research. 1997;76(11):1776-81.

Williams K, Rapley K, Haun J, Walters P, He T, Grender J, et al. Comparison of rotation/oscillation and sonic power toothbrushes on plaque and gingivitis for 10 weeks. American Journal of Dentistry. 2009;22(6):345.

Borghetti A, Novakovitch G, Louise F, Simeone D, Fourel J. Cryopreserved cancellous bone allograft in periodontal intraosseous defects. Journal of Periodontology. 1993;64(2):128-32.

Hill HC, Levi PA, Glickman I. The effects of waxed and unwaxed dental floss on interdental plaque accumulation and interdental gingival health. Journal of Periodontology. 1973;44(7):411-3.

Vogel RI, Sullivan AJ, Pascuzzi JN, Deasy MJ. Evaluation of cleansing devices in the maintenance of interproximal gingival health. Journal of Periodontology. 1975;46(12):745-7.

Mokhlis GR, Matis BA, Cochran MA, Eckert GJ. A clinical evaluation of carbamide peroxide and hydrogen peroxide whitening agents during daytime use. The Journal of the American Dental Association. 2000;131(9):1269-77.

Rabalais Jr ML, Yukna RA, Mayer ET. Evaluation of Durapatite Ceramic as an Alloplastic Implant in Periodontal Osseous Defects: I. Initial Six-Month Results. Journal of Periodontology. 1981;52(11):680-9.

Moreira BH, Tumang AJ. Prevention of dental caries by means of mouthwashes with 0.1 solutions of sodium fluoride. Results of a 2 year study. Revista brasileira de odontologia. 1972;29(173):37.

Gillam DG, Coventry JF, Manning RH, Newman HN, Bulman JS. Comparison of two desensitizing agents for the treatment of cervical dentine sensitivity. Dental Traumatology. 1997;13(1):36-9.

Jared H, Zhong Y, Rowe M, Ebisutani K, Tanaka T, Takase N. Clinical trial of a novel interdental brush cleaning system. The Journal of Clinical Dentistry. 2004;16(2):47-52.

Pillon FL, Romani IG, Schmidt dr. Effect of a 3% potassium oxalate topical application on dentinal hypersensitivity after subgingival scaling and root planing. Journal of Periodontology. 2004;75(11):1461-4

Preshaw PM, Grainger P, Bradshaw MH, Mohammad AR, Powala CV, Nolan A. Subantimicrobial dose doxycycline in the treatment of recurrent oral aphthous ulceration: a pilot study. Journal of oral pathology and medicine. 2007;36(4):236-40.

Beiswanger BB, mcclanahan SF, Bartizek RD, Lanzalaco AC, Bacca LA, White DJ. The comparative efficacy of stabilized stannous fluoride dentifrice, peroxide/baking soda dentifrice and essential oil mouthrinse for the prevention of gingivitis. The Journal of Clinical Dentistry. 1996;8(2 Spec No):46-53.

Andlaw RJ, Tucker GJ. A dentifrice containing 0.8 per cent sodium monofluorophosphate in an aluminium oxide trihydrate base. A 3-year clinical trial. British Dental Journal. 1975;138(11):426-3

Awad MA, Lund JP, Shapiro SH, Locker D, Klemetti E, Chehade A, et al. Oral health status and treatment satisfaction with mandibular implant overdentures and conventional dentures: a randomized clinical trial in a senior population. International Journal of Prosthodontics. 2003;16(4).

Christodoulides N, Nikolidakis D, Chondros P, Becker J, Schwarz F, Rssler R, et al. Photodynamic therapy as an adjunct to non-surgical periodontal treatment: a randomized, controlled clinical trial. Journal of Periodontology. 2008;79(9):1638-44.

Cannizzaro G, Felice P, Leone M, Checchi V, Esposito M. Flapless versus open flap implant surgery in partially edentulous patients subjected to immediate loading: 1-year results from a split-mouth randomised controlled trial. European Journal of Oral Implantology. 2011;4(3):177-88.

Cortellini P, Prato GP, Tonetti MS. Periodontal regeneration of human intrabony defects with bioresorbable membranes. A controlled clinical trial. Journal of Periodontology. 1996;67(3):217-23.

Van Wyk I, van Wyk CW. The effectiveness of a 0.2 percent and a 0.05 percent neutral naf mouthrinsing programme. The Journal of the Dental Association of South Africa= Die Tydskrif van die Tandheelkundige Vereniging van Suid-Afrika. 1986;41(2):35.

Schubert MM, Eduardo FP, Guthrie KA, Franquin J-C, Bensadoun R-JJ, Migliorati CA, et al. A phase III randomized double-blind placebo-controlled clinical trial to determine the efficacy of low level laser therapy for the prevention of oral mucositis in patients undergoing hematopoietic cell transplantation. Supportive Care in Cancer. 2007;15(10):1145-54.

Mainwaring PJ, Naylor MN. A three-year clinical study to determine the separate and combined caries-inhibiting effects of sodium monofluorophosphate toothpaste and an acidulated phosphate-fluoride gel. Caries Research. 1978;12(4):202-12.

Cobb HB, Rozier RG, Bawden JW. A clinical study of the caries preventive effects of an APF solution and an APF thixotropic gel. Journal of Pediatric Dentistry. 1980;2(4):263-6.

Slack GL, Berman DS, Martin WJ, Young J. Clinical testing of a stannous fluoride-insoluble metaphosphate dentifrice in Kent school girls. British Dental Journal. 1967;123:9-16.

Harper PR, Milsom S, Wade W, Addy M, Moran J, Newcombe RG. An approach to efficacy screening of mouthrinses: studies on a group of French products. Journal of Clinical Periodontology. 1995;22(9):723-7.

Barone A, Aldini NN, Fini M, Giardino R, Calvo Guirado JL, Covani U. Xenograft versus extraction alone for ridge preservation after tooth removal: a clinical and histomorphometric study. Journal of Periodontology. 2008;79(8):1370-7.

Brudevold F, Chilton NW. Comparative study of a fluoride dentifrice containing soluble phosphate and a calcium-free abrasive: second-year report. The Journal of the American Dental Association. 1966;72(4):889-94.

Turner JA, Mancl L, Aaron LA. Short-and long-term efficacy of brief cognitive-behavioral therapy for patients with chronic temporomandibular disorder pain: a randomized, controlled trial. Pain. 2006;121(3):181-94.

Spets‐Happonen S, Luoma H, Forss H, Kentala J, Alaluusua S, Luoma AR, et al. Effects of a chlorhexidine‐fluoride‐strontium rinsing program on caries, gingivitis and some salivary bacteria among Finnish school children. European Journal of Oral Sciences. 1991;99(2):130-8.

Peterson JK, Williamson L. Field test of a sodium fluoride dentifrice containing acid orthophosphate and an insoluble metaphosphate abrasive--second year report. Journal of oral therapeutics and pharmacology. 1967;4(1):1.

Mela L, Bracco P. Primary and secondary closure of the surgical wound after removal of impacted mandibular third molars: a comparative study. International Journal of Oral and Maxillofacial Surgery. 2005;34(1):52-7.

Pradeep AR, Agarwal E, Naik SB. Clinical and microbiologic effects of commercially available dentifrice containing aloe vera: a randomized controlled clinical trial. Journal of Periodontology. 2012;83(6):797-804.

Barnes GP, Roberts DW, Katz RV, Woolridge Jr ED. Effects of two cetylpyridinium chloride-containing mouthwashes on bacterial plaque. Journal of Periodontology. 1976;47(7):419-22.

Kemppainen P, Eskola S, Ylipaavalniemi P. A comparative prospective clinical study of two single-tooth implants: a preliminary report of 102 implants. The Journal of Prosthetic Dentistry. 1997;77(4):382-7.

Wallenstein S, Fleiss JL, Chilton NW. Confidence intervals for percentage reduction in caries increments. Journal of Dental Research. 1982;61(6):828-30.

Charles CH, Sharma NC, Galustians HJ, Qaqish J, mcguire JA, Vincent JW. Comparative efficacy of an antiseptic mouthrinse and an antiplaque/antigingivitis dentifrice: a six-month clinical trial. The Journal of the American Dental Association. 2001;132(5):670-5.

Güncü GN, Tözüm TF, Güncü MB, Yamalik N. Relationships between implant stability, image‐based measures and nitric oxide levels. Journal of Oral Rehabilitation. 2008;35(10):745-53.

Gerlach RW, Zhou X. Comparative clinical efficacy of two professional bleaching systems. COMPENDIUM-NEWTOWN-. 2002;23(1A):35-41.

Yilmaz HG, Kurtulmus-Yilmaz S, Cengiz E, Bayindir H, Aykac Y. Clinical evaluation of Er, Cr: YSGG and gaalas laser therapy for treating dentine hypersensitivity: A randomized controlled clinical trial. Journal of Dentistry. 2011;39(3):249-54.

Zucchelli G, Bernardi F, Montebugnoli L, Sanctis MD. Enamel Matrix Proteins and Guided Tissue Regeneration With Titanium-Reinforced Expanded polytetrafluoroethylenemembranes in the Treatment of Infrabony Defects: A Comparative Controlled Clinical Trial. Journal of Periodontology. 2002;73(1):3-12.

Trammell K, Geurs NC, O'Neal SJ, Liu P-R, Haigh SJ, mcneal S, et al. A prospective, randomized, controlled comparison of platform-switched and matched-abutment implants in short-span partial denture situations. International Journal of Periodontics and Restorative Dentistry. 2009;29(6).

Packer MW, Laswell HR, Doyle J, Naff HH, Brown F. Cariostatic effects of fluoride mouthrinses in a non-fluoridated community. The Journal of the Tennessee Dental Association. 1975;55(1):22.

Turkyilmaz I, Tumer C. Early versus late loading of unsplinted tiunite surface implants supporting mandibular overdentures: a 2‐year report from a prospective study. Journal of Oral Rehabilitation. 2007;34(10):773-80.

Meffert RM, Thomas JR, Hamilton KM, Brownstein CN. Hydroxylapatite as an alloplastic graft in the treatment of human periodontal osseous defects. Journal of Periodontology. 1985;56(2):63-73.

Zucchelli G, Bernardi F, Montebugnoli L, Sanctis MD. Enamel Matrix Proteins and Guided Tissue Regeneration With Titanium-Reinforced Expanded polytetrafluoroethylenemembranes in the Treatment of Infrabony Defects: A Comparative Controlled Clinical Trial. Journal of Periodontology. 2002;73(1):3-12.

Mortazavi M, Pourhashemi SJ, Khosravi MB, Ashtari S, Ghaderi F. Assessment of a low dose of IV midazolam used orally for conscious sedation in pediatric dentistry. DARU Journal of Pharmaceutical Sciences. 2015;17(2):79-82.

Lekovic V, Camargo PM, Klokkevold PR, Weinlaender M, Kenney EB, Dimitrijevic B, et al. Preservation of alveolar bone in extraction sockets using bioabsorbable membranes. Journal of Periodontology. 1998;69(9):1044-9.

Cannizzaro G, Leone M. Restoration of partially edentulous patients using dental implants with a microtextured surface: a prospective comparison of delayed and immediate full occlusal loading. International Journal of Oral and Maxillofacial Implants. 2003;18(4).

Trombelli L, Scabbia A, Tatakis DN, Calura G. Subpedicle connective tissue graft versus guided tissue regeneration with bioabsorbable membrane in the treatment of human gingival recession defects. Journal of Periodontology. 1998;69(11):1271-7.

Renvert S, Birkhed D. Comparison between 3 triclosan dentifrices on plaque, gingivitis and salivary microflora. Journal of Clinical Periodontology. 1995;22(1):63-70.

Mestnik MJ, Feres M, Figueiredo LC, Duarte PM, Lira EAG, Faveri M. Short‐term benefits of the adjunctive use of metronidazole plus amoxicillin in the microbial profile and in the clinical parameters of subjects with generalized aggressive periodontitis. Journal of Clinical Periodontology. 2010;37(4):353-65.

Zucchelli G, Clauser C, De Sanctis M, Calandriello M. Mucogingival versus guided tissue regeneration procedures in the treatment of deep recession type defects. Journal of Periodontology. 1998;69(2):138-45.

Meijer HJA, Raghoebar GM, Van‘t Hof MA. Comparison of implant-retained mandibular overdentures and conventional complete dentures: a 10-year prospective study of clinical aspects and patient satisfaction. International Journal of Oral and Maxillofacial Implants. 2003;18(6).

Robinson PJ, Maddalozzo D, Breslin S. A six-month clinical comparison of the efficacy of the Sonicare and the Braun Oral-B electric toothbrushes on improving periodontal health in adult periodontitis patients. The Journal of Clinical Dentistry. 1997;8(1 Spec No):4-9.

Johansson A, Wenneberg B, Wagersten C, Haraldson T. Acupuncture in treatment of facial muscular pain. Acta Odontologica Scandinavica. 1991;49(3):153-8.

Yukna RA. HTR polymer grafts in human periodontal osseous defects. I. 6-month clinical results. Journal of Periodontology. 1990;61(10):633-42.

Higashi Y, Goto C, Jitsuiki D, Umemura T, Nishioka K, Hidaka T, et al. Periodontal infection is associated with endothelial dysfunction in healthy subjects and hypertensive patients. Hypertension. 2008;51(2):446-53.

Kiger RD, Nylund K, Feller RP. A comparison of proximal plaque removal using floss and interdental brushes. Journal of Clinical Periodontology. 1991;18(9):681-4.

Fogels HR, Meade JJ, Griffith J, Miragliuolo R, Cancro LP. A clinical investigation of a high-level fluoride dentifrice. ASDC Journal of Dentistry for Children. 1988;55(3):210.

Kim C-K, Choi E-J, Cho K-S, Chai J-K, Wikesj UME. Periodontal repair in intrabony defects treated with a calcium carbonate implant and guided tissue regeneration. Journal of Periodontology. 1996;67(12):1301-6.

Xavier RL, Vasconcelos BC, Caubi AF, Porto GG, Maurette MA. Passive drainage through the vestibular oblique incision in impacted inferior third molar surgery: a preliminary study. Acta odontologica latinoamericana: AOL. 2007;21(1):57-63.

Tawse‐Smith A, Payne AGT, Kumara R, Thomson WM. Early Loading of Unsplinted Implants Supporting Mandibular Overdentures Using a One‐Stage Operative Procedure with Two Different Implant Systems: A 2‐Year Report. Clinical Implant Dentistry and Related Research. 2002;4(1):33-42.

Zacherl WA. A clinical evaluation of a stannous fluoride and a sarcosinate dentifrice. ASDC Journal of Dentistry for Children. 1973;40(6):451.

Zamet JS, Darbar UR, Griffiths GS, Bulman JS, Brägger U, Bürgin W, et al. Particulate bioglass® as a grafting material in the treatment of periodontal intrabony defects. Journal of Clinical Periodontology. 1997;24(6):410-8.

Gerlach RW, Gibb RD, Sagel PA. A randomized clinical trial comparing a novel 5.3% hydrogen peroxide whitening strip to 10%, 15%, and 20% carbamide peroxide tray-based bleaching systems. Compendium of Continuing Education in Dentistry (Jamesburg, NJ: 1995)Supplement. 1999(29):3.

Piemontese M, Aspriello SD, Rubini C, Ferrante L, Procaccini M. Treatment of periodontal intrabony defects with demineralized freeze-dried bone allograft in combination with platelet-rich plasma: a comparative clinical trial. Journal of Periodontology. 2008;79(5):802-10.

Ringelberg ML, Conti AJ, Ward CB, Clark B, Lotzkar S, editors. Effectiveness of Different Concentrations And Frequencies Of Sodium-fluoride Mouthrinse. Journal of Dental Research; 198, Va 22314

Castillo JL, Rivera S, Aparicio T, Lazo R, Aw TC, Mancl LL, et al. The short-term effects of diammine silver fluoride on tooth sensitivity: a randomized controlled trial. Journal of Dental Research. 2011;90(2):203-8.

Mcbride MA, Gilpatrick RO, Fowler WL. The effectiveness of sodium fluoride iontophoresis in patients with sensitive teeth. Quintessence international. 1991;22(8).

Lovelace TB, Mellonig JT, Meffert RM, Jones AA, Nummikoski PV, Cochran DL. Clinical evaluation of bioactive glass in the treatment of periodontal osseous defects in humans. Journal of Periodontology. 1998;69(9):1027-35.

Chukwuneke FN, Oji C, Saheeb DB. A comparative study of the effect of using a rubber drain on postoperative discomfort following lower third molar surgery. International Journal of Oral and Maxillofacial Surgery. 2008;37(4):341-4.

Koromantzos PA, Makrilakis K, Dereka X, Offenbacher S, Katsilambros N, Vrotsos IA, et al. Effect of non-surgical periodontal therapy on C-reactive protein, oxidative stress, and matrix metalloproteinase (MMP)-9 and MMP-2 levels in patients with type 2 diabetes: a randomized controlled study. Journal of Periodontology. 2012;83(1):3-10.

Aranha ACC, de Paula Eduardo C. Effects of Er: YAG and Er, Cr: YSGG lasers on dentine hypersensitivity. Short-term clinical evaluation. Lasers in medical science. 2012;27(4):813-8.

Crespi R, Cappar P, Gherlone E, Romanos GE. Immediate versus delayed loading of dental implants placed in fresh extraction sockets in the maxillary esthetic zone: a clinical comparative study. International Journal of Oral and Maxillofacial Implants. 2008;23(4).

Ruiken R, Truin GJ, Konig K, Vogels A, Hof M. Clinical cariostatic effectiveness of a naf rinse in a low prevalent‐child population. Community Dentistry and Oral Epidemiology. 1987;15(2):57-9.

Grusovin MG, Esposito M. The efficacy of enamel matrix derivative (Emdogain) for the treatment of deep infrabony periodontal defects: a placebo-controlled randomised clinical trial. European Journal of Oral Implantology. 2009;2(1).

Zimmer S, Kolbe C, Kaiser G, Krage T, Ommerborn M, Barthel C. Clinical efficacy of flossing versus use of antimicrobial rinses. Journal of Periodontology. 2006;77(8):1380-5.

Biesbrock AR, Bartizek RD, Gerlach RW, Terzhalmy GT. Oral hygiene regimens, plaque control, and gingival health: a two-month clinical trial with antimicrobial agents. Journal of Clinical Dentistry. 2007;18(4):101.

Maiya GA, Sagar MS, Fernandes D. Effect of low level helium-neon (He-Ne) laser therapy in the prevention and treatment of radiation induced mucositis in head and neck cancer patients. Indian Journal of Medical Research. 2006;124(4):399.

Gurinsky BS, Mills MP, Mellonig JT. Clinical evaluation of demineralized freeze-dried bone allograft and enamel matrix derivative versus enamel matrix derivative alone for the treatment of periodontal osseous defects in humans. Journal of Periodontology. 2004;75(10):1309-18.

Tewari A, Chawla HS, Utreja A. Comparative evaluation of the role of naf, APF and Duraphat topical fluoride applications in the prevention of dental caries--a 2 1/2 years study. Journal of the Indian Society of Pedodontics and Preventive Dentistry. 1991;8(1):28-35.

Ferguson KA, Ono T, Lowe AA, Keenan SP, Fleetham JA. A randomized crossover study of an oral appliance vs nasal-continuous positive airway pressure in the treatment of mild-moderate obstructive sleep apnea. Chest. 1996;109(5):1269-75.

Englander HR, Keyes PH, Gestwicki M, Sulit HA. Clinical anticaries effect of repeated topical sodium fluoride applications by mouthpieces. The Journal of the American Dental Association. 1967;75(3):638-44.

Cons NC, Janerich DT, Senning RS. Albany topical fluoride study. The Journal of the American Dental Association. 1970;80(4):777-81.

Graziani F, d’aiuto F, Arduino PG, Tonelli M, Gabriele M. Perioperative dexamethasone reduces post-surgical sequelae of wisdom tooth removal. A split-mouth randomized double-masked clinical trial. International Journal of Oral and Maxillofacial Surgery. 2006;35(3):241-6.

Polat O, Karaman AI, Durmus E. Effects of preoperative ibuprofen and naproxen sodium on orthodontic pain. The Angle Orthodontist. 2005;75(5):791-6.

Meloni SM, De Riu G, Pisano M, De Riu N, Tullio A. Immediate versus delayed loading of single mandibular molars. One-year results from a randomised controlled trial. European Journal of Oral Implantology. 2012;5(4):345-53.

Batenburg RHK, Meijer HJA, Raghoebar GM, Van Oort RP, Boering G. Mandibular overdentures supported by two Brånemark, IMZ or ITI implants. A prospective comparative preliminary study: One‐year results. Clinical Oral Implants Research. 1998;9(6):374-83.

Forabosco A, Spinato S, Grandi T, Prini M. A comparative study between different techniques in non-surgical periodontal treatment. Minerva stomatologica. 2006;55(5):289-96.

Smith RG, Moran J, Addy M, Doherty F, Newcombe RG. Comparative staining in vitro and plaque inhibitory properties in vivo of 0.12% and 0.2% chlorhexidine mouthrinses. Journal of Clinical Periodontology. 1995;22(8):613-1

Castellanos T A, de la Rosa R M, de la Garza M, Caffesse RG. Enamel matrix derivative and coronal flaps to cover marginal tissue recessions. Journal of Periodontology. 2006;77(1):7-14.

Pakfetrat A, Mansourian A, Momen-Heravi F, Delavarian Z, Momen-Beitollahi J, Khalilzadeh O, et al. Comparison of colchicine versus prednisolone in recurrent aphthous stomatitis: A double-blind randomized clinical trial. Clinical and Investigative Medicine. 2010;33(3):189-95.

Heidmann J, Poulsen S, Arnbjerg D, Kirkegaard E, Laurberg L. Caries development after termination of a fluoride rinsing program. Community Dentistry and Oral Epidemiology. 1992;20(3):118-21.

Lpez NJ, Quintero A, Casanova PA, Ibieta CI, Baelum V, Lpez R. Effects of periodontal therapy on systemic markers of inflammation in patients with metabolic syndrome: a controlled clinical trial. Journal of Periodontology. 2012;83(3):267-78.

Borutta A, Knzel W, Rbsam F. The caries-protective efficacy of 2 fluoride varnishes in a 2-year controlled clinical trial. Deutsche Zahn-, Mund-, und Kieferheilkunde mit Zentralblatt. 1991;79(7):543-9.

Tatakis DN, Trombelli L. Gingival recession treatment: guided tissue regeneration with bioabsorbable membrane versus connective tissue graft. Journal of Periodontology. 2000;71(2):299-307.

Ozen T, Orhan K, Avsever H, Tunca YM, Ulker AE, Akyol M. Dentin hypersensitivity: a randomized clinical comparison of three different agents in a short-term treatment period. Operative Dentistry. 2009;34(4):392-8.

Muhler JC. A clinical comparison of fluoride and antienzyme dentifrices. ASDC Journal of Dentistry for Children. 1969;37(6):501 passim.

Kapur A, Chawla SH, Goyal A, Gauba K, Bhardwaj N. Efficacy and acceptabilty of oral-transmucosal midazolam as a conscious sedation agent in pre-school children. Journal of the Indian Society of Pedodontics and Preventive Dentistry. 2004;22(3):109-13.

Doroschak AM, Bowles WR, Hargreaves KM. Evaluation of the combination of flurbiprofen and tramadol for management of endodontic pain. Journal of Endodontics. 1999;25(10):660-3.

Rule JT, Smith MR, Truelove RB, Macko DJ, Castaldi CR. Caries inhibition of a dentifrice containing 0.78% sodium monofluorophosphate in a silica base. Community Dentistry and Oral Epidemiology. 1984;12(4):213-7.

Orhan K, Aksoy U, Can-Karabulut DC, Kalender A. Low-level laser therapy of dentin hypersensitivity: a short-term clinical trial. Lasers in Medical Science. 2011;26(5):591-8.

Femiano F, Buonaiuto C, Gombos F, Lanza A, Cirillo N. Pilot study on recurrent aphthous stomatitis (RAS): a randomized placebo-controlled trial for the comparative therapeutic effects of systemic prednisone and systemic montelukast in subjects unresponsive to topical therapy. Oral Surgery, Oral Medicine, Oral Pathology, Oral Radiology, and Endodontology. 2010;109(3):402-7.

Lier BB, Rsing CK, Aass AM, Gjermo P. Treatment of dentin hypersensitivity by Nd: YAG laser. Journal of Clinical Periodontology. 2002;29(6):501-6.

Pereira JC, Martineli ACBF, Santiago SL. Treating hypersensitive dentin with three different potassium oxalate-based gel formulations: a clinical study. Revfacodontolbauru. 2001;9(3/4):123-30.

Glass RL, Peterson JK, Bixler D. The effects of changing caries prevalence and diagnostic criteria on clinical caries trials. Caries Research. 1983;17(2):145-51.

Den Hartog L, Raghoebar GM, Stellingsma K, Vissink A, Meijer HJA. Immediate non‐occlusal loading of single implants in the aesthetic zone: a randomized clinical trial. Journal of Clinical Periodontology. 2011;38(2):186-94.

Assis jsd, Rodrigues LKA, Fonteles CSR, Colares RCR, Souza ambd, Santiago SL. Dentin hypersensitivity after treatment with desensitizing agents: a randomized, double-blind, split-mouth clinical trial. Brazilian Dental Journal. 2011;22(2):157-61.

Lu KH, Ruhlman CD, Chung KL, Sturzenberger OP, Lehnhoff RW. A three-year clinical comparison of a sodium monofluorophosphate dentifrice with sodium fluoride dentifrices on dental caries in children. ASDC Journal of Dentistry for Children. 1987;54(4):241-4

Joly JC, Palioto DB, Lima afmd, Mota LF, Caffesse R. Clinical and Radiographic Evaluation Periodontal Intrabony Defects Treated With Guided Tissue Regeneration. A Pilot Study. Journal of Periodontology. 2002;73(4):353-9.

Laswell HR, Packer MW, Wiggs JS. Cariostatic effects of fluoride mouthrinses in a fluoridated community. Journal of the Kentucky Dental Association. 1975;27(4):21.

Ge L, Shu R, Li Y, Li C, Luo L, Song Z, et al. Adjunctive effect of photodynamic therapy to scaling and root planing in the treatment of chronic periodontitis. Photomedicine and Laser Surgery. 2011;29(1):33-7.

Sharma N, Charles CH, Lynch MC, Qaqish J, mcguire JA, Galustians JG, et al. Adjunctive benefit of an essential oil–containing mouthrinse in reducing plaque and gingivitis in patients who brush and floss regularly: a six-month study. The Journal of the American Dental Association. 2004;135(4):496-504.

West NX, Hughes JA, Parker DM, Newcombe RG, Addy M. Development and evaluation of a low erosive blackcurrant juice drink 2. Comparison with a conventional blackcurrant juice drink and orange juice. Journal of dentistry. 1999;27(5):341-4.

Bryan ET, Williams JE. The cariostatic effectiveness of a phosphate‐fluoride gel administered annually to school children; final results. Journal of Public Health Dentistry. 1970;30(1):13-6.

Tarbet WJ, Silverman G, Stolman JM, Fratarcangelo PA. An evaluation of two methods for the quantitation of dentinal hypersensitivity. The Journal of the American Dental Association. 1979;98(6):914-8.

O'Beirne G, Johnson RH, Persson GR, Spektor MD. Efficacy of a sonic toothbrush on inflammation and probing depth in adult periodontitis. Journal of Periodontology. 1996;67(9):900-8.

Canullo L, Iannello G, Gtz W. The influence of individual bone patterns on peri-implant bone loss: preliminary report from a 3-year randomized clinical and histologic trial in patients treated with implants restored with matching-diameter abutments or the platform-switching concept. International Journal of Oral and Maxillofacial Implants. 2011;26(3):618.

Andruškevičienė V, Milčiuvienė S, Bendoraitienė E, Saldūnaitė K, Vasiliauskienė I, Slabšinskienė E, et al. Oral health status and effectiveness of caries prevention programme in kindergartens in Kaunas city (Lithuania). Oral health and preventive dentistry. 2008;6(4).

Nagasawa T, et al. Effects of single‐visit full‐mouth ultrasonic debridement versus quadrant‐wise ultrasonic debridement. Journal of Clinical Periodontology. 2005;32(7):734-43.

Polat O, Karaman AI. Pain control during fixed orthodontic appliance therapy. The Angle Orthodontist. 2005;75(2):214-9.

You BJ, Jian WW, Sheng RW, Jun Q, Wa WC, Bartizek RD, et al. Caries prevention in Chinese children with sodium fluoride dentifrice delivered through a kindergarten-based oral health program in China. The Journal of Clinical Dentistry. 2002;13(4):179-84.

Braun A, Dehn C, Krause F, Jepsen S. Short‐term clinical effects of adjunctive antimicrobial photodynamic therapy in periodontal treatment: a randomized clinical trial. Journal of Clinical Periodontology. 2008;35(10):877-84.

Spinato S, Grandi T, Prini M. A comparative study between different techniques in non-surgical periodontal treatment. Minerva stomatologica. 2006;55(5):289-96.

Pieri F, Aldini NN, Marchetti C, Corinaldesi G. Influence of implant-abutment interface design on bone and soft tissue levels around immediately placed and restored single-tooth implants: a randomized controlled clinical trial. International Journal of Oral and Maxillofacial Implants. 2011;26(1).

Chen L, Luo G, Xuan D, Wei B, Liu F, Li J, et al. Effects of non-surgical periodontal treatment on clinical response, serum inflammatory parameters, and metabolic control in patients with type 2 diabetes: a randomized study. Journal of Periodontology. 2012;83(4):435-43.

Naylor MN, Glass RL. A 3-year clinical trial of calcium carbonate dentifrice containing calcium glycerophosphate and sodium monofluorophosphate. Caries Research. 1979;13(1):39-46.

Crockett DJ, Foreman ME, Alden L, Blasberg B. A comparison of treatment modes in the management of myofascial pain dysfunction syndrome. Applied Psychophysiology and Biofeedback. 1986;11(4):279-91.

Mabry TW, Yukna RA, Sepe WW. Freeze-dried bone allografts combined with tetracycline in the treatment of juvenile periodontitis. Journal of Periodontology. 1985;56(2):74-81.

Silverman G, Berman E, Hanna CB, Salvato A, Fratarcangelo P, Bartizek RD, et al. Assessing the efficacy of three dentifrices in the treatment of dentinal hypersensitivity. The Journal of the American Dental Association. 1996;127(2):191-201.

Mengel R, Soffner M, Flores-de-Jacoby L. Bioabsorbable membrane and bioactive glass in the treatment of intrabony defects in patients with generalized aggressive periodontitis: results of a 12-month clinical and radiological study. Journal of Periodontology. 2003;74(6):899-908.

Brown GD, Mealey BL, Nummikoski PV, Bifano SL, Waldrop TC. Hydroxyapatite cement implant for regeneration of periodontal osseous defects in humans. Journal of Periodontology. 1998;69(2):146-57.

Pamir T, Dalgar H, Onal B. Clinical evaluation of three desensitizing agents in relieving dentin hypersensitivity. Operative dentistry. 2007;32(6):544-8.

Petersson LG, Svanholm I, Andersson H, Magnusson K. Approximal caries development following intensive fluoride mouthrinsing in teenagers. A 3-year radiographic study. European Journal of Oral Sciences. 1998;106(6):1048-51.

Silvestri M, Ricci G, Rasperini G, Sartori S, Cattaneo V. Comparison of treatments of infrabony defects with enamel matrix derivative, guided tissue regeneration with a nonresorbable membrane and Widman modified flap. Journal of Clinical Periodontology. 2000;27(8):603-10.

Shahdad SA, Taylor C, Barclay SC, Steen IN, Preshaw PM. A double‐blind, crossover study of Biotène Oralbalance and bioxtra systems as salivary substitutes in patients with post‐radiotherapy xerostomia. European Journal of Cancer Care. 2005;14(4):319-26.

Tonetti MS, Lang NP, Cortellini P, Suvan JE, Adriaens P, Dubravec D, et al. Enamel matrix proteins in the regenerative therapy of deep intrabony defects. Journal of Clinical Periodontology. 2002;29(4):317-25.

Curtis P, Gartman LA, Green DB. Utilization of ketorolac tromethamine for control of severe odontogenic pain. Journal of Endodontics. 1994;20(9):457-9.

Al Quran FAM, Kamal MS. Anterior midline point stop device (AMPS) in the treatment of myogenous tmds: comparison with the stabilization splint and control group. Oral Surgery, Oral Medicine, Oral Pathology, Oral Radiology, and Endodontology. 2006;101(6):741-7.

Gillam DG, Newman HN, Davies EH, Bulman JS, Troullos ES, Curro FA. Clinical evaluation of ferric oxalate in relieving dentine hypersensitivity. Journal of Oral Rehabilitation. 2004;31(3):245-50.

Marthaler TM, Knig KG, Mhlemann HR. The effect of a fluoride gel used for supervised toothbrushing 15 or 30 times per year. Helvetica Odontologica Acta. 1970;14(2):67-77.

Park J-S, Suh J-J, Choi S-H, Moon I-S, Cho K-S, Kim C-K, et al. Effects of pretreatment clinical parameters on bioactive glass implantation in intrabony periodontal defects. Journal of Periodontology. 2001;72(6):730-40.

Lekovic V, Kenney EB, Weinlaender M, Han T, Klokkevold P, Nedic M, et al. A bone regenerative approach to alveolar ridge maintenance following tooth extraction. Report of 10 cases. Journal of Periodontology. 1997;68(6):563-70.

Zacherl WA, mcphail CW. Final report on the efficacy of a stannous fluoride-calcium pyrophosphate dentifrice. Journal of the Canadian Dental Association. 1970;36(7):262.

Mcconchie JM, Richardson AS, Hole LW, mccombie F, Kolthammer J. Caries‐preventive effect of two concentrations of stannous fluoride mouthrinse. Community Dentistry and Oral Epidemiology. 1977;5(6):278-83.

Crespi R, Cappar P, Gherlone E. Radiographic evaluation of marginal bone levels around platform-switched and non-platform-switched implants used in an immediate loading protocol. International Journal of Oral and Maxillofacial Implants. 2009;24(5).

Sicilia A, Cuesta‐Frechoso S, Suárez A, Angulo J, Pordomingo A, De Juan P. Immediate efficacy of diode laser application in the treatment of dentine hypersensitivity in periodontal maintenance patients: a randomized clinical trial. Journal of Clinical Periodontology. 2009;36(8):650-60.

Morris MF, Davis RD, Richardson BW. Clinical efficacy of two dentin desensitizing agents. American Journal of Dentistry. 1999;12(2):72-6.

Clark DC, Stamm JW, Quee TC, Robert G. Results of the Sherbrooke‐Lac Megantic fluoride varnish study after 20 months. Community Dentistry and Oral Epidemiology. 1985;13(2):61-4.

Pamir T, Özyazici M, Baloğlu E, Önal B. The efficacy of three desensitizing agents in treatment of dentine hypersensitivity. Journal of Clinical Pharmacy and Therapeutics. 2005;30(1):73-6.

Mouly SJ, Orler J-B, Tillet Y, Coudert A-C, Oberli F, Preshaw P, et al. Efficacy of a new oral lubricant solution in the management of psychotropic drug-induced xerostomia: a randomized controlled trial. Journal of clinical psychopharmacology. 2007;27(5):437-43.

Ferguson KA, Ono T, Lowe AA, Al-Majed S, Love LL, Fleetham JA. A short-term controlled trial of an adjustable oral appliance for the treatment of mild to moderate obstructive sleep apnoea. Thorax. 1997;52(4):362-8.

Tarannum F, Faizuddin M. Effect of periodontal therapy on pregnancy outcome in women affected by periodontitis. Journal of Periodontology. 2007;78(11):2095-103.

Moeintaghavi A, Arab HR, Bozorgnia Y, Kianoush K, Alizadeh M. Non‐surgical periodontal therapy affects metabolic control in diabetics: a randomized controlled clinical trial. Australian Dental Journal. 2012;57(1):31-7.

Friberg B, Jisander S, Widmark G, Lundgren A, Ivanoff CJ, Sennerby L, et al. One‐Year Prospective Three‐Center Study Comparing the Outcome of a Soft Bone Implant (Prototype Mk IV) and the Standard Brånemark Implant. Clinical Implant Dentistry and Related Research. 2003;5(2):71-7.

Tonetti MS, D'Aiuto F, Nibali L, Donald A, Storry C, Parkar M, et al. Treatment of periodontitis and endothelial function. New England Journal of Medicine. 2007;356(9):911-20.

Pizzo G, Guiglia R, Imburgia M, Pizzo I, D'Angelo M, Giuliana G. The effects of antimicrobial sprays and mouthrinses on supragingival plaque regrowth: a comparative study. Journal of Periodontology. 2006;77(2):248-56.

Garcia-Godoy F, devizio W, Volpe AR, Ferlauto RJ, Miller JM. Effect of a triclosan/copolymer/fluoride dentifrice on plaque formation and gingivitis: a 7-month clinical study. American Journal of Dentistry. 1990;3:15.

Lalla RV, Choquette LE, Feinn RS, Zawistowski H, Latortue MC, Kelly ET, et al. Multivitamin therapy for recurrent aphthous stomatitis: a randomized, double-masked, placebo-controlled trial. The Journal of the American Dental Association. 2012;143(4):370-6.

De Jongh AD, Muris P, Ter Horst G, Van Zuuren F, Schoenmakers N, Makkes P. One-session cognitive treatment of dental phobia: preparing dental phobics for treatment by restructuring negative cognitions. Behaviour research and therapy. 1995;33(8):947-54.

Bensadoun RJ, Franquin JC, Ciais G, Darcourt V, Schubert MM, Viot M, et al. Low-energy He/Ne laser in the prevention of radiation-induced mucositis. Supportive care in cancer. 1999;7(4):244-52.

Modica F, Pizzo MD, Roccuzzo M, Romagnoli R. Coronally advanced flap for the treatment of buccal gingival recessions with and without enamel matrix derivative. A split-mouth study. Journal of Periodontology. 2000;71(11):1693-8.

Lekovic V, Camargo PM, Weinlaender M, Kenney EB, Vasilic N. Combination use of bovine porous bone mineral, enamel matrix proteins, and a bioabsorbable membrane in intrabony periodontal defects in humans. Journal of Periodontology. 2001;72(5):583-9.

Mellonig JT. Decalcified freeze-dried bone allograft as an implant material in human periodontal defects. The International Journal of Periodontics and Restorative Dentistry. 1984;4(6):40.

Camargo PM, Lekovic V, Weinlaender M, Nedic M, Vasilic N, Wolinsky LE, et al. A controlled re‐entry study on the effectiveness of bovine porous bone mineral used in combination with a collagen membrane of porcine origin in the treatment of intrabony defects in humans. Journal of Clinical Periodontology. 2000;27(12):889-96.

Scannapieco FA, Yu J, Raghavendran K, Vacanti A, Owens SI, Wood K, et al. A randomized trial of chlorhexidine gluconate on oral bacterial pathogens in mechanically ventilated patients. Critical Care. 2009;13(4):R117.

Sisk AL, Bonnington GJ. Evaluation of methylprednisolone and flurbiprofen for inhibition of the postoperative inflammatory response. Oral Surgery, Oral Medicine, Oral Pathology. 1985;60(2):137-45.

Marthaler TM. Caries-inhibition by an amine fluoride dentifrice results after 6 years in children with low caries activity. Helvetica Odontologica Acta. 1974;18:44.

Triol CW, Kranz SM, Volpe AR, Frankl SN, Alman JE, Allard RL. Anticaries effect of a sodium fluoride rinse and an MFP dentifrice in a nonfluoridated water area: A thirty-month study. Clinical Preventive Dentistry. 1980;2(2):13-5.

Adebola RA. Comparative study of effect of single and multiple suture techniques on inflammatory complications after third molar surgery. Journal of Oral and Maxillofacial Surgery. 2011;69(4):971-6.

Pritlove-Carson S, Palmer RM, Floyd PD. Evaluation of guided tissue regeneration in the treatment of paired periodontal defects. British Dental Journal. 1995;179(10):388-94.

Lekovic V, Camargo PM, Weinlaender M, Aleksic Z, Barrie Kenney E, Nedic M. A comparison between enamel matrix proteins used alone or in combination with bovine porous bone mineral in the treatment of intrabony periodontal defects in humans. Journal of Periodontology. 2000;71(7):1110-6.

Conti AJ, Lotzkar S, Daley R, Cancro L, Marks RG, mcneal DR. A 3‐year clinical trial to compare efficacy of dentifrices containing 1.14% and 0.76% sodium monofluorophosphate. Community Dentistry and Oral Epidemiology. 1988;16(3):135-8.

Finn SB, Moller P, Jamison H, Regattieri L, Manson-Hing L. The clinical cariostatic effectiveness of two concentrations of acidulated phosphate-fluoride mouthwash. The Journal of the American Dental Association. 1975;90(2):398-402.

Zacherl WA. Clinical evaluation of neutral sodium fluoride, stannous fluoride, sodium monofluorophosphate and acidulated fluoride-phosphate denifrices. Journal of the Canadian Dental Association. 1972;38(1):35-8.

Christgau M, Moder D, Wagner J, Glässl M, Hiller KA, Wenzel A, et al. Influence of autologous platelet concentrate on healing in intra‐bony defects following guided tissue regeneration therapy: a randomized prospective clinical split‐mouth study. Journal of Clinical Periodontology. 2006;33(12):908-21.

Reed MW, King JD. A clinical evaluation of a sodium fluoride dentifrice. Pharmacology and therapeutics in dentistry. 1975;2(2):77-82.

Pinho mdn, Oliveira RDR, Novaes Jr AB, Voltarelli JC. Relationship between periodontitis and rheumatoid arthritis and the effect of non-surgical periodontal treatment. Brazilian Dental Journal. 2009;20(5):355-64.

Guerrero A, Griffiths GS, Nibali L, Suvan J, Moles DR, Laurell L, et al. Adjunctive benefits of systemic amoxicillin and metronidazole in non‐surgical treatment of generalized aggressive periodontitis: a randomized placebo‐controlled clinical trial. Journal of Clinical Periodontology. 2005;32(10):1096-107.

Penniston SG, Hargreaves KM. Evaluation of periapical injection of Ketorolac for management of endodontic pain. Journal of Endodontics. 1996;22(2):55-9.

Payne AGT, Tawse‐Smith A, Thompson WM, Kumara R. Early functional loading of unsplinted roughened surface implants with mandibular overdentures 2 weeks after surgery. Clinical Implant Dentistry and Related Research. 2003;5(3):143-53.

Bokhari H, Syed A, Khan AA, Butt AK, Azhar M, Hanif M, et al. Non‐surgical periodontal therapy reduces coronary heart disease risk markers: a randomized controlled trial. Journal of Clinical Periodontology. 2012;39(11):1065-74.

Paolantonio M. Treatment of gingival recessions by combined periodontal regenerative technique, guided tissue regeneration, and subpedicle connective tissue graft. A comparative clinical study. Journal of Periodontology. 2002;73(1):53-62.

Lobene RR, Soparkar PM, Newman MB. Use of dental floss. Effect on plaque and gingivitis. Clinical Preventive Dentistry. 1982;4(1):5.

Bravo M, Baca P, Llodra JC, Osorio E. A 24‐month Study Comparing Sealant and Fluoride Varnish in Caries Reduction on Different Permanent First Molar Surfaces. Journal of Public Health Dentistry. 1997;57(3):184-6.

Campos GN, Pimentel SP, Ribeiro FV, Casarin RCV, Cirano FR, Saraceni CHC, et al. The adjunctive effect of photodynamic therapy for residual pockets in single-rooted teeth: a randomized controlled clinical trial. Lasers in Medical Science. 2013:1-8.

Schiff T, Dos Santos M, Laffi S, Yoshioka M, Baines E, Brasil KD, et al. Efficacy of a dentifrice containing 5% potassium nitrate and 1500 PPM sodium monofluorophosphate in a precipitated calcium carbonate base on dentinal hypersensitivity. The Journal of Clinical Dentistry. 1997;9(1):22-5.

Reed MW. Clinical evaluation of three concentrations of sodium fluoride in dentifrices. The Journal of the American Dental Association. 1973;87(7):1401-3.

Englander HR, Sherrill LT, Miller BG, Carlos JP, Mellberg JR, Senning RS. Incremental rates of dental caries after repeated topical sodium fluoride applications in children with lifelong consumption of fluoridated water. The Journal of the American Dental Association. 1971;82(2):354-8.

Blinkhorn AS, Holloway PJ, Davies TGH. Combined effects of a fluoride dentifrice and mouthrinse on the incidence of dental caries. Community Dentistry and Oral Epidemiology. 1983;11(1):7-11.

Bastos jrdm, Viegas AR, Lopes ES. Comparao entre o uso de solues de fluoreto de sdio a 0, 2%, monofluorfosfato de sdio a 0, 7% e monofluorfosfato de sdio a 0, 7% em alcool a 4% em bochechos semanais, na preveno da crie dentria: resultados de 12 meses. Revassocpaulcirdent. 1981;35(5):5, passim.

Guida L, Annunziata M, Belardo S, Farina R, Scabbia A, Trombelli L. Effect of autogenous cortical bone particulate in conjunction with enamel matrix derivative in the treatment of periodontal intraosseous defects. Journal of Periodontology. 2007;78(2):231-8.

Keijser JAM, Verkade H, Timmerman MF, Van der Weijden FA. Comparison of 2 commercially available chlorhexidine mouthrinses. Journal of Periodontology. 2003;74(2):214-8.

Chung KM, Salkin LM, Stein MD, Freedman AL. Clinical evaluation of a biodegradable collagen membrane in guided tissue regeneration. Journal of Periodontology. 1990;61(12):732-6.

Rugg-Gunn AJ, Holloway PJ, Davies TGH. Caries prevention by daily fluoride mouthrinsing. Report of a three-year clinical trial. British Dental Journal. 1973;135(8):353-60.

Boerrigter EM, Geertman ME, Van Oort RP, Bouma J, Raghoebar GM, Van Waas MAJ, et al. Patient satisfaction with implant-retained mandibular overdentures. A comparison with new complete dentures not retained by implants—a multicentre randomized clinical trial. British Journal of Oral and Maxillofacial Surgery. 1995;33(5):282-8.

Ripa LW, Leske GS, Sposato A, Varma A. Clinical comparison of the caries inhibition of two mixed naf-Na2PO3F dentifrices containing 1,000 and 2,500 ppm F compared to a conventional Na2PO3F dentifrice containing 1,000 ppm F: results after two years. Caries Research. 1987;21(2):149-57.

Kakaboura A, Rahiotis C, Thomaidis S, Doukoudakis S. Clinical effectiveness of two agents on the treatment of tooth cervical hypersensitivity. American Journal of Dentistry. 2005;18(4):291-5.

Pasqualini D, Cocero N, Castella A, Mela L, Bracco P. Primary and secondary closure of the surgical wound after removal of impacted mandibular third molars: a comparative study. International Journal of Oral and Maxillofacial Surgery. 2005;34(1):52-7.

Camargo PM. Root coverage with a coronally positioned flap used in combination with enamel matrix derivative: 18-month clinical evaluation. Journal of Periodontology. 2006;77(12):2031-9.

Leknes KN, Andersen K-M, Be OE, Skavland RJ, Albandar JM. Enamel matrix derivative versus bioactive ceramic filler in the treatment of intrabony defects: 12-month results. Journal of Periodontology. 2009;80(2):219-27.

Froum SJ, Ortiz M, Witkin RT, Thaler R, Scopp IW, Stahl SS. Osseous Autografts: III. Comparison of Osseous Coagulum-Bone Blend Implants with Open Curettage. Journal of Periodontology. 1976;47(5):287-94.

Yek EC, Cintan S, Topcuoglu N, Kulekci G, Issever H, Kantarci A. Efficacy of amoxicillin and metronidazole combination for the management of generalized aggressive periodontitis. Journal of Periodontology. 2010;81(7):964-74.

Carlson CR, Bertrand PM, Ehrlich AD, Maxwell AW, Burton RG. Physical self-regulation training for the management of temporomandibular disorders. Journal of Orofacial Pain. 2001;15(1).

Silverstein L, Bissada N, Manouchehr-Pour M, Greenwell H. Clinical and microbiologic effects of local tetracycline irrigation on periodontitis. Journal of Periodontology. 1988;59(5):301-5.

Dos Anjos B, Novaes Jr AB, Meffert R, Barboza EP. Clinical comparison of cellulose and expanded polytetrafluoroethylene membranes in the treatment of class II furcations in mandibular molars with 6-month re-entry. Journal of Periodontology. 1998;69(4):454-9.

Subbaiah R, Thomas B. Efficacy of a bioactive alloplast, in the treatment of human periodontal osseous defects-a clinical study. Medicina Oral Patologia Oral y Cirugia Bucal. 2011;16(2):e244.

Hoang T, Jorgensen MG, Keim RG, Pattison AM, Slots J. Povidone‐iodine as a periodontal pocket disinfectant. Journal of Periodontal Research. 2003;38(3):311-7.

Sun W-L, Chen L-L, Zhang S-Z, Ren Y-Z, Qin G-M. Changes of adiponectin and inflammatory cytokines after periodontal intervention in type 2 diabetes patients with periodontitis. Archives of Oral Biology. 2010;55(12):970-4.

Stephen KW, Chestnutt IG, Jacobson AP, mccall DR, Chesters RK, Huntington E, et al. The effect of naf and SMFP toothpastes on three-year caries increments in adolescents. International Dental Journal. 1994;44(3 Suppl 1):287-95.

Maze GI, Reinhardt RA, Agarwal RK, Dyer JK, Robinson DH, dubois LM, et al. Response to intracrevicular controlled delivery of 25% tetracycline from poly (lactide/glycolide) film strips in SPT patients. Journal of Clinical Periodontology. 1995;22(11):860-7.

Shapira L, Shapira M, Tandlich M, Gedalia I. Effect of amine fluoride-stannous fluoride containing toothpaste (Meridol) on plaque and gingivitis in adults: a six-month clinical study. Journal of the International Academy of Periodontology. 1999;1(4):117-20.

Modéer T, Twetman S, Bergstrand F. Three‐year study of the effect of fluoride varnish (Duraphat) on proximal caries progression in teenagers. European Journal of Oral Sciences. 1984;92(5):400-7.

Özçaka Ö, Başoğlu ÖK, Buduneli N, Taşbakan MS, Bacakoğlu F, Kinane DF. Chlorhexidine decreases the risk of ventilator‐associated pneumonia in intensive care unit patients: a randomized clinical trial. Journal of Periodontal Research. 2012;47(5):584-92.

Meadows CL, Gher ME, Quintero G, Lafferty TA. A comparison of polylactic acid granules and decalcified freeze-dried bone allograft in human periodontal osseous defects. Journal of Periodontology. 1993;64(2):103-9.

Kuhn A, Porto FA, Miraglia P, Brunetto AL. Low-level infrared laser therapy in chemotherapy-induced oral mucositis: a randomized placebo-controlled trial in children. Journal of pediatric hematology/oncology. 2009;31(1):33-7.

Skjelbred P, Lkken P. Post-operative pain and inflammatory reaction reduced by injection of a corticosteroid. European Journal of Clinical Pharmacology. 1982;21(5):391-6.

Offenbacher S, Beck JD, Jared HL, Mauriello SM, Mendoza LC, Couper DJ, et al. Effects of periodontal therapy on rate of preterm delivery a randomized controlled trial. Obstetrics and Gynecology. 2009;114(3):551.

Bauroth K, Charles CH, Mankodi SM, Simmons K, Zhao Q, Kumar LD. The efficacy of an essential oil antiseptic mouthrinse vs. Dental floss in controlling interproximal gingivitis: a comparative study. The Journal of the American Dental Association. 2003;134(3):359-65.

Craig EW, Suckling GW, Pearce EI. The effect of a preventive programme on dental plaque and caries in school children. The New Zealand dental journal. 1981;77(349):89.

Marthaler TM. The caries-inhibiting effect of amine fluoride dentifrices in children during three years of unsupervised use. British Dental Journal. 1965;119:153.

Sculean A, Windisch P, Chiantella GC, Donos N, Brecx M, Reich E. Treatment of intrabony defects with enamel matrix proteins and guided tissue regeneration. Journal of Clinical Periodontology. 2001;28(5):397-403.

West NX, Hughes JA, Parker D, Weaver LJ, Moohan M, De'Ath J, et al. Modification of soft drinks with xanthan gum to minimise erosion: a study in situ. British Dental Journal. 2004;196(8):478-81.

Salmassian R, Oesterle LJ, Shellhart WC, Newman SM. Comparison of the efficacy of ibuprofen and acetaminophen in controlling pain after orthodontic tooth movement. American Journal of Orthodontics and Dentofacial Orthopedics. 2009;135(4):516-21.

Sowinski JA, Battista GW, Petrone ME, Chaknis P, Zhang YP, devizio W, et al. A new desensitizing dentifrice--an 8-week clinical investigation. Compendium of Continuing Education in Dentistry (Jamesburg, NJ: 1995)Supplement. 1999(27):6; quiz 28.

Crespi R, Cappar P, Gherlone E. Magnesium-enriched hydroxyapatite compared to calcium sulfate in the healing of human extraction sockets: radiographic and histomorphometric evaluation at 3 months. Journal of Periodontology. 2009;80(2):210-8.

Overholser CD, Meiller TF, depaola LG, Minah GE, Niehaus C. Comparative effects of 2 mouthrinses on the development of supragingival dental plaque and gingivitis. Journal of Clinical Periodontology. 1990;17(8):575-9.

Muhler JC, Radike AW, Nebergall WH, Day HG. A comparison between the anticariogenic effects of dentifrices containing stannous fluoride and sodium fluoride. The Journal of the American Dental Association. 1955;51(5):556-9.

Chesters RK, Pitts NB, Matuliene G, Kvedariene A, Huntington E, Bendinskaite R, et al. An abbreviated caries clinical trial design validated over 24 months. Journal of Dental Research. 2002;81(9):637-40.

Segal AH, Stiff RH, George WA, Picozzi A. Cariostatic effect of a stannous fluoride-containing dentifrice on children: two-year report of a supervised toothbrushing study. Journal of oral therapeutics and pharmacology. 1967;4(3):175.

Chondros P, Nikolidakis D, Christodoulides N, Rssler R, Gutknecht N, Sculean A. Photodynamic therapy as adjunct to non-surgical periodontal treatment in patients on periodontal maintenance: a randomized controlled clinical trial. Lasers in medical science. 2009;24(5):681-8.

Anderson RJ, Beal JF, Bradnock G. A 3‐year clinical trial of the effect on dental caries of a dentifrice containing 2% sodium monofluorophosphate. Community Dentistry and Oral Epidemiology. 1977;5(2):67-72.

Willumsen T, Vassend O, Hoffart A. A comparison of cognitive therapy, applied relaxation, and nitrous oxide sedation in the treatment of dental fear. Acta Odontologica Scandinavica. 2001;59(5):290-6.

Mergele M. Report II. An unsupervised brushing study on subjects residing in a community with fluoride in the water. Acad Med NJ Bull. 1968;14:251-5.

Purton DG, Torr B, Tn NHDT. A randomized controlled clinical trial of conventional and immediately loaded tapered implants with screw-retained crowns. The International Journal of Prosthodontics. 2006;19:17-9.

Volkov I, Rudoy I, Freud T, Sardal G, Naimer S, Peleg R, et al. Effectiveness of vitamin B12 in treating recurrent aphthous stomatitis: a randomized, double-blind, placebo-controlled trial. The Journal of the American Board of Family Medicine. 2009;22(1):9-16.

Gerlach RW, Barker ML, Sagel PA. Comparative efficacy and tolerability of two direct-to-consumer tooth whitening systems. American Journal of Dentistry. 2001;14(5):267-72.

Slack GL, Bulman JS, Osborn JF. Clinical testing of fluoride and non-fluoride containing dentifrices in Hounslow school children. British Dental Journal. 1971;130(4):154-8.

Yukna CN, Yukna RA. Multi-center evaluation of bioabsorbable collagen membrane for guided tissue regeneration in human Class II furcations. Journal of Periodontology. 1996;67(7):650-7.

Fourrier F, Cau-Pottier E, Boutigny H, Roussel-Delvallez M, Jourdain M, Chopin C. Effects of dental plaque antiseptic decontamination on bacterial colonization and nosocomial infections in critically ill patients. Intensive care medicine. 2000;26(9):1239-47.

Aimetti M, Romano F, Griga FB, Godio L. Clinical and histologic healing of human extraction sockets filled with calcium sulfate. International Journal of Oral and Maxillofacial Implants. 2009;24(5).

Neupert EA, Lee JW, Philput CB, Gordon JR. Evaluation of dexamethasone for reduction of postsurgical sequelae of third molar removal. Journal of Oral and Maxillofacial Surgery. 1992;50(11):1177-82.

Dilsiz A, Canakci V, Aydin T. Clinical effects of potassium–titanyl–phosphate laser and photodynamic therapy on outcomes of treatment of chronic periodontitis: a randomized controlled clinical trial. Journal of Periodontology. 2013;84(3):278-86.

Jepsen S, Topoll H, Rengers H, Heinz B, Teich M, Hoffmann T, et al. Clinical outcomes after treatment of intra‐bony defects with an EMD/synthetic bone graft or EMD alone: a multicentre randomized‐controlled clinical trial. Journal of Clinical Periodontology. 2008;35(5):420-8.

Singh S, Kumar V, Kumar S, Subbappa A. The effect of periodontal therapy on the improvement of glycemic control in patients with type 2 diabetes mellitus: A randomized controlled clinical trial. International Journal of Diabetes in Developing Countries. 2008;28(2):38.

Triratana T, Kraivaphan P, Amornchat C, Rustogi K, Petrone MP, Volpe AR. Effect of a triclosan/copolymer pre-brush mouthrinse on established plaque formation and gingivitis: a six-month clinical study in Thailand. The Journal of Clinical Dentistry. 1994;6(2):142-7.

Williams C, mcbride S, Bolden TE, Mostler K, Petrone DM, Petrone ME, et al. Clinical efficacy of an optimized stannous fluoride dentifrice, Part 3: A 6-month plaque/gingivitis clinical study, southeast USA. Compendium of Continuing Education in Dentistry (Jamesburg, NJ: 1995). 1996;18:16-20.

Al‐Arrayed F, Adam S, Moran J, Dowell P. Clinical trial of cross‐linked human type I collagen as a barrier material in surgical periodontal treatment. Journal of Clinical Periodontology. 1995;22(5):371-9.

Law SLS, Southard KA, Law AS, Logan HL, Jakobsen JR. An evaluation of preoperative ibuprofen for treatment of pain associated with orthodontic separator placement. American Journal of Orthodontics and Dentofacial Orthopedics. 2000;118(6):629-35.

Peterson JK. A supervised brushing trial of sodium monofluorophosphate dentifrices in a fluoridated area. Caries Research. 1979;13(2):68-72.

Refo'a Y, Ouatik N, Golchin F, Mahboobi N. Comparing primary and secondary wound healing discomfort after mandibular third molar surgery: a randomized, double-blind clinical trial. General dentistry. 2010;59(4):310-3.

Kenney EB, Lekovic V, Han T, Carranza Jr FA, Dimitrijevic B. The Use of a Porous Hydroxylapatite Implant in Periodontal Defects I. Clinical Results after Six Months. Journal of Periodontology. 1985;56(2):82-8.

Mayfield L, Sderholm G, Hallstrm H, Kullendorff B, Edwardsson S, Bratthall G, et al. Guided tissue regeneration for the treatment of intraosseous defects using a biabsorbable membrane a controlled clinical study. Journal of Clinical Periodontology. 1998;25(7):585-95.

Bello SA, Olaitan AA, Ladeinde AL. A randomized comparison of the effect of partial and total wound closure techniques on postoperative morbidity after mandibular third molar surgery. Journal of Oral and Maxillofacial Surgery. 2011;69(6):e30.

Cahen PM, Frank RM, Turlot JC, Juno MT. Comparative unsupervised clinical trial on caries inhibition effect of monofluorophosphate and amine fluoride dentifrices after 3 years in Strasbourg, France. Community Dentistry and Oral Epidemiology. 1982;10(5):238-41.

Triratana T, Rustogi KN, Volpe AR, devizio W, Petrone M, Giniger M. Clinical effect of a new liquid dentifrice containing triclosan/copolymer on existing plaque and gingivitis. The Journal of the American Dental Association. 2002;133(2):219-25.

Romagna-Genon C. Comparative clinical study of guided tissue regeneration with a bioabsorbable bilayer collagen membrane and subepithelial connective tissue graft. Journal of Periodontology. 2001;72(9):1258-64.

Trombelli L, Simonelli A, Pramstraller M, Wikesj UME, Farina R. Single flap approach with and without guided tissue regeneration and a hydroxyapatite biomaterial in the management of intraosseous periodontal defects. Journal of Periodontology. 2010;81(9):1256-63.

Hague AL, Carr MP. Efficacy of an automated flossing device in different regions of the mouth. Journal of Periodontology. 2007;78(8):1529-37.

Ozan O, Turkyilmaz I, Yilmaz B. A preliminary report of patients treated with early loaded implants using computerized tomography‐guided surgical stents: flapless versus conventional flapped surgery. Journal of Oral Rehabilitation. 2007;34(11):835-40.

Michalowicz BS, Hodges JS, diangelis AJ, Lupo VR, Novak MJ, Ferguson JE, et al. Treatment of periodontal disease and the risk of preterm birth. New England Journal of Medicine. 2006;355(18):1885-94.

Singh VP, Nayak DG, Uppoor AS, Shah D. Nano-crystalline hydroxyapatite bone graft combined with bioresorbable collagen membrane in the treatment of periodontal intrabony defects: a randomized controlled clinical trial. Journal of Indian Society of Periodontology. 2012;16(4):562.

Mankodi S, Bauroth K, Witt JJ, Bsoul S, He T, Gibb R, et al. 6-month clinical trial to study the effects of a cetylpyridinium chloride mouthrinse on gingivitis and plaque. American Journal of Dentistry . 2005;18(Spec No).

Yukna RA, Evans GH, Aichelmann-Reidy MB, Mayer ET. Clinical comparison of bioactive glass bone replacement graft material and expanded polytetrafluoroethylene barrier membrane in treating human mandibular molar class II furcations. Journal of Periodontology. 2001;72(2):125-33.

Hooper SM, Newcombe RG, Faller R, Eversole S, Addy M, West NX. The protective effects of toothpaste against erosion by orange juice: studies in situ and in vitro. Journal of dentistry. 2007;35(6):476-81.

Mankodi S, Chaknis P, Panagakos FS, devizio W, Proskin HM. Comparative investigation of a dentifrice containing triclosan/copolymer/sodium fluoride and specially-designed silica and a dentifrice containing 0.243% sodium fluoride in a silica base for the control of established supra-gingival plaque and gingivitis: A 6-month clinical study. American Journal of Dentistry. 2011;24:21A.

Zanatta GM, Bittencourt S, Nociti Jr FH, Sallum EA, Sallum AW, Casati MZ. Periodontal debridement with povidone-iodine in periodontal treatment: short-term clinical and biochemical observations. Journal of Periodontology. 2006;77(3):498-505.

Kuhn A, Vacaro G, Almeida D, Machado l, Braghini PB, Shilling MA, et al. Low-level Infrared Laser Therapy for Chemo-or Radiotherapy-induced Oral Mucositis: A Randomized, Placebo-controlled Study. Journal of Oral Laser Applications. 2007;7(3).

Schincaglia GP, Marzola R, Scapoli C, Scotti R. Immediate loading of dental implants supporting fixed partial dentures in the posterior mandible: a randomized controlled split-mouth study--machined versus titanium oxide implant surface. International Journal of Oral and Maxillofacial Implants. 2007;22(1).

Vieira AHM, Passos VF, de Assis JS, Mendona JS, Santiago SL. Clinical evaluation of a 3% potassium oxalate gel and a gaalas laser for the treatment of dentinal hypersensitivity. Photomedicine and Laser Surgery. 2009;27(5):807-12.

Masters LB, Mellonig JT, Brunsvold MA, Nummikoski PV. A clinical evaluation of demineralized freeze-dried bone allograft in combination with tetracycline in the treatment of periodontal osseous defects. Journal of Periodontology. 1996;67(8):770-81.

Koch G, Bergmann-Arnadottir I, Bjarnason S, Finnbogason S, Hskuldsson O, Karlsson R. Caries-preventive effect of fluoride dentifrices with and without anticalculus agents: a 3-year controlled clinical trial. Caries Research. 1990;24(1):72-9.

Newnham JP, Newnham IA, Ball CM, Wright M, Pennell CE, Swain J, et al. Treatment of periodontal disease during pregnancy: a randomized controlled trial. Obstetrics and Gynecology. 2009;114(6):1239-48.

Kanchanakamol U, Umpriwan R, Jotikasthira N, Srisilapanan P, Tuongratanaphan S, Sholitkul W, et al. Reduction of plaque formation and gingivitis by a dentifrice containing triclosan and copolymer. Journal of Periodontology. 1995;66(2):109-12.

Torell P, Ericsson Y. Two-year clinical tests with different methods of local caries-preventive fluorine application in Swedish school-children. Acta Odontologica Scandinavica. 1965;23(3):287-322.

Rosenberg ES, Fox GK, Cohen C. Bioactive glass granules for regeneration of human periodontal defects. Journal of Esthetic and Restorative Dentistry. 2000;12(5):248-57.

Scott TA, Towle HJ, Assad DA, Nicoll BK. Comparison of bioabsorbable laminar bone membrane and non-resorbable eptfe membrane in mandibular furcations. Journal of Periodontology. 1997;68(7):679-86.

Randerath WJ, Heise M, Hinz R, Ruehle K-H. An individually adjustable oral appliance vs continuous positive airway pressure in mild-to-moderate obstructive sleep apnea syndrome. CHEST Journal. 2002;122(2):569-75.

Dri F, Huszar T, Nikolidakis D, Arweiler NB, Gera I, Sculean A. Effect of platelet-rich plasma on the healing of intrabony defects treated with an anorganic bovine bone mineral and expanded polytetrafluoroethylene membranes. Journal of Periodontology. 2007;78(6):983-90.

Ong MMA, Eber RM, Korsnes MI, macneil RL, Glickman GN, Shyr Y, et al. Evaluation of a bioactive glass alloplast in treating periodontal intrabony defects. Journal of Periodontology. 1998;69(12):1346-54.

Gerschman JA, Ruben J, Gebart‐Eaglemont J. Low level laser therapy for dentinal tooth hypersensitivity. Australian Dental Journal. 1994;39(6):353-7.

Szwejda LF. Fluorides in community programs; a study of four years of various fluorides applied topically to the teeth of children in fluoridated communities. Journal of Public Health Dentistry. 1972;32(1):25-33.

Ringelberg ML, Webster DB, Dixon DO, lezotte DC. The caries-preventive effect of amine fluorides and inorganic fluorides in a mouthrinse or dentifrice after 30 months of use. The Journal of the American Dental Association. 1979;98(2):202-8.

Camargo PM, Lekovic V, Weinlaender M, Vasilic N, Madzarevic M, Kenney EB. A reentry study on the use of bovine porous bone mineral, GTR, and platelet-rich plasma in the regenerative treatment of intrabony defects in humans. The International Journal of Periodontics and Restorative Dentistry. 2005;25(1):49-59.

Yukna RA, Callan DP, Krauser JT, Evans GH, Aichelmann-Reidy ME, Moore K, et al. Multi-center clinical evaluation of combination anorganic bovine-derived hydroxyapatite matrix (ABM)/cell binding peptide (P-15) as a bone replacement graft material in human periodontal osseous defects. 6-month results. Journal of Periodontology. 1998;69(6):655-63.

Grossman E, Dembling W, Proskin HM. A comparative clinical investigation of the safety and efficacy of an oscillating/rotating electric toothbrush and a sonic toothbrush. The Journal of Clinical Dentistry. 1994;6(1):108-12.

Fiorellini JP, Howell TH, Cochran D, Malmquist J, Lilly LC, Spagnoli D, et al. Randomized study evaluating recombinant human bone morphogenetic protein-2 for extraction socket augmentation. Journal of Periodontology. 2005;76(4):605-13.

Dilsiz A, Canakci V, Ozdemir A, Kaya Y. Clinical evaluation of Nd: YAG and 685-nm diode laser therapy for desensitization of teeth with gingival recession. Photomedicine and Laser Surgery. 2009;27(6):843-8.

Ngan P, Wilson S, Shanfeld J, Amini H. The effect of ibuprofen on the level of discomfort in patients undergoing orthodontic treatment. American Journal of Orthodontics and Dentofacial Orthopedics. 1994;106(1):88-95.

Mora F, Etienne D, Ouhayoun JP. Treatment of interproximal angular defects by guided tissue regeneration: 1 year follow‐up. Journal of Oral Rehabilitation. 1996;23(9):599-606.

Charles CH, Mostler KM, Bartels LL, Mankodi SM. Comparative antiplaque and antigingivitis effectiveness of a chlorhexidine and an essential oil mouthrinse: 6‐month clinical trial. Journal of Clinical Periodontology. 2004;31(10):878-84.

Thomas AE, Jamison HC. Effect of snf2 dentrifices on caries in children: Two-year clinical study of supervised brushing in children’s homes. The Journal of the American Dental Association. 1966;73(4):844-52.

Promsudthi A, Pimapansri S, Deerochanawong C, Kanchanavasita W. The effect of periodontal therapy on uncontrolled type 2 diabetes mellitus in older subjects. Oral diseases. 2005;11(5):293-8.

Schiff T, Zhang YP, devizio W, Stewart B, Chaknis P, Petrone ME, et al. A randomized clinical trial of the desensitizing efficacy of three dentifrices. Compendium of Continuing Education in Dentistry (Jamesburg, NJ: 1995)Supplement. 2000(27):10; quiz 28.

Van de Velde T, Sennerby L, De Bruyn H. The clinical and radiographic outcome of implants placed in the posterior maxilla with a guided flapless approach and immediately restored with a provisional rehabilitation: a randomized clinical trial. Clinical Oral Implants Research. 2010;21(11):1223-33.

Lobene RR. Evaluation of altered gingival health from permissive powered toothbrushing. The Journal of the American Dental Association. 1964;69(5):585-8.

Renvert S, Badersten A, Nilvus R, Egelberg J. Healing after treatment of periodontal intraosseous defects I. Comparative study of clinical methods. Journal of Clinical Periodontology. 1981;8(5):387-99.

Engleman HM, mcdonald JP, Graham D, Lello GE, Kingshott RN, Coleman EL, et al. Randomized crossover trial of two treatments for sleep apnea/hypopnea syndrome: continuous positive airway pressure and mandibular repositioning splint. American Journal of Respiratory and Critical Care Medicine. 2002;166(6):855-9.

Cortellini P, Carnevale G, Sanz M, Tonetti MS. Treatment of deep and shallow intrabony defects A multicenter randomized controlled clinical trial. Journal of Clinical Periodontology. 1998;25(12):981-7.

Astrand P, Engquist B, Dahlgren S, Engquist E, Feldmann H, Gröndahl K. Astra Tech and Brånemark System Implants: A Prospective 5‐Year Comparative Study. Results after One Year. Clinical Implant Dentistry and Related Research. 1999;1(1):17-26.

Mankodi S, Petrone DM, Battista G, Petrone ME, Chaknis P, devizio W, et al. Clinical efficacy of an optimized stannous fluoride dentifrice, Part 2: A 6-month plaque/gingivitis clinical study, northeast USA. Compendium of Continuing Education in Dentistry (Jamesburg, NJ: 1995). 1997;18:10-5.

Litt MD, Shafer DM, Kreutzer DL. Brief cognitive-behavioral treatment for TMD pain: Long-term outcomes and moderators of treatment. Pain. 2010;151(1):110-6.

Lo EM, Schwarz E, Wong MCM. Arresting dentine caries in Chinese preschool children. International Journal of Paediatric Dentistry.

Koeman M, van der Ven AJAM, Hak E, Joore HCA, Kaasjager K, de Smet AGA, et al. Oral decontamination with chlorhexidine reduces the incidence of ventilator-associated pneumonia. American journal of respiratory and critical care medicine. 2006;173(12):1348-55.

Mcclanahan SF, Beiswanger BB, Bartizek RD, Lanzalaco AC, Bacca L, White DJ. A comparison of stabilized stannous fluoride dentifrice and triclosan/copolymer dentifrice for efficacy in the reduction of gingivitis and gingival bleeding: six-month clinical results. The Journal of Clinical Dentistry. 1996;8(2 Spec No):39-45.

Erdoğan MF. The effect of improved periodontal health on metabolic control in type 2 diabetes mellitus. Journal of Clinical Periodontology. 2005;32(3):266-72.

Olivier M, Brodeur JM, Simard PL. Efficacy of APF treatments without prior tooth cleaning targeted to high‐risk children. Community Dentistry and Oral Epidemiology. 1992;20(1):38-42.

Buyukkurt MC, Gungormus M, Kaya O. The effect of a single dose prednisolone with and without diclofenac on pain, trismus, and swelling after removal of mandibular third molars. Journal of Oral and Maxillofacial Surgery. 2006;64(12):1761-6.

Hague AL, Carr MP, Rashid RG. Evaluation of the safety and efficacy of an automated flossing device: a randomized controlled trial. The Journal of Clinical Dentistry. 2007;18(2):45-8.

Ouyang X-Y, Qiao J. Effect of platelet-rich plasma in the treatment of periodontal intrabony defects in humans. Chinese medical journal. 2006;119(18):1511-21.

Horowitz HS, Doyle J. The effect on dental caries of topically applied acidulated phosphate-fluoride: results after three years. The Journal of the American Dental Association. 1971;82(2):359-65.

Stewart JE, Wager KA, Friedlander AH, Zadeh HH. The effect of periodontal treatment on glycemic control in patients with type 2 diabetes mellitus. Journal of Clinical Periodontology. 2001;28(4):306-10.

Allen DR, Battista GW, Petrone DM, Petrone ME, Chaknis P, devizio W, et al. The clinical efficacy of Colgate Total Plus Whitening Toothpaste containing a special grade of silica and Colgate Total Fresh Stripe Toothpaste in the control of plaque and gingivitis: a six-month clinical study. The Journal of Clinical Dentistry. 2002;13(2):59-64.

Osunde OD, Saheeb BD, Adebola RA. Comparative study of effect of single and multiple suture techniques on inflammatory complications after third molar surgery. Journal of Oral and Maxillofacial Surgery. 2011;69(4):971-6.

Dybvik T, Leknes KN, Be OE, Skavland RJ, Albandar JM. Bioactive ceramic filler in the treatment of severe osseous defects: 12-month results. Journal of Periodontology. 2007;78(3):403-10.

Kirkegaard E, Poulsen S, Bangsbo G, Bro K, editors. A clinical-trial of fluoride rinses in a danish public child dental service. Caries Research; 1984: ch-4009.

Paolantonio M, Perinetti G, Dolci M, Perfetti G, Tet S, Sammartino G, et al. Surgical treatment of periodontal intrabony defects with calcium sulfate implant and barrier versus collagen barrier or open flap debridement alone: a 12-month randomized controlled clinical trial. Journal of Periodontology. 2008;79(10):1886-93.

Fischer MJ, Reiners A, Kohnen R, Bernateck M, Gutenbrunner C, Fink M, et al. Do occlusal splints have an effect on complex regional pain syndrome? A randomized, controlled proof-of-concept trial. The Clinical journal of pain. 2008;24(9):776-83.

Heijl L, Heden G, Svrdstrm G, stgren A. Enamel matrix derivative. Journal of Clinical Periodontology. 1997;24:705-14.

Heifetz SB, Horowitz HS, Driscoll WS. Two‐year evaluation of a self‐administered procedure for the topical application of acidulated phosphate‐fluoride; final report. Journal of Public Health Dentistry. 1970;30(1):7-12.

Turk DC, Zaki HS, Rudy TE. Effects of intraoral appliance and biofeedback/stress management alone and in combination in treating pain and depression in patients with temporomandibular disorders. The Journal of Prosthetic Dentistry. 1993;70(2):158-64.

Cooley RL. Effectiveness of potassium oxalate treatment on dentin hypersensitivity. General Dentistry. 1989;37:330-3.

Del Peloso Ribeiro r, Bittencourt S, Ambrosano GMB, Nociti Jr FH, Sallum EA, Sallum AW, et al. Povidone-iodine used as an adjunct to non-surgical treatment of furcation involvements. Journal of Periodontology. 2006;77(2):211-7.

Ekstrand KR, Kuzmina IN, Kuzmina E, Christiansen MEC. Two and a Half–Year Outcome of Caries–Preventive Programs Offered to Groups of Children in the Solntsevsky District of Moscow. Caries Research. 2000;34(1):8-19.

Glass RL. A clinical study of hand and electric toothbrushing. Journal of Periodontology. 1965;36(4):322-7.

Biesbrock AR, Gerlach RW, Bollmer BW, Faller RV, Jacobs SA, Bartizek RD. Relative anti‐caries efficacy of 1100, 1700, 2200, and 2800 ppm fluoride ion in a sodium fluoride dentifrice over 1 year. Community Dentistry and Oral Epidemiology. 2001;29(5):382-9.

Kiliç AR, Efeoǧlu E, Yilmaz S. Guided tissue regeneration in conjunction with hydroxyapatite‐collagen grafts for intrabony defects. Journal of Clinical Periodontology. 1997;24(6):372-83.

Franco Neto CA, Parolo CCF, Rsing CK, Maltz M. Comparative analysis of the effect of two chlorhexidine mouthrinses on plaque accumulation and gingival bleeding. Brazilian oral research. 2008;22(2):139-44.

Flemmig TF, Ehmke B, Bolz K, Kbler NR, Karch H, Reuther JF, et al. Long-term maintenance of alveolar bone gain after implantation of autolyzed, antigen-extracted, allogenic bone in periodontal intraosseous defects. Journal of Periodontology. 1998;69(1):47-53.

Ashley FP, Naylor MN, Emslie RD. Stannous fluoride and sodium monofluorophosphate dentifrices. Clinical testing in London school children-radiological findings. British Dental Journal. 1969;127(3):125-8.

Tawse‐Smith A, Payne AGT, Kumara R, Thomson WM. One‐Stage Operative Procedure Using Two Different Implant Systems: A Prospective Study on Implant Overdentures in the Edentulous Mandible. Clinical Implant Dentistry and Related Research. 2001;3(4):185-93.

Ratka‐Krüger P, Neukranz E, Raetzke P. Guided tissue regeneration procedure with bioresorbable membranes versus conventional flap surgery in the treatment of infrabony periodontal defects. Journal of Clinical Periodontology. 2000;27(2):120-7.

Ho HP, Niederman R. Effectiveness of the Sonicare sonic toothbrush on reduction of plaque, gingivitis, probing pocket depth and subgingival bacteria in adolescent orthodontic patients. The Journal of Clinical Dentistry. 1997;8(1 Spec No):15-9.

Veerkamp JS, Gruythuysen RJ, Hoogstraten J, Van Amerongen WE. Dental treatment of fearful children using nitrous oxide. Part 4: Anxiety after two years. ASDC Journal of Dentistry for Children. 1993;60(4):372-6.

Johnson BD, mclnnes C. Clinical evaluation of the efficacy and safety of a new sonic toothbrush. Journal of Periodontology. 1994;65(7):692-7.

Wang H-L, Bunyaratavej P, Labadie M, Shyr Y, macneil RL. Comparison of 2 clinical techniques for treatment of gingival recession. Journal of Periodontology. 2001;72(10):1301-11.

Cowen D, Tardieu C, Schubert M, Peterson D, Resbeut M, Faucher C, et al. Low energy helium-neon laser in the prevention of oral mucositis in patients undergoing bone marrow transplant: results of a double blind randomized trial. International Journal of Radiation Oncology* Biology* Physics. 1997;38(4):697-703.

Denepitiya JL, Fine D, Singh S, devizio W, Volpe AR, Person P. Effect upon plaque formation and gingivitis of a triclosan/copolymer/fluoride dentifrice: a 6-month clinical study. American Journal of Dentistry. 1992;5(6):307-11.

Schincaglia GP, Marzola R, Giovanni GF, Chiara CS, Scotti R. Replacement of mandibular molars with single-unit restorations supported by wide-body implants: immediate versus delayed loading. A randomized controlled study. International Journal of Oral and Maxillofacial Implants. 2008;23(3).

Malferrari S. Desensitizing effects of Gluma and Gluma 2000 on hypersensitive dentin. American Journal of Dentistry. 1993;6(6):283-6.

Fourrier F, Dubois D, Pronnier P, Herbecq P, Leroy O, Desmettre T, et al. Effect of gingival and dental plaque antiseptic decontamination on nosocomial infections acquired in the intensive care unit: a double-blind placebo-controlled multicenter study. Critical Care Medicine. 2005;33(8):1728-35.

Schrad SC, Tussing GJ. Human allografts of iliac bone and marrow in periodontal osseous defects. Journal of Periodontology. 1986;57(4):205-10.

Gentile LC, Greghi SLA. Clinical evaluation of dentin hypersensitivity treatment with the low intensity Gallium-Aluminum-Arsenide laser-asgaal. Journal of Applied Oral Science. 2004;12(4):267-72.

Buhe H, Buttner W, Barlage B. Uber einen dreijahrigen klinischen zahncremetest mit zahnpasten unterschiedlicher fluoridkonzentration: 0.8% und 1.2% natriummonofluorophosphat. Quintessenz. 1984;35(1):103-11.

Tonetti MS, Cortellini P, Suvan JE, Adriaens P, Baldi C, Dubravec D, et al. Generalizability of the added benefits of guided tissue regeneration in the treatment of deep intrabony defects. Evaluation in a multi-center randomized controlled clinical trial. Journal of Periodontology. 1998;69(11):1183-92.

Baltacioglu E, Aslan M, Sara z, Saybak A, Yuva P. Analysis of clinical results of systemic antimicrobials combined with nonsurgical periodontal treatment for generalized aggressive periodontitis: a pilot study. Journal of the Canadian Dental Association. 2011;77.

Schiff T, Proskin HM, Zhang YP, Petrone M, devizio W. A clinical investigation of the efficacy of three different treatment regimens for the control of plaque and gingivitis. The Journal of Clinical Dentistry. 2006;17(5):138-44.

Camps J, Pashley D. In vivo sensitivity of human root dentin to air blast and scratching. Journal of Periodontology. 2003;74(11):1589-94.

Karpinia KA, Magnusson I, Sagel PA, Zhou X, Gerlach RW. Vital bleaching with two at-home professional systems. American Journal of Dentistry. 2002;15:18A.

Canullo L, Goglia G, Iurlaro G, Iannello G. Short-term bone level observations associated with platform switching in immediately placed and restored single maxillary implants: a preliminary report. International Journal of Prosthodontics. 2009;22(3).

Horowitz HS, Creighton WE, mcclendon BJ. The effect on human dental caries of weekly oral rinsing with a sodium fluoride mouthwash: a final report. Archives of Oral Biology. 1971;16(6):609-16.

Flath RK, Hicks ML, Dionne RA, Pelleu GB. Pain suppression after pulpectomy with preoperative flurbiprofen. Journal of Endodontics. 1987;13(7):339-47.

Palomo F, Wantland L, Sanchez A, Volpe AR, mccool J, devizio W. The effect of three commercially available dentifrices containing triclosan on supragingival plaque formation and gingivitis: a six month clinical study. International Dental Journal. 1994;44(1 Suppl 1):75-81.

Dunne SM, Hannington-Kiff JG. The use of topical guanethidine in the relief of dentine hypersensitivity: a controlled study. Pain. 1993;54(2):165-8.

Sculean A, Schwarz F, Chiantella GC, Donos N, Arweiler NB, Brecx M, et al. Five‐year results of a prospective, randomized, controlled study evaluating treatment of intra‐bony defects with a natural bone mineral and GTR. Journal of Clinical Periodontology. 2007;34(1):72-7.

Gisselsson H, Birkhed D, Emilson C-G. Effect of professional flossing with naf or snf2 gel on approximal caries in 13-16-year-old schoolchildren. Acta Odontologica Scandinavica. 1999;57(2):121-5.

Bratthall G, Sderholm G, Neiderud AM, Kullendorff B, Edwardsson S, Attstrm R. Guided tissue regeneration in the treatment of human infrabony defects Clinical, radiographical and microbiological results: a pilot study. Journal of Clinical Periodontology. 1998;25(11):908-14.

Varela VM, Heller D, Silva-Senem MX, Torres MCMB, Colombo APV, Feres-Filho EJ. Systemic antimicrobials adjunctive to a repeated mechanical and antiseptic therapy for aggressive periodontitis: a 6-month randomized controlled trial. Journal of Periodontology. 2011;82(8):1121-30.

Depaola PF, Soparkar M, Van Leeuwen M, develis R. The anticaries effect of single and combined topical fluoride systems in school children. Archives of Oral Biology. 1980;25(10):649-53.

Li Y, Lee SS, Cartwright SL, Wilson AC. Comparison of clinical efficacy and safety of three professional at-home tooth whitening systems. Compendium of Continuing Education in Dentistry (Jamesburg, NJ: 1995). 2003;24(5):60, 362, 4 passim; quiz 78.

Tecco S, Teté S, Crincoli V, Festa MA, Festa F. Fixed orthodontic therapy in temporomandibular disorder (TMD) treatment: an alternative to intraoral splint. CRANIO. 2010;28(1):30-42.

Li X, Tse HF, Yiu KH, Li LSW, Jin L. Effect of periodontal treatment on circulating CD34 cells and peripheral vascular endothelial function: a randomized controlled trial. Journal of Clinical Periodontology. 2011;38(2):148-56.

Strand P, Engquist B, Anzn B, Bergendal T, Hallman M, Karlsson U, et al. Nonsubmerged and submerged implants in the treatment of the partially edentulous maxilla. Clinical Implant Dentistry and Related Research. 2002;4(3):115-27.

Wan K, Jing Q, Zhao JZ. Evaluation of oral midazolam as conscious sedation for pediatric patients in oral restoration. Chinese Medical Sciences Journal. 2006;21(3):163-6.

Xajigeorgiou C, Sakellari D, Slini T, Baka A, Konstantinidis A. Clinical and microbiological effects of different antimicrobials on generalized aggressive periodontitis. Journal of Clinical Periodontology. 2006;33(4):254-64.

Allen DR, Davies R, Bradshaw B, Ellwood R, Simone AJ, Robinson R, et al. Efficacy of a mouthrinse containing 0.05% cetylpyridinium chloride for the control of plaque and gingivitis: a 6-month clinical study in adults. Compendium of Continuing Education in Dentistry

Molina MX, Rodriguez FG, Urbina T, Vargas S. Effect of weekly mouthrinses with 0.2% neutral naf solution on caries incidence in first permanent molars. Odontologia Chilena. 1989;37(1):176-82.

Goodson JM, Cugini MA, Kent RL, Armitage GC, Cobb CM, Fine D, et al. Multicenter evaluation of tetracycline fiber therapy: II. Clinical response. Journal of Periodontal Research. 1991;26(4):371-9.

Hashemi HM, Beshkar M, Aghajani R. The effect of sutureless wound closure on postoperative pain and swelling after impacted mandibular third molar surgery. British Journal of Oral and Maxillofacial Surgery. 2012;50(3):256-8.

Yun F, Firkova EI, Jun-Qi L, Xun H. Effect of non-surgical periodontal therapy on patients with type 2 diabetes mellitus. Folia Med (Plovdiv). 2007;49(1-2):32-6.

Negm MM. Effect of intracanal use of nonsteroidal anti-inflammatory agents on posttreatment endodontic pain. Oral Surgery, Oral Medicine, Oral Pathology. 1994;77(5):507-13.

Cayir Keles G, Ozkan Cetinkaya B, Albayrak D, Koprulu H, Acikgoz G. Comparison of platelet pellet and bioactive glass in periodontal regenerative therapy. Acta Odontologica Scandinavica. 2006;64(6):327-33.

Kamil W, Al Habashneh R, Khader Y, Al Bayati L, Taani D. Effects of nonsurgical periodontal therapy on C‐reactive protein and serum lipids in Jordanian adults with advanced periodontitis. Journal of Periodontal Research. 2011;46(5):616-21.

Fan X, Li X, Wan H, Hu D, Zhang YP, Volpe AR, et al. Clinical investigation of the anticaries efficacy of a 1.14% sodium monofluorophosphate (SMFP) calcium carbonate-based dentifrice: a two-year caries clinical trial on children in China. The Journal of Clinical Dentistry. 2008;19(4):134-7.

Yankell SL, Emling RC. A Thirty-Day Safety and Efficacy Evaluation of the Rowenta, Braun and Sonicare Powered Toothbrushes and a Manual Toothbrush. The Journal of Clinical Dentistry. 1996;8(4):120-3.

Haukeb K, Skaret E, st L-G, Raadal M, Berg E, Sundberg H, et al. One-vs. Five-session treatment of dental phobia: a randomized controlled study. Journal of Behavior Therapy and Experimental Psychiatry. 2008;39(3):381-90.

James PMC, Anderson RJ. Clinical testing of a stannous fluoride-calcium pyrophosphate dentifrice in Buckinghamshire school children. British Dental Journal. 1967;123:33-9.

Cortellini P, Prato GP, Tonetti MS. Periodontal regeneration of human intrabony defects with titanium reinforced membranes. A controlled clinical trial. Journal of Periodontology. 1995;66(9):797-803.

Yukna RA. Clinical evaluation of coralline calcium carbonate as a bone replacement graft material in human periodontal osseous defects. Journal of Periodontology. 1994;65(2):177-85.

Gordon JM, Lamster IB, Seiger MC. Efficacy of Listerine antiseptic in inhibiting the development of plaque and gingivitis. Journal of Clinical Periodontology. 1985;12(8):697-704.

Sadatmansouri S, Sedighpoor N, Aghaloo M. Effects of periodontal treatment phase I on birth term and birth weight. Journal of Indian Society of Pedodontics and Preventive Dentistry. 2006;24(1):23.

Clark GT, Blumenfeld I, Yoffe N, Peled E, Lavie P. A crossover study comparing the efficacy of continuous positive airway pressure with anterior mandibular positioning devices on patients with obstructive sleep apnea. Chest. 1996;109(6):1477-83.

Gardea MA, Gatchel RJ, Mishra KD. Long-term efficacy of biobehavioral treatment of temporomandibular disorders. Journal of Behavioral Medicine. 2001;24(4):341-59.

Needleman IG, Gerlach RW, Baker RA, Damani NC, Smith SR, Smales FC. Retention, antimicrobial activity, and clinical outcomes following use of a bioerodible tetracycline gel in moderate-to-deep periodontal pockets. Journal of Periodontology. 1998;69(5):578-83.

Trubman A, Crellin JA. Effect on dental caries of self-application of acidulated phosphate fluoride paste and gel. The Journal of the American Dental Association. 1973;86(1):153-7.

Koshy G, Kawashima Y, Kiji M, Nitta H, Umeda M, Nagasawa T, et al. Effects of single‐visit full‐mouth ultrasonic debridement versus quadrant‐wise ultrasonic debridement. Journal of Clinical Periodontology. 2005;32(7):734-43.

Ran F, Gedalia I, Fried M, Hadani P, Tved A. Effectiveness of fortnightly tooth brushing with amine fluorides in caries‐prone subjects. Journal of Oral Rehabilitation. 1991;18(4):311-6.

Yates R, Owens J, Jackson R, Newcombe RG, Addy M. A split‐mouth placebo‐controlled study to determine the effect of amorphous calcium phosphate in the treatment of dentine hypersensitivity. Journal of Clinical Periodontology. 1998;25(8):687-92.

Davies GM, Duxbury JT, Boothman NJ, Davies RM, Blinkhorn AS. A staged intervention dental health promotion program to reduce early childhood caries. Community Dental Health. 2005;22(2):118-22.

Okuda K, Momose M, Miyazaki A, Murata M, Yokoyama S, Yonezawa Y, et al. Enamel matrix derivative in the treatment of human intrabony osseous defects. Journal of Periodontology. 2000;71(12):1821-8.

Sowmya NK, Kumar ABT, Mehta DS. Clinical evaluation of regenerative potential of type I collagen membrane along with xenogenic bone graft in the treatment of periodontal intrabony defects assessed with surgical re-entry and radiographic linear and densitometric analysis. Journal of Indian Society of Periodontology. 2010;14(1):23.

Bashutski JD, Wang H-L, Rudek I, Moreno I, Koticha T, Oh T-J. Effect of flapless surgery on single-tooth implants in the esthetic zone: a randomized clinical trial. Journal of Periodontology. 2013;84(12):1747-54.

Hagan PP, Rozier RG, Bawden JW. The caries-preventive effects of full-and half-strength topical acidulated phosphate fluoride. Journal of Pediatric Dentistry. 1985;7(3):185-91.

Mansouri SS, Esteghamati A, Yousefi Y. Evaluation of first phase non-surgical periodontal therapy on diabetes control. Iranian Journal of Diabetes and Metabolism. 2006;6(1):101-6.

Hrzeler M, Fickl S, Zuhr O, Wachtel HC. Peri-implant bone level around implants with platform-switched abutments: preliminary data from a prospective study. Journal of Oral and Maxillofacial Surgery. 2007;65(7):33-9.

Driscoll WS, Swango PA, Horowitz AM, Kingman A. Caries-preventive effects of daily and weekly fluoride mouthrinsing in a fluoridated community: final results after 30 months. The Journal of the American Dental Association. 1982;105(6):1010-3

Wennström JL, Tomasi C, Bertelle A, Dellasega E. Full‐mouth ultrasonic debridement versus quadrant scaling and root planing as an initial approach in the treatment of chronic periodontitis. Journal of Clinical Periodontology. 2005;32(8):851-9.

Al-Zahrani MS, Austah ON. Photodynamic therapy as an adjunctive to scaling and root planing in treatment of chronic periodontitis in smokers. Saudi Medical Journal. 2011;32(11):1183-8.

Paolantonio M, Femminella B, Coppolino E, Sammartino G, D'Arcangelo C, Perfetti G, et al. Autogenous periosteal barrier membranes and bone grafts in the treatment of periodontal intrabony defects of single-rooted teeth: a 12-month reentry randomized controlled clinical trial. Journal of Periodontology. 2010;81(11):1587-95.

Holm G-B, Hoist K, Mejre I. The caries-preventive effect of a fluoride varnish in the fissures of the first permanent molar. Acta Odontologica Scandinavica. 1984;42(4):193-7.

Lundh H, Westesson P-L, Jisander S, Eriksson L. Disk-repositioning onlays in the treatment of temporomandibular joint disk displacement: comparison with a flat occlusal splint and with no treatment. Oral Surgery, Oral Medicine, Oral Pathology. 1988;66(2):155-62.

Muhler JC. Effect of a stannous fluoride dentifrice on caries reduction in children during a three-year study period. The Journal of the American Dental Association. 1962;64(2):216-24.

Axelsson P, Paulander J, Nordkvist K, Karlsson R. Effect of fluoride containing dentifrice, mouthrinsing, and varnish on approximal dental caries in a 3‐year clinical trial. Community Dentistry and Oral Epidemiology. 1987;15(4):177-80.

Rakprasitkul S, Pairuchvej V. Mandibular third molar surgery with primary closure and tube drain. International Journal of Oral and Maxillofacial Surgery. 1997;26(3):187-90.

Sgan-Cohen HD, Gat E, Schwartz Z. The effectiveness of an amine fluoride/stannous fluoride dentifrice on the gingival health of teenagers: results after six months. International Dental Journal. 1996;46(4):340-5.

Quirynen M, Avontroodt P, Peeters W, Pauwels M, Coucke W, Van Steenberghe D. Effect of different chlorhexidine formulations in mouthrinses on de novo plaque formation. Journal of Clinical Periodontology. 2001;28(12):1127-36.

Hanachowicz L. Caries prevention using a 1.2% sodium monofluorophosphate dentifrice in an aluminium oxide trihydrate base. Community Dentistry and Oral Epidemiology. 1984;12(1):10-6.

Weisenstein PR, Zacherl WA. A multiple-examiner clinical evaluation of a sodium fluoride dentifrice. The Journal of the American Dental Association. 1972;84(3):621-3.

Döri F, Huszár T, Nikolidakis D, Arweiler NB, Gera I, Sculean A. Effect of platelet‐rich plasma on the healing of intra‐bony defects treated with a natural bone mineral and a collagen membrane. Journal of Clinical Periodontology. 2007;34(3):254-61.

Zacherl WA. A three-year clinical caries evaluation of the effect of a sodium fluoride-silica abrasive dentifrice. Pharmacology and therapeutics in dentistry. 1980;6(1-2):1-7.

Demir B, Şengün D, Berberoğlu A. Clinical evaluation of platelet‐rich plasma and bioactive glass in the treatment of intra‐bony defects. Journal of Clinical Periodontology. 2007;34(8):709-15.

Holborow DW. A clinical trial of a potassium oxalate system in the treatment of sensitive root surfaces. Archives of Oral Biology. 1994;39:S134.

Pruthi VK, Perio C, Gelskey SC, Mirbod SM. Furcation therapy with bioabsorbable collagen membrane: a clinical trial. Journal-Canadian Dental Association. 2002;68(10):610-6.

Stookey GK, Mau MS, Isaacs RL, Gonzalez-Gierbolini C, Bartizek RD, Biesbrock AR. The relative anticaries effectiveness of three fluoride-containing dentifrices in Puerto Rico. Caries Research. 2004;38(6):542-50.

Theodoro LH, Silva SP, Pires JR, Soares GHG, Pontes AEF, Zuza EP, et al. Clinical and microbiological effects of photodynamic therapy associated with nonsurgical periodontal treatment. A 6-month follow-up. Lasers in Medical Science. 2012;27(4):687-93.

Eitner S, Bittner C, Wichmann M, Nickenig H-J, Sokol B. Comparison of conventional therapies for dentin hypersensitivity versus medical hypnosis. Intljournal of Clinical and Experimental Hypnosis. 2010;58(4):457-75.

Muzzin KB, Johnson R. Effects of potassium oxalate on dentin hypersensitivity in vivo. Journal of Periodontology. 1989;60(3):151-8.

Radnai M, Pal A, Novak T, Urban E, Eller J, Gorzo I. Benefits of periodontal therapy when preterm birth threatens. Journal of Dental Research. 2009;88(3):280-4.

Moses AN, Hollandsworth JG. Relative effectiveness of education alone versus stress inoculation training in the treatment of dental phobia. Behavior Therapy. 1985;16(5):531-7.

Eckles TA, Reinhardt RA, Dyer JK, Tussing GJ, Szydlowski WM, dubous LM. Intracrevicular application of tetracycline in white petrolatum for the treatment of periodontal disease. Journal of Clinical Periodontology. 1990;17(7):454-62.

Horowitz HS, Law FE, Thompson MB, Chamberlin SR. Evaluation of a stannous fluoride dentifrice for use in dental public health programs I. Basic findings. The Journal of the American Dental Association. 1966;72(2):408-22.

Hanna R, Trejo PM, Weltman RL. Treatment of intrabony defects with bovine-derived xenograft alone and in combination with platelet-rich plasma: a randomized clinical trial. Journal of Periodontology. 2004;75(12):1668-77.

Pilloni A, Paolantonio M, Camargo PM. Root coverage with a coronally positioned flap used in combination with enamel matrix derivative: 18-month clinical evaluation. Journal of Periodontology. 2006;77(12):2031-9.

Walsh M, Heckman B, Leggott P, Armitage G, Robertson PB. Comparison of manual and power toothbrushing, with and without adjunctive oral irrigation, for controlling plaque and gingivitis. Journal of Clinical Periodontology. 1989;16(7):419-27.

Deasy MJ, Singh SM, Rustogi KN, Petrone DM, Battista G, Petrone ME, et al. Effect of a dentifrice containing triclosan and a copolymer on plaque formation and gingivitis. Clinical Preventive Dentistry. 1991;13(6):12-9.

Radike AW, Gish CW, Peterson JK, King JD, Segreto VA. Clinical evaluation of stannous fluoride as an anticaries mouthrinse. The Journal of the American Dental Association. 1973;86(2):404-8.

Canullo L, Fedele GR, Iannello G, Jepsen S. Platform switching and marginal bone‐level alterations: the results of a randomized‐controlled trial. Clinical Oral Implants Research. 2010;21(1):115-21.

Altiere ET, Reeve CM, Sheridan PJ. Lyophilized bone allografts in periodontal intraosseous defects. Journal of Periodontology. 1979;50(10):510-9.

Melike Ordulu, Irem Aktas, Serhat Yalcin, Aysen Nekora Azak, Gulumser Evliogˇlu, Rian Disçi, Yusuf Emes, Comparative study of the effect of tube drainage versus methylprednisolone after third molar surgery, Oral Surgery, Oral Medicine, Oral Pathology, Oral Radiology, and Endodontology, Volume 101, Issue 6, 2006, Pages e96-e100, ISSN.2005.09.002.

Sunitha RV, Sapthagiri E. Flapless implant surgery: a 2-year follow-up study of 40 implants. Oral Surgery, Oral Medicine, Oral Pathology and Oral Radiology. 2013;116(4):e243.

Hughes JA, West NX, Parker DM, Newcombe RG, Addy M. Development and evaluation of a low erosive blackcurrant juice drink 3. Final drink and concentrate, formulae comparisons in situ and overview of the concept. Journal of dentistry. 1999;27(5):345-50.

Andersen R, Loebel N, Hammond D, Wilson M. Treatment of periodontal disease by photodisinfection compared to scaling and root planing. Journal of Clinical Dentistry. 2007;18(2):34.

Glass RL, Shiere FR. A clinical trial of a calcium carbonate base dentifrice containing 0.76% sodium monofluorophosphate. Caries Research. 1978;12(5):284-9.

Jervøe‐Storm PM, Semaan E, alahdab H, Engel S, Fimmers R, Jepsen S. Clinical outcomes of quadrant root planing versus full‐mouth root planing. Journal of Clinical Periodontology. 2006;33(3):209-15.

Ashley FP, Mainwaring PJ, Emslie RD, Naylor MN. Clinical testing of a mouthrinse and a dentifrice containing fluoride. A two-year supervised study in school children. British Dental Journal. 1977;143(10):333-8.

Ide M, mcpartlin D, Coward PY, Crook M, Lumb P, Wilson RF. Effect of treatment of chronic periodontitis on levels of serum markers of acute‐phase inflammatory and vascular responses. Journal of Clinical Periodontology. 2003;30(4):334-40.

Horowitz HS, Heifetz SB, mcclendon J, Viegas AR, Guimaraes LOC, Lopes ES. Evaluation of self-administered prophylaxis and supervised toothbrushing with acidulated phosphate fluoride. Caries Research. 1974;8(1):39-51.

Yilmaz HG, Cengiz E, Kurtulmus‐Yilmaz S, Leblebicioglu B. Effectiveness of Er, Cr: YSGG laser on dentine hypersensitivity: a controlled clinical trial. Journal of Clinical Periodontology. 2011;38(4):341-6.

Stavropoulos A, Karring T, Kostopoulos L. Fully vs. Partially rough implants in maxillary sinus floor augmentation: a randomized‐controlled clinical trial. Clinical Oral Implants Research. 2007;18(1):95-102.

Howat AP, Hollaway PJ, Davies TG. Caries prevention by daily supervised use of a MFP gel dentifrice. Report of a 3-year clinical trial. British Dental Journal. 1978;145(8):233.

Koch G, Petersson LG. Caries preventive effect of a fluoride‐containing varnish (Duraphat®) after 1 year's study. Community Dentistry and Oral Epidemiology. 1975;3(6):262-6.

Pizzo M, Zucchelli G, Modica F, Villa R, Debernardi C. Coronally advanced flap with or without enamel matrix derivative for root coverage: a 2‐year study. Journal of Clinical Periodontology. 2005;32(11):1181-7.

Tritten CB, Armitage GC. Comparison of a sonic and a manual toothbrush for efficacy in supragingival plaque removal and reduction of gingivitis. Journal of Clinical Periodontology. 1996;23(7):641-8.

Dri F, Huszar T, Nikolidakis D, Tihanyi D, Horvath A, Arweiler NB, et al. Effect of platelet-rich plasma on the healing of intrabony defects treated with Beta tricalcium phosphate and expanded polytetrafluoroethylene membranes. Journal of Periodontology. 2008;79(4):660-9.

Blumenthal NM. A clinical comparison of collagen membranes with e-PTFE membranes in the treatment of human mandibular buccal class II furcation defects. Journal of Periodontology. 1993;64(10):925-33.

Kielbassa AM, Martinez-de Fuentes R, Goldstein M, Arnhart C, Barlattani A, Jackowski J, et al. Randomized controlled trial comparing a variable-thread novel tapered and a standard tapered implant: interim one-year results. The Journal of Prosthetic Dentistry. 2009;101(5):293-305.

Zacherl WA. Clinical evaluation of an aged stannous fluoride-calcium pyrophosphate dentifrice. Journal of the Canadian Dental Association. 1972;38(4):155-7.

Sculean A, Pietruska M, Schwarz F, Willershausen B, Arweiler NB, Auschill TM. Healing of human intrabony defects following regenerative periodontal therapy with an enamel matrix protein derivative alone or combined with a bioactive glass. Journal of Clinical Periodontology. 2005;32(1):111-7.

Sun W-L, Chen L-L, Zhang S-Z, Wu Y-M, Ren Y-Z, Qin G-M. Inflammatory cytokines, adiponectin, insulin resistance and metabolic control after periodontal intervention in patients with type 2 diabetes and chronic periodontitis. Internal Medicine. 2011;50(15):1569-74.

Gerlach RW, Gibb RD, Sagel PA. A randomized clinical trial comparing a novel 5.3% hydrogen peroxide whitening strip to 10%, 15%, and 20% carbamide peroxide tray-based bleaching systems. Compendium of Continuing Education in Dentistry (Jamesburg, NJ: 1995)Supplement. 1999(29):3.

Pontoriero R, Wennstrm J, Lindhe J. The use of barrier membranes and enamel matrix proteins in the treatment of angular bone defects. A prospective controlled clinical study. Journal of Clinical Periodontology. 1999;26(12):833-40.

Okuda K, Tai H, Tanabe K, Suzuki H, Sato T, Kawase T, et al. Platelet-rich plasma combined with a porous hydroxyapatite graft for the treatment of intrabony periodontal defects in humans: a comparative controlled clinical study. Journal of Periodontology. 2005;76(6):890-8.

Lindhe J, Rosling B, Socransky SS, Volpe AR. The effect of a triclosan‐containing dentifrice on established plaque and gingivitis. Journal of Clinical Periodontology. 1993;20(5):327-34.

Lind OP, Möller IJ, Fher FR, Larsen MJ. Caries‐preventive effect of a dentifrice containing 2% sodium monofluorophosphate in a natural fluoride area in Denmark. Community Dentistry and Oral Epidemiology. 1974;2(2):104-13.

Schiff T, Dotson M, Cohen S, De Vizio W, mccool J, Volpe A. Efficacy of a dentifrice containing potassium nitrate, soluble pyrophosphate, PVM/MA copolymer, and sodium fluoride on dentinal hypersensitivity: a twelve-week clinical study. The Journal of Clinical Dentistry. 1993;5:87-92.

Enkling N, Jöhren P, Klimberg V, Bayer S, Mericske‐Stern R, Jepsen S. Effect of platform switching on peri‐implant bone levels: a randomized clinical trial. Clinical Oral Implants Research. 2011;22(10):1185-92.

Goyal CR, Qaqish J, He T, Grender J, Walters P, Biesbrock AR. A randomized 12-week study to compare the gingivitis and plaque reduction benefits of a rotation-oscillation power toothbrush and a sonic power toothbrush. Journal of Clinical Dentistry. 2009;20(3):93.

Abrams RG, Chambers DW. Caries-inhibiting effect of a stannous fluoride silica gel dentifrice: a three-year clinical study. Clinical Preventive Dentistry. 1980;2(1):22.

Krejci CB, Bissada NF, Farah C, Greenwell H. Clinical evaluation of porous and nonporous hydroxyapatite in the treatment of human periodontal bony defects. Journal of Periodontology. 1987;58(8):521-8.

Van Strydonck DAC, Timmerman MF, Van Der Velden U, Van Der Weijden GA. Plaque inhibition of two commercially available chlorhexidine mouthrinses. Journal of Clinical Periodontology. 2005;32(3):305-9.

Hughes JA, West NX, Parker DM, Newcombe RG, Addy M. Development and evaluation of a low erosive blackcurrant juice drink in vitro and in situ1. Comparison with orange juice. Journal of dentistry. 1999;27(4):285-9.

D’aiuto F, Nibali L, Parkar M, Suvan J, Tonetti MS. Short-term effects of intensive periodontal therapy on serum inflammatory markers and cholesterol. Journal of Dental Research. 2005;84(3):269-73.

Isaacs RL, Beiswanger BB, Rosenfield ST, Crawford JL, Mau MS, Eckert GJ, et al. A crossover clinical investigation of the safety and efficacy of a new oscillating/rotating electric toothbrush and a high frequency electric toothbrush. American Journal of Dentistry. 1998;11(1):7-12.

Blumenthal N, Steinberg J. The use of collagen membrane barriers in conjunction with combined demineralized bone-collagen gel implants in human infrabony defects. Journal of Periodontology. 1990;61(6):319-27.
